# Influence of platelet-rich plasma composition on pain and functional performance in knee osteoarthritis: a systematic review and network meta-analysis

**DOI:** 10.1186/s43019-026-00318-4

**Published:** 2026-04-21

**Authors:** Eduardo Anitua, Sabino Padilla, Roberto Prado, Roberto Tierno, Mohammad Hamdan Alkhraisat

**Affiliations:** 1 BTI Biotechnology Institute, Vitoria-Gasteiz, Spain; 2 University Institute for Regenerative Medicine and Oral Implantology, UIRMI (UPV/EHU-Fundación Eduardo Anitua), Vitoria-Gasteiz, Spain

**Keywords:** Platelet-rich plasma, Knee osteoarthritis, Systematic review, Network meta-analysis, Randomized controlled trials

## Abstract

**Background:**

Pain, decreased quality of life, and functional impairment are common symptoms of knee osteoarthritis (KOA), a degenerative joint disease. Surgery is reserved for advanced cases, and conservative treatment is primarily palliative. Although platelet-rich plasma (PRP) therapy is a novel regenerative strategy, the influence of PRP composition on its effectiveness remains unclear. The aim of this review is to determine whether PRP activation and platelet and leukocyte enrichment are associated with improved pain and functional outcomes in KOA at 6 and 12 months.

**Methods:**

The systematic review included 56 randomized controlled trials (RCTs), involving a total of 5251 patients. Of these, 53 RCTs involving 5031 participants were included in the network meta-analysis. PRP treatments were compared with other nonsurgical interventions and placebo. Primary outcomes included Western Ontario and McMaster Universities Arthritis Index Score (WOMAC), Knee Injury and Osteoarthritis Outcome Score (KOOS), and visual analog scale (VAS), while International Knee Documentation Committee Score (IKDC), Lequesne Index, and EuroQol (EQ)-VAS were assessed as secondary outcomes. PRP formulations were categorized on the basis of activation status and Mishra’s classification system. Both direct and indirect comparisons were performed using a frequentist network meta-analysis approach.

**Results:**

Comparing PRP with different activation states at 6 and 12 months revealed that PRP activation exerted significant benefits in specific KOOS domains at 12 months (KOOS Activities of Daily Living, KOOS Sport and Recreation Function, and KOOS Knee-Related Quality of Life). Generally, the performance of high-platelet PRP was not statistically different from that of low-platelet PRP in most of the assisted questionnaires and domains. Considering activated PRP, no significant variation was detected between Mishra’s categories, indicating that increased leukocyte and platelet enrichment ratios confer no additional benefit.

**Conclusions:**

Overall, the data suggest that PRP activation could play a key role in the treatment outcomes of KOA and could compensate for variation in both platelet and leukocyte enrichment. There is a need for RCTs to assess the effect of platelet composition and activation status in the clinical performance of PRP in KOA.

*Level of evidence:* Level I, systematic review and network meta-analysis.

**Supplementary Information:**

The online version contains supplementary material available at 10.1186/s43019-026-00318-4.

## Introduction

Knee osteoarthritis (KOA) is a prevalent degenerative joint disorder and a leading cause of disability worldwide [1]. Characterized by progressive deterioration of articular cartilage, synovial inflammation, and subchondral bone remodeling triggered by cumulative mechanical stress, KOA results in chronic pain, functional impairment, increased susceptibility to cardiovascular and atherosclerosis-related disorders, and reduced quality of life [2]. KOA predominantly affects older adults and is now recognized as one of the ten most disabling diseases in developed countries, leading to to significant functional limitations, with approximately 80% of affected individuals experiencing reduced mobility and an additional 25% reporting difficulties in performing activities of daily living [3]. This substantial clinical burden underscores the importance of designing targeted evidence-based therapeutic strategies specifically tailored to the affected population [4, 5]. According to current guidelines, surgical interventions in the management of KOA, such as arthroscopy, arthroplasty, osteotomy, and preservation surgery, are typically reserved for patients who have not responded adequately to primary conservative therapies and exhibit advanced joint degeneration accompanied by significant pain and restricted physical function [6–8]. Nevertheless, evidence from randomized controlled trials (RCTs) indicates that arthroscopic joint debridement provides limited long-term benefits in pain relief and functional improvement compared with conservative management [9]. Considering the diffuse and progressive nature of KOA, focal surgical interventions usually exhibit restricted effectiveness and involve risks associated with perioperative complications, such as superficial wound or periprosthetic joint infection and venous thromboembolism [10, 11].

Current nonsurgical treatment strategies—including physiotherapy, radiofrequency, nonsteroidal anti-inflammatory drugs (NSAIDs), intra-articular injections of corticosteroids (COR), hyaluronic acid (HA) or genicular nerve blocks, and extra-articular injections—provide only temporary symptomatic relief and have not demonstrated the ability to modify disease progression. This therapeutic limitation has driven increased attention toward regenerative medicine approaches, including gene therapy, laser therapy, prolotherapy, autologous microfragmented adipose tissue with stromal vascular fraction, or platelet-rich plasma (PRP) [12, 13]. Among these, PRP has emerged as a promising autologous blood-derived biologic therapy characterized by a high concentration of platelets that release growth factors and cytokines essential for tissue regeneration and inflammation modulation [14]. As highlighted in recent literature reviews, numerous RCTs have evaluated the efficacy of intra-articular PRP injections in patients with KOA over the past decade, with promising outcomes in terms of pain reduction, joint function improvement, range of motion enhancement, and mobility restoration [15, 16]. However, the results have been inconsistent, and the clinical relevance of the observed benefits remains controversial. Differences in PRP preparation methods, patient selection criteria, administration regimen, experimental design, control treatment, and outcome measures contribute to the variability observed across studies [17, 18]. In particular, variability in PRP manufacturing protocols—including centrifugation speed and time, anticoagulant use, plasma fractions collection or exogenous platelet activation—leads to significant heterogeneity in the final product’s volume, cellular composition, and biochemical profile, thus modulating PRP’s biological activity [19–21]. Consequently, numerous researchers have emphasized the importance of adopting and accurately reporting standardized PRP protocols to allow consistent comparison across studies and trustworthy assessment of clinical outcomes [20, 22, 23].

With the widespread implementation of PRP-based regenerative approaches in clinical practice and the unresolved questions regarding the relationship between PRP composition and its therapeutic efficacy, a comprehensive evaluation of the existing evidence is required. Thus, the primary objective of the present systematic review and network meta-analysis is to evaluate the effect of PRP composition on clinical efficacy and safety-related outcomes in patients with KOA. Clinical outcomes evaluated include pain reduction and functional improvement at 6 and 12 months assessed using patient-reported subjective scales, including the Western Ontario and McMaster Universities Osteoarthritis Index (WOMAC), the Knee injury and Osteoarthritis Outcome Score (KOOS), the visual analog scale (VAS), the International Knee Documentation Committee (IKDC) Subjective Knee Evaluation Form, the Lequesne Index, and the EuroQol visual analog scale (EQ-VAS). This review aims to establish whether PRP activation and platelet and leukocyte enrichment are associated with better pain management and improved functionality in patients with KOA after 6 and 12 months.

## Methods

Prior to study initiation, a detailed protocol describing the methodology for this systematic review and aggregate data meta-analysis was registered in the PROSPERO International Prospective Register of Systematic Reviews (registration no. CRD42025646778, version 18 February 2025 RPV and RTF, version 18 February 2025). The protocol was developed in accordance with the Preferred Reporting Items for Systematic Review and Meta-Analysis Protocols (PRISMA-P) guidelines [24].

### Search strategy

A systematic literature search was performed to identify RCTs published in English investigating the administration of PRP. Studies were eligible for inclusion without restriction based on the type of PRP used, PRP administration regimen, KOA clinical assessment or characteristics of the study population. A computer-assisted systematic search with no constraints on publication date or publication status was conducted in PubMed, EMBASE, Cochrane Central Register of Controlled Trials (CENTRAL), and SCOPUS databases, with monthly updates until 3 January 2025, using the following combination of terms using subject headings and variations on keywords selected to optimize both search sensitivity and specifi city: platelet-rich plasma, platelet concentrate, platelet gel, platelet, plasma, growth factors, plasma rich in growth factors, platelet-rich fi brin, platelet lysate, platelet releasate, osteoarthritis and knee. A detailed description of the systematic search strategy for each database is provided as Supplementary Material. Furthermore, the reference lists of key publications were manually screened to identify additional eligible studies not captured in the initial search. Prior to the screening process, duplicate records were removed by applying a combination of computer-assisted and manual verification methods to delete duplicate citations [25].

### Study selection and inclusion criteria

Two reviewers [blind for review] independently assessed the study list following a conventional double-screening approach, with disagreements resolved via discussion and third-reviewer arbitration by MHA if required [26]. Eligibility was initially assessed by screening titles and abstracts, with full-text review conducted when necessary. Studies were deemed eligible if PRP for KOA was referenced in either the title or abstract. Studies investigating all forms of KOA, including both primary and secondary KOA regardless of the radiographic grading, clinical phenotype, anatomical location, or extent of the osteoarthritis within the knee joint compartment, were included. Only full-text RCTs comparing PRP with at least one nonsurgical regenerative or non-regenerative treatment—such as physical therapy, NSAIDs or intra-articular (IA) injections of COR, HA, geniculate nerve blocks, microfragmented adipose tissue (MFAT), or bone marrow concentrate (BMC)—and/or placebo were considered in this review. The focus was on KOA treatments involving PRP administered as monotherapy irrespective of experimental design, PRP composition, administration regimen, KOA clinical status, or patient demographics. Inclusion was limited to studies with minimum follow-up duration of 6 months. Preclinical in vitro/in vivo studies, case reports and series, uncontrolled observational and prospective studies, retrospective studies, and narrative, scoping, or systematic reviews—with or without meta-analysis—were excluded.

### Study population

The study population consisted of individuals diagnosed with KOA, irrespective of disease severity, radiographic grading, clinical phenotype, anatomical location, or extent of the OA within the knee joint compartment, age, gender, or other demographic and clinical characteristics.

### Interventions

Intra-articular PRP injections delivered as monotherapy were compared against other regenerative or non-regenerative nonsurgical management therapies for KOA regardless of their administration route or mechanism of action.

### Definition of outcomes

All the RCTs identified via systematic literature search were examined to determine the clinical efficacy of PRP treatment for KOA. Primary outcomes included WOMAC [27, 28], KOOS [28], and VAS for pain [29]. For WOMAC and KOOS, both individual subscales—including WOMAC Pain, WOMAC Stiffness, WOMAC Physical Function, KOOS Pain, KOOS Activities of Daily Living, KOOS Sport and Recreation Function, KOOS Knee-Related Quality of Life, and KOOS Symptoms—and the global index (WOMAC Total and KOOS Total) were collected and analyzed when available. In cases where overall WOMAC score was not provided but individual WOMAC subscales were explicitly reported, a WOMAC global score was estimated by combining subscale-specific scores. Secondary outcomes included the IKDC Subjective Score [30], EQ-VAS [31], and Lequesne Index [32], at both 6 and 12 months. All outcome scores were extracted as continuous variables.

### Data collection and synthesis

The following data were independently extracted by two reviewers RPV and RTF from each study: first author, year of publication, PRP and comparator groups, number of participants, patient selection criteria, population demographics and clinical characteristics, KOA severity and clinical status, outcome measures, administration regimen of the respective groups, follow-up period, PRP composition, and main results (Table 1). Corresponding authors were contacted if further information or clarification was required.

**Table 1:** Basic information, demographics, outcomes, and follow-up of the included studies

Ref.	First author	Year	Country	Group (arm)	Patients (*n*)	Knees (*n*)	Female (%)^*^	Age (years)	BMI (kg/m^2^)	Level of KOA severity		Outcome measures‡	Follow-up points (months)
										Scale^†^	Grade		
[54]	Acosta-Olivo	2014	Mexico	PRP	21	21	NR	NR	NR	K–L	1	KOOS	2, 4, 6
				Acetaminophen	21	21	NR	NR	NR				
[55]	Anz	2022	USA	PRP	39	39	44	52.2 ± 12.4	27.9 ± 5.8	K–L	1–3	WOMAC, IKDC	1, 3, 6, 9, 12, 18, 24
				Bone marrow concentrate	45	45	40	55.8 ± 11.3	27.7 ± 5.0				
[56]	Arliani	2022	Brazil	PRP	14	14	79	62.8 ± 6.1	28.3 ± 2.9	K–L	2–3	WOMAC	1, 2, 3, 4, 6
				Hyaluronic acid	15	15	87	63.4 ± 5.0	28.1 ± 3.9				
[57]	Bansal	2021	India	PRP	64	64	39.1	64..4	24.9	K–L	1–3	WOMAC, IKDC, 6MW	1, 3, 6, 12
				Hyaluronic acid	68	68	38.2	65.8	25.3				
[58]	Baria	2022	USA	PRP	30	30	33.3	51.9 ± 2.4	31.0 ± 0.8	K–L	1–4	KOOS, VAS, Tegner	1, 3, 6
				MFAT	28	28	71.4	56.1 ± 1.7	31.0 ± 0.9				
[59]	Baria	2024	USA	PRP	23	23	30.4	52.8 ± 14.0	31.8 ± 4.7	K–L	1–4	KOOS, VAS, Tegner	1, 3, 6, 12
				MFAT	26	26	69.2	56.7 ± 7.8	31.2 ± 4.8				
[60]	Bennell	2021	Australia	PRP	144	144	59	62.2 ± 6.3	29.0 ± 3.7	K–L	2–3	KOOS, QoL-8, knee pain while walking, overall knee pain severity, MRI cartilage volume	2, 12
				Normal saline	144	144	58.3	61.6 ± 6.6	29.6 ± 4.5				
[61]	Buendía-López	2018	Spain	PRP	33	33	52	56.2 ± 3.0	24.9 ± 0.32	K–L	1–2	WOMAC, VAS	6, 12
				Hyaluronic acid	32	32	52	56.6 ± 2.9	24.9 ± 0.41				
				NSAID	33	33	53	57.4 ± 3.1	25.2 ± 0.48				
[62]	Chu	2022	China	PRP	308	308	60	53.9 ± 5.0	27.5 ± 3.2	K–L	1–3	WOMAC, IKDC, VAS	3, 6, 12, 24, 60
				Normal saline	302	302	58	54.5 ± 5.1	27.9 ± 3.6				
[63]	Ciapini	2023	Italy	PRP	20	20	50	60.2 (39–80)	25.5 (19–36)	K–L	2–3	WOMAC, VAS	3, 6
				Hyaluronic acid	20	20	50	60.2 (39–80)	25.5 (19–36)				
[64]	Cole	2017	USA	PRP	49	49	42.9	55.9 ± 10.4	27.4 ± 3.9	K–L	1–3	WOMAC, IKDC, VAS	1.5, 3, 6, 12
				Hyaluronic acid	50	50	60.0	56.8 ± 10.5	29.0 ± 6.4				
[65]	Dulic	2021	Servia	PRP	34	34	55.9	58.8 ± 11.2	28.47 ± 4.54	K–L	2–4	KOOS, WOMAC, IKDC	1, 3, 6, 9, 12
				Hyaluronic acid	30	30	56.7	59.4 ± 14.0	29.98 ± 5.24				
[66]	Duymus	2017	Turkey	PRP	41	41	97	60.4 ± 5.1	27.6 ± 4.6	K–L	2–3	WOMAC, VAS	1, 3, 6, 12
				Hyaluronic acid	40	40	97.4	60.3 ± 9.1	28.4 ± 3.6				
				Ozone	39	39	88.6	59.4 ± 5.7	27.6 ± 4.4				
[67]	Elik	2020	Turkey	PRP	30	30	96.7	61.3 ± 7.9	30.37 ± 4.47	K–L	1–3	VAS, WOMAC, SF-36	1, 6
				Normal saline	27	27	88.9	60.2 ± 6.8	30.70 ± 3.97				
[68]	Elksniņš-Finogejevs	2020	Latvia	PRP	20	20	25	66.4 ± 8.4	28.6 ± 5.0	K–L	2–3	IKDC, VAS, KSS	0.25, 1, 3, 6, 12
				Corticosteroid	20	20	25	70.2 ± 9.2	30.5 ± 5.8				
[69]	Filardo	2015	Italy	PRP	94	94	36.2	53.3 ± 13.2	26.6 ± 4.0	K–L	0–3	KOOS, IKDC, EQ-VAS, Tegner score	2, 6, 12
				Hyaluronic acid	89	89	41.6	57.6 ± 11.8	26.9 ± 4.4				
[70]	Forogh	2016	Iran	PRP	24	24	71	59.1 ± 7.0	28.9 ± 2.8	K–L	2–3	KOOS, VAS, 20MW test, ROM, flexion contracture	2, 6
				Corticosteroid	24	24	62	61.1 ± 6.7	29.2 ± 3.4				
[71]	Ghai	2019	India	PRP	20	20	75	49.8 ± 9.4	NR	K–L	1–2	WOMAC, VAS	0.5, 1.5, 3, 6
				Normal saline	20	20	75	49.8 ± 9.4	NR				
[72]	Görmeli	2017	Turkey	PRP	39	39	59	53.7 ± 13.1	28.7 ± 4.8	K–L	1–4	IKDC, EQ-VAS	6
				Hyaluronic acid	39	39	56.4	53.5 ± 14	29.7 ± 3.7				
				Normal saline	40	40	50	52.8 ± 12.8	29.5 ± 3.2				
[73]	Huang	2019	China	PRP	40	40	79.2	54.5 ± 1.2	25.23 ± 4.15	K–L	1–2	WOMAC, VAS	3, 6, 9, 12
				Hyaluronic acid	40	40	84.2	54.8 ± 1.1	24.51 ± 3.09				
				Corticosteroid	40	40	82.5	54.3 ± 1.4	24.56 ± 3.62				
[74]	Joshi Jubert	2017	Spain	PRP	35	35	65.71	65.6 ± 8.6	68 ± 7.17	K–L	3–4	KOOS, VAS, SF-36	1, 3, 6
				Corticosteroid	30	30	80	68 ± 7.2	30.98 ± 4.16				
[75]	Karaborklu	2024	Turkey	PRP	28	28	71.4	55 ± 7	29 ± 4	K–L	2–3	WOMAC, VAS, SF-12, 40-m fast-paced walk test, stair climbing test	1.5, 3, 6, 12
				Structured exercise program	28	28	78.6	58 ± 7	27 ± 4				
[76]	Kaszynski	2022	Poland	PRP	28	28	NR	57 ± 8	26 ± 3	K–L	2–3	KOOS, WOMAC, IKDC 2000, VAS, EQ‐5D	1, 3, 6, 12
				MFAT	26	26	NR	55 ± 8	27 ± 3				
[77]	Küçükakkaş	2022	Turkey	PRP	20	20	80	57.5 ± 10.6	29.8 ± 6.8	K–L	2–3	WOMAC, VAS, cartilage thickness	1, 6
				Hyaluronic acid	20	20	70	57.0 ± 10.1	28.9 ± 3.6				
[78]	Lamo de Espinosa	2021	Spain	PRP	34	34	35	59.8	NR	ICRS	2–4	KOSS, WOMAC, VAS	6
				Hyaluronic acid	35	35	46	64.6	NR				
[79]	Lana	2016	Brazil	PRP	36	36	80.6	60.9 ± 7	27.42 ± 6.89	K–L	1–3	WOMAC, VAS	1, 3, 6, 12
				Hyaluronic acid	36	36	91.7	60 ± 6.6	28.24 ± 8.77				
[80]	Lewis	2022	Australia	PRP (× 1)	47	47	57	55.1 ± 12.6	29.3 ± 6.7	K–L	0–2	KOOS, EQ-5D-5L, VAS	1.5, 3, 6, 12
				PRP (× 3)	27	27	67	59.4 ± 8.9	29.7 ± 6.1				
				Normal saline	28	28	57	60.1 ± 9.3	29.9 ± 5.5				
[81]	Li	2023	China	PRP	34	34	76.5	59.5 ± 8.2	25.56 ± 1.94	K–L	1–3	WOMAC, VAS	1, 3, 6, 12
				Hyaluronic acid	33	33	60.6	58.9 ± 10.1	25.83 ± 2.28				
[82]	Lin	2019	Taiwan	PRP	18	31	71	61.2 ± 13.1	23.98 ± 2.62	Ahlbäck	1–3	WOMAC, IKDC	1, 2, 6, 12
				Hyaluronic acid	18	29	66	62.5 ± 9.9	26.26 ± 2.99				
				Normal saline	17	27	63	62.2 ± 11.7	24.98 ± 3.12				
[83]	Louis	2018	France	PRP	24	24	41.7	53.2 ± 11.7	25.6 ± 2.9	K–L	2–4	WOMAC, VAS, satisfaction	1, 3, 6
				Hyaluronic acid	24	24	54.2	48.5 ± 11.5	27.0 ± 2.9				
[84]	Malanin	2017	Rusia	PRP	30	30	53.3	49.3 ± 11.0	29.13 ± 2.68	K–L	2	Lequesne, VAS	1, 3, 6
				Hyaluronic acid	30	30	66.7	44.5 ± 8.9	27.77 ± 2.34				
[85]	Montanez-Heredia	2016	Spain	PRP	27	27	55.6	66.3 ± 8.3	29.0 ± 5.5	K–L	1–3	KOOS, VAS, EQ-Pain, EQ-5D	3, 6
				Hyaluronic acid	26	26	65.4	61.5 ± 8.6	30.4 ± 4.9				
[86]	Nunes-Tamashiro	2022	Brazil	PRP	34	34	88.2	67.6 ± 7.4	29.22 ± 3.2	K–L	2–3	WOMAC, VAS, SF-36, TUG, 6MW, Likert, % improvement	1, 2, 3, 12
				Corticosteroid	33	33	90.9	65.8 ± 6.1	29.59 ± 4.5				
				Normal saline	33	33	90.9	68 ± 6.2	30.23 ± 4.1				
[87]	Park	2021	Republic of Korea	PRP	55	55	70.9	60.6 ± 8.2	25.5 ± 2.2	K–L	1–3	WOMAC, IKDC, VAS	1.5, 3, 6
				Hyaluronic acid	55	55	85.5	62.3 ± 9.6	25.9 ± 2.8				
[88]	Patel	2013	India	PRP (× 1)	26	52	59.3	53.1 ± 11.6	26.28 ± 3.23	Ahlbäck	1–2	WOMAC, VAS	1.5, 3, 6
				PRP (× 2)	25	50	80	51.64 ± 9.2	25.81 ± 3.31				
				Normal saline	23	43	73.9	53.7 ± 8.2	26.21 ± 2.93				
[89]	Raeissadat	2020	Iran	PRP	50	50	72	57.1 ± 7.3	27.92 ± 2.7	K–L	2–3	WOMAC, VAS, Lequesne	2, 6, 12
				Hyaluronic acid	52	52	71.2	58.6 ± 7.1	28.65 ± 3.02				
[90]	Raeissadat	2015	Iran	PRP	77	77	89.6	56.9 ± 9.1	28.20 ± 4.63	K–L	1–4	WOMAC, SF-36	12
				Hyaluronic acid	62	62	75.8	61.1 ± 7.5	27.03 ± 4.15				
[91]	Reyes-Sosa	2020	Spain	PRP	30	30	86	53.7 ± 8.6	NR	K–L	2–3	WOMAC, VAS	1, 3, 6, 12
				NSAID	30	30	70	52.8 ± 9.6	NR				
[92]	Sánchez	2012	Spain	PRP	89	89	52	60.5 ± 7.9	27.9 ± 2.9	Ahlbäck	1–3	WOMAC, Lequesne, responders OMERACT-OARSI, acetaminophen	1, 2, 6
				Hyaluronic acid	87	87	52	58.9 ± 8.2	28.2 ± 2.7				
[93]	Sdeek	2021	Egypt	PRP	95	95	84.2	60.2	27.9	K–L	2–3	WOMAC, IKDC, VAS	2, 6, 12, 18, 24, 30, 36
				Hyaluronic acid	94	94	83	59.5	27.1				
[94]	Simental-Mendia	2016	Mexico	PRP	33	33	67	57.2 ± 8.1	32.2 ± 6.2	K–L	1–2	WOMAC, VAS, SF-12	1.5, 3, 6
				Acetaminophen	32	32	62	55.6 ± 11.4	29.5 ± 3.8				
[95]	Singh	2022	India	PRP	35	35	68.6	53.2 ± 7.8	28.88 ± 2.50	K–L	2–3	WOMAC, VAS	3, 6, 9
				Arthroscopy	35	35	62.9	54.8 ± 5.7	28.09 ± 3.03				
[96]	Smith	2016	USA	PRP	15	15	77	29.5 ± 6.9	29.53 ± 6.89	K–L	2–3	WOMAC	0.25, 0.5, 2, 3, 6, 12
				Normal saline	15	15	60	46.6 ± 9.4	27.47 ± 4.78				
[97]	Spakova	2012	Slovakia	PRP	60	60	45	52.8 ± 12.4	27.9 ± 4.1	K–L	1–3	WOMAC, VAS	3, 6
				Hyaluronic acid	60	60	48.3	53.2 ± 14.5	28.3 ± 4.0				
[98]	Su	2018	China	PRP	25	25	56	54.2 ± 6.6	28.17 ± 1.43	K–L	2–3	WOMAC, VAS, satisfaction	1, 3, 6, 12, 18
				Hyaluronic acid	30	30	60	53.1 ± 6.4	28.69 ± 1.13				
[99]	Tschopp	2023	Switzerland	PRP	24	30	42.9	62 (56–68)	26 (21.3–28.7)	K–L	1–3	WOMAC, pain NRS, Tegner activity scale	0.25, 3, 6, 9, 12, 15, 21, 24
				Contrast	23	30	40	58 (54–61)	25.1 (23–28.8)				
				Corticosteroid	24	30	52	59 (49–65)	27 (24.4–30.8)				
				Hyaluronic acid	24	30	37.5	64 (54.75–72)	25.5 (22.7–30.3)				
[100]	Tucker	2021	USA	PRP	11	11	27.3	57.5 ± 1.8	47.7 ± 8.9	K–L	2–3	WOMAC, VAS	0.3, 3, 6 12
				Normal saline	6	6	66.7	57.2 ± 3.9	29.1 ± 2.1				
[101]	Uslu Güvendi	2018	Turkey	PRP (× 1)	19	19	94.7	62.3 ± 1.6	31.4 ± 0.7	K–L	3	WOMAC, VAS, Lequesne	2, 6
				PRP (× 3)	14	14	92.9	60.4 ± 1.7	31.0 ± 1.0				
				Corticosteroid	17	17	88.2	62.8 ± 1.7	31.1 ± 1.0				
[102]	Vaquerizo	2013	Spain	PRP	48	48	66.7	62.4 ± 6.6	30.7 ± 3.6	K–L	2–4	WOMAC, Lequesne, responders OMERACT-OARSI	6, 12
				Hyaluronic acid	48	48	45.8	64.8 ± 7.7	31.0 ± 4.6				
[103]	Wang	2022	Taiwan	PRP	56	56	77.8	61.9 ± 5.5	24.07 ± 3.35	K–L	1–2	WOMAC	1, 3, 6
				Hyaluronic acid	54	54	71.4	63.0 ± 5.3	24.02 ± 2.39				
[104]	Wu	2018	Taiwan	PRP	20	20	75	63.3 ± 6.8	24.14 ± 2.93	Ahlbäck	1–2	WOMAC, isokinetic parameters	0.5, 1, 3, 6
				Hyaluronic acid	20	20	75	63.3 ± 6.8	24.14 ± 2.93				
[105]	Xu	2021	China	PRP	30	40	66.7	56.9 ± 4.2	22.5 ± 2.3	K–L	2–3	WOMAC, VAS, Lequesne, Lysholm	1, 6, 12, 24
				Hyaluronic acid	20	34	75.0	57.1 ± 3.4	22.8 ± 2.1				
[106]	Yaradilmis	2020	Turkey	L-PRP	30	30	86.7	60.3 ± 7.6	31.27 ± 4.08	K–L	2–3	WOMAC, VAS	2, 6, 12
				P-PRP	30	30	86.7	58.9 ± 6.3	32.53 ± 6.25				
				Hyaluronic acid	30	30	86.7	63.0 ± 9.2	32.4 ± 4.2				
[107]	Yoshioka	2024	Japan	PRP	15	15	80	65.9 ± 8.0	24.0 ± 3.3	K–L	2–3	WOMAC, VAS	1, 3, 6
				Normal saline	15	15	60	67.9 ± 10.7	22.9 ± 1.4				
[108]	Yurtbay	2022	Turkey	PRP (× 1)	62	62	38.9	53.3 ± 13.0	31.09 ± 5.52	K–L	1–3	KOOS, VAS, Kujala Patellofemoral Score, ROM, KC	1, 3, 6, 12, 24
				Normal saline (× 1)	59	59	18.6	56.3 ± 10.5	30.67 ± 4.51				
				PRP (× 3)	63	63	14.3	57.4 ± 8.8	30.68 ± 4.63				
				Normal saline (× 3)	53	53	34.0	53.5 ± 11.3	29.22 ± 4.79				
[109]	Zaffagnini	2022	Italy	PRP	55	55	34.5	54.1 ± 10.6	28.0 ± 5.5	K–L	1–4	KOOS, IKDC, EQ-VAS, EQ-5D, VAS	1, 3, 6, 12, 24
				MFAT	53	53	47.2	54.5 ± 12.1	25.9 ± 4.3				

^*^Not reported

^†^Kellgren–Lawrence

‡ 6MW: 6 Min Walk Test; 20MW: 20 Meter Walk Test; EQ-5D: EuroQol 5-Dimension; EQ-5D-5L: EuroQol 5-Dimension 5-Level questionnaire; NRS: Numeric Rating Scale; OMERACT-OARSI: Outcome Measures in Rheumatology from the Osteoarthritis Research Society International; ROM: Range of Motions; SF-12: 12-Item Short Form Health Survey; SF-36: 36-Item Short Form Health Survey; TUG: Timed Up and Go Test

### Risk of bias assessment

The risk of bias for each included study was independently evaluated by two reviewers RPV and RTF using the Revised Cochrane Risk of Bias Tool (RoB 2) for randomized controlled trials [33]. Discrepancies were resolved through discussion to reach consensus. If consensus could not be achieved, a third reviewer MHA was consulted [34].

### Publication bias assessment

Contour-enhanced funnel plots were generated to assess potential publication bias [35, 36]. Egger’s test for intercept was performed to quantify funnel plot asymmetry [37]. When significant asymmetry was detected and data from at least ten studies were available, Duval and Tweedie’s trim-and-fill method was applied to adjust for potential publication bias [38]. These analyses were conducted using a frequentist approach, with consideration of the inherent limitations in bias assessment within complex network meta-analyses.

### Statistical analysis

Aggregate data from individual RCTs were synthesized for each quantitative outcome. For studies reporting follow-up data at multiple time points, only data from follow-up time points at 6 and 12 months were included in the analyses. Continuous outcome measurements were expressed as endpoint measurements or baseline-to-follow-up absolute changes to provide adjusted estimates of the treatment effect while minimizing bias from randomization imbalances. In cases where standard deviations for absolute changes from baseline and/or follow-up time points were not reported, they were derived from medians, ranges, interquartile ranges (IQRs), and sample sizes using the approximation methodologies suggested by Wan et al. [39]. The standard deviation (SD) for baseline-to-follow-up change scores was estimated from 95% confidence intervals (CI), *p*-values, or *t*-values as suggested by Higgins et al. [40]. In studies reporting multiple frequencies, doses, or formulations of the same treatment or comparator, these arms were aggregated using a weighted mean and pooled standard deviation, in accordance with Cochrane guidelines for combining groups in meta-analysis [40]. When data on baseline and follow-up measurements or baseline-to-follow-up change scores were only displayed graphically (e.g. box plots, bar plots, or line plots), approximations for means and SDs were extracted via WebPlotDigitizer [41].

A frequentist network meta-analysis was conducted to compare the effect of exogenous platelet activation during PRP manufacturing process using the netmeta package (version 3.2.0) [42] in the R statistical computing environment (version 4.5.1) [43]. The activation status of PRP was systematically extracted from each publication. Where activation procedures were not explicitly reported, the PRP was classified as nonactivated for simplicity [44]. Strictly speaking, this term specifically denotes the absence of exogenous triggers. However, such preparations are more accurately described as endogenously (or not exogenously) activated [45], as platelets undergo physiological degranulation only upon in vivo contact with native tissues, collagen type I, endogenous thrombin, or synovial factors [46, 47].

To further explore potential sources of heterogeneity, PRP formulations were categorized and node splitting was conducted according to Mishra’s PRP classification system (Table 2; Supplementary Table S3) on the basis of leukocyte content, activation status, and platelet enrichment [48]. This framework provides a standardized nomenclature to differentiate PRP formulations and facilitate comparison of biological and clinical effects across studies. Platelet and leukocyte enrichment ratios were preferentially extracted from the original publications when explicitly reported (Supplementary Table S4). If these were unavailable, manufacturer-provided specifications were utilized. When neither source was available, the platelet content was estimated by applying reported platelet and leukocyte concentrations in PRP to baseline platelet counts in whole blood as provided by the study authors or manufacturers. In cases where baseline hematological parameters were not quantified, platelet counts were approximated using normative reference data derived from a large cohort of 1430 individuals of both sexes [49] and similarly to Magalon et al. [45]. When needed, leukocyte and erythrocyte concentrations in whole blood were extrapolated according to data from the third National Health and Nutrition Examination Survey [50]. All analyses were conducted in accordance with methodological recommendations for frequentist network meta-analyses.

**Table 2:** Classification of platelet-rich plasma formulations according to Mishra et al.

Type	Leukocyte content	Exogenous activation	Platelet concentration	
			≥ 5 × baseline	< 5 × baseline
Type 1	Increased	No	Type 1A	Type 1B
Type 2	Increased	Yes	Type 2A	Type 2B
Type 3	Minimal/absent	No	Type 3A	Type 3B
Type 4	Minimal/absent	Yes	Type 4A	Type 4B

Mishra et al. [48]

Effect estimates are reported as mean differences with corresponding 95% confidence intervals. Treatment effects were modeled using a random-effects model incorporating both within-study variance and between-study heterogeneity. The between-study variance (*τ*^2^) was estimated using the restricted maximum likelihood (REML) method under the assumption of a common heterogeneity parameter across all treatment comparisons [51]. The structure and connectivity of the evidence network were evaluated both graphically and quantitatively using network geometry metrics, including mean path length (defined as the average shortest number of steps between any two nodes) or minimal parallelism (reflecting the smallest number of direct comparisons connecting two treatments). Inconsistency between direct and indirect evidence was assessed using the design-by-treatment interaction model and node-splitting approach where applicable [52, 53].

## Results

### Literature search results and study selection

**Fig. 1:**
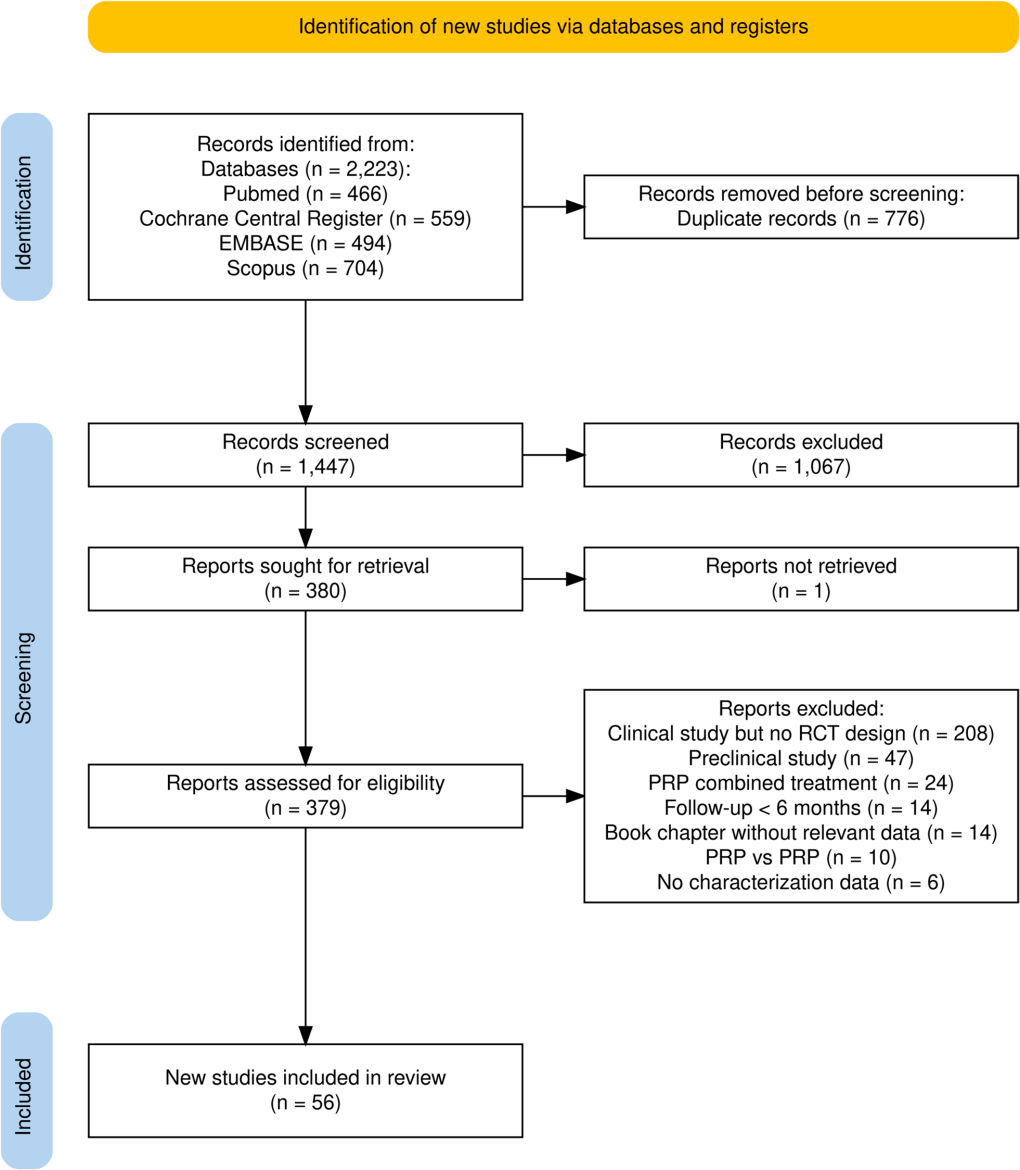
Preferred Reporting Items for Systematic reviews and Meta-Analyses (PRISMA) 2020 flow diagram

As shown in Fig. 1, a total of 2223 potentially relevant citations were identified as a result of the systematic search. After removing duplicates and screening the titles and abstracts for relevance, 379 full-text unique studies were assessed for eligibility. Of these, 56 studies [54–109] met the predefined inclusion criteria, and 53 were included in the quantitative synthesis [54–78, 80–84, 86–98, 100–109]. Table 1 outlines the main characteristics of the included studies. The pooled study population consisted of 5251 participants with symptomatic KOA, with a sex distribution of 39% male and 61% female in the PRP group, and 38% male and 62% female across the comparator groups. The overall pooled mean age of participants receiving PRP therapy was 57 years, while the mean age across the comparator groups was 58 years. However, a small number of RCTs did not report demographic data, including sex distribution [54, 76] and the mean age of the study population [54].

PRP administration regimen varied from a single infiltration [55, 57–59, 61, 65, 68, 70, 71, 74, 77, 80, 83, 86–88, 95, 99–101, 103, 104, 108, 109] to multiple injections of two [66, 88–91, 98] or three infiltration series [56, 60, 62–64, 67, 72, 73, 75, 76, 78–82, 84, 85, 92–94, 96, 101, 102, 105–108], with frequencies ranging from one [56, 60, 62, 64, 67, 69, 72, 78, 80–82, 84, 92, 96, 97, 101, 102, 106, 107], 2 [54, 76, 79, 85, 91, 93, 94, 98, 105], or three injections per week [73, 88, 89] to one injection per month [66, 90, 108] when explicitly stated. In contrast, comparator administration methods were adapted to the particular properties of each control treatment.

In terms of comparators, 15 studies utilized placebo [60, 62, 67, 72, 80, 82, 86, 88, 96, 99, 100, 104, 107, 108], while 40 employed active control groups [54–59, 61, 63–66, 68–70, 72–79, 81, 83–87, 89–95, 97–99, 101–103, 105, 106, 109]. These included 29 studies comparing PRP with HA [56, 57, 61, 63–65, 69, 71–73, 77–79, 81, 83–85, 87, 89, 90, 92, 93, 97–99, 102, 105, 106], 6 studies comparing PRP with COR [68, 70, 73, 74], 4 studies comparing PRP with MFAT [58, 59, 76, 109], 2 studies comparing PRP with acetaminophen [54, 94], 2 studies comparing PRP with NSAID [61, 91], 1 study comparing PRP with arthroscopy (ARTHRO) [95], 1 study comparing PRP with BMC [55], 1 study comparing PRP with ozone therapy (OZO) [66], and 1 study comparing PRP with structured exercise program (SEP) [75]. Despite the fact that the majority of studies were designed as two-arm clinical trials, a subset included multiple intervention and/or comparator groups [61, 66, 72, 73, 86, 99]. Only a small number of studies evaluated different dosing regimens, administration protocols, or PRP formulations [88, 101, 106].

### Risk of bias assessment

**Fig. 2:**
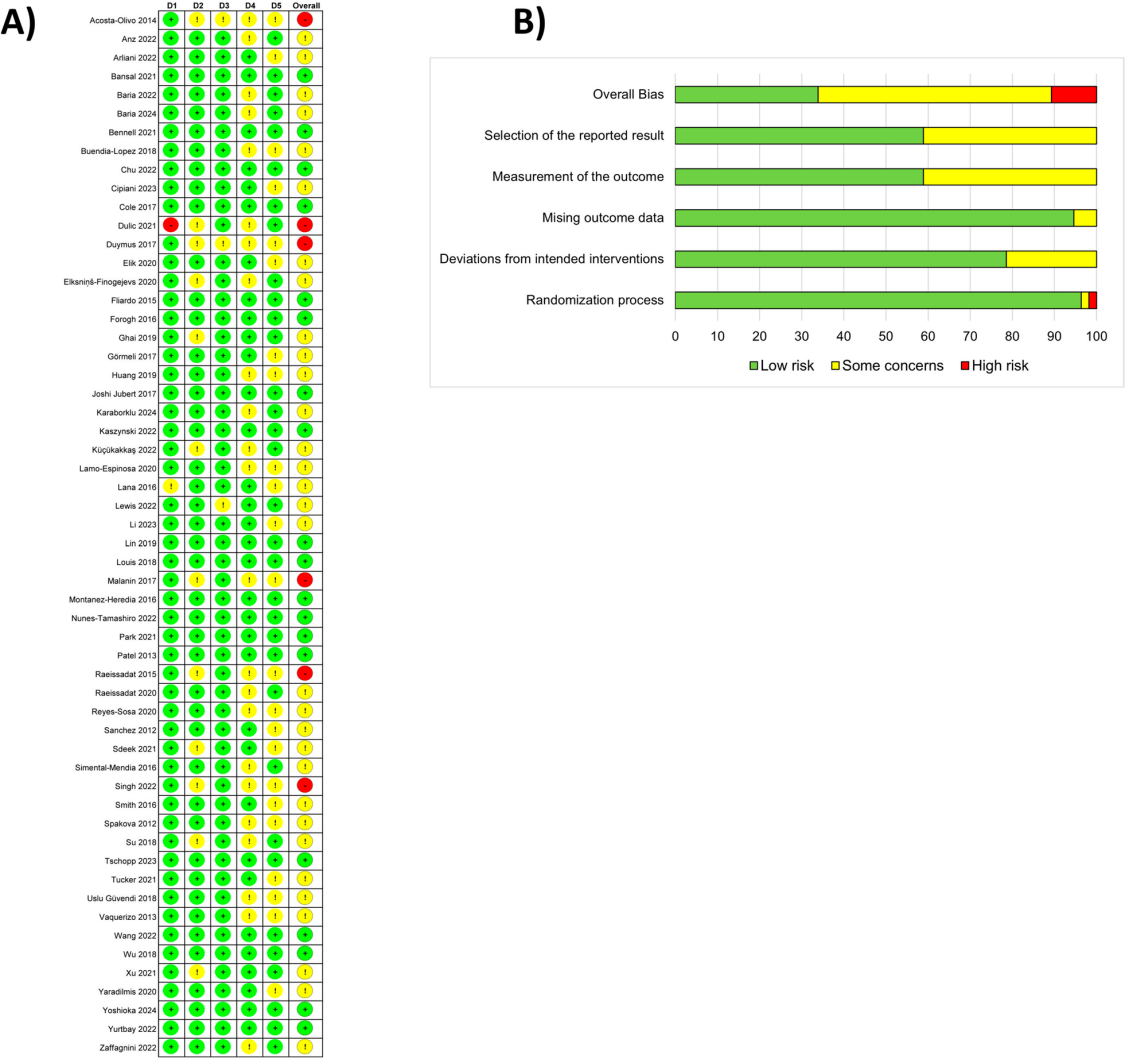
Risk of bias across the studies included in the systemic review: **A** traffic-light plot depicting the risk of bias for each domain across individual studies, and **B** summary plot representing the proportion of studies classified as low risk, with some concerns, or high risk of bias within each domain. This figure presents the review authors’ judgements for each risk-of-bias domain (D1, randomization process; D2, deviations from the intended interventions; D3, missing outcome data; D4, measurement of the outcome; and D5, selection of the reported result), as assessed using the RoB2 tool. Colors indicate risk of bias: green, low; yellow, some concerns; red, high

Figure 2 summarizes the risk of bias assessments for the studies included in the present systematic review. Low risk of bias in randomization was observed in 54 studies, considering that the randomization methodology was sufficiently detailed [54–64, 66–78, 80–95, 97–109]. In addition, another publication mentioned that the randomization process was conducted but the specific method employed was not clearly reported [79]. Finally, another study was considered to have a high risk of bias due to the absence of details on allocation or randomization procedures [65]. Regarding bias due to deviations from intended interventions, a total of 44 studies were classified as low risk [55–64, 67, 69, 70, 72–76, 78–83, 85–89, 91, 92, 94, 96, 97, 99–104, 106–109], whereas 12 studies raised some concerns [54, 65, 66, 68, 71, 77, 84, 90, 93, 95, 98, 105]. A low risk of bias related to missing outcome data was identified in 53 studies [55–65, 67–79, 81–109]. In contrast, three studies raised some concerns in this domain owing to substantial missing data [54, 66, 80]. Moreover, a total of 23 studies apparently did not blind patients and/or investigators. Considering that all primary and secondary outcomes relevant to the present review were subjectively assessed, these studies were rated as having some concerns about bias related to outcome measurement [54, 55, 58, 59, 61, 65, 66, 68, 73, 75, 77, 78, 84, 89–91, 94, 95, 97, 98, 101, 102, 109]. On the other hand, the remaining 33 studies were considered to have a low risk of bias since double blinding was conducted [56, 57, 60, 62–64, 67, 69–72, 74, 76, 79, 81–83, 85–88, 92, 93, 96, 99, 100, 103–108]. Regarding selective reporting bias, most studies (43) were judged to be at low risk, with clearly reported results [55, 57–60, 62, 64, 65, 68–71, 74–77, 80, 82, 83, 85–89, 94, 98, 99, 103, 105, 107–109]. However, up to 23 studies raised some concerns because their reporting of analysis results was unclear [54, 56, 61, 63, 66, 67, 72, 73, 78, 79, 81, 84, 90–93, 95–97, 100–102, 106]. Individual classifications for each domain were synthesized to generate a global risk of bias score. As a result, 19 studies were identified as having low overall risk of bias [57, 60, 62, 64, 69, 70, 74, 76, 82, 83, 85–88, 99, 103, 104, 107, 108], 31 studies were rated as having some concerns [55, 56, 58, 59, 61, 63, 67, 68, 71, 72, 75, 77–81, 89, 91–93, 96–98, 101, 102, 104–106, 109], and 6 were classified as showing high overall risk of bias [54, 65, 66, 84, 90, 95]. The proportion of these studies classified as high risk compared with the total number of studies included in the meta-analysis for each primary outcome was as follows: WOMAC Total 4/37, WOMAC Pain 2/27, WOMAC Stiffness 2/25, WOMAC Function 2/25, VAS 2/35, KOOS Pain 1/12, KOOS Symptoms 0/11, KOOS Function 0/11, KOOS Sports 0/11, KOOS Knee 0/11, and KOOS Total 2/4.

### Network meta-analyses quality assessment

The statistical synthesis was based on data from 5031 participants enrolled in 53 RCTs. To assess the robustness of the comparisons performed, different diagnostic tools were computed, including direct and indirect effect estimates to evaluate inconsistency, the proportion of direct evidence, and also path length and minimal parallelism as key network geometry metrics (Supplementary Material). Egger’s test yielded statistically nonsignificant results for both primary or secondary outcomes, regardless of the time point considered, indicating an absence of significant publication bias (*p* > 0.05). The diagnostic plots and regression tests for asymmetry are presented in the Supplementary Material.

### PRP activation

**Fig. 3:**
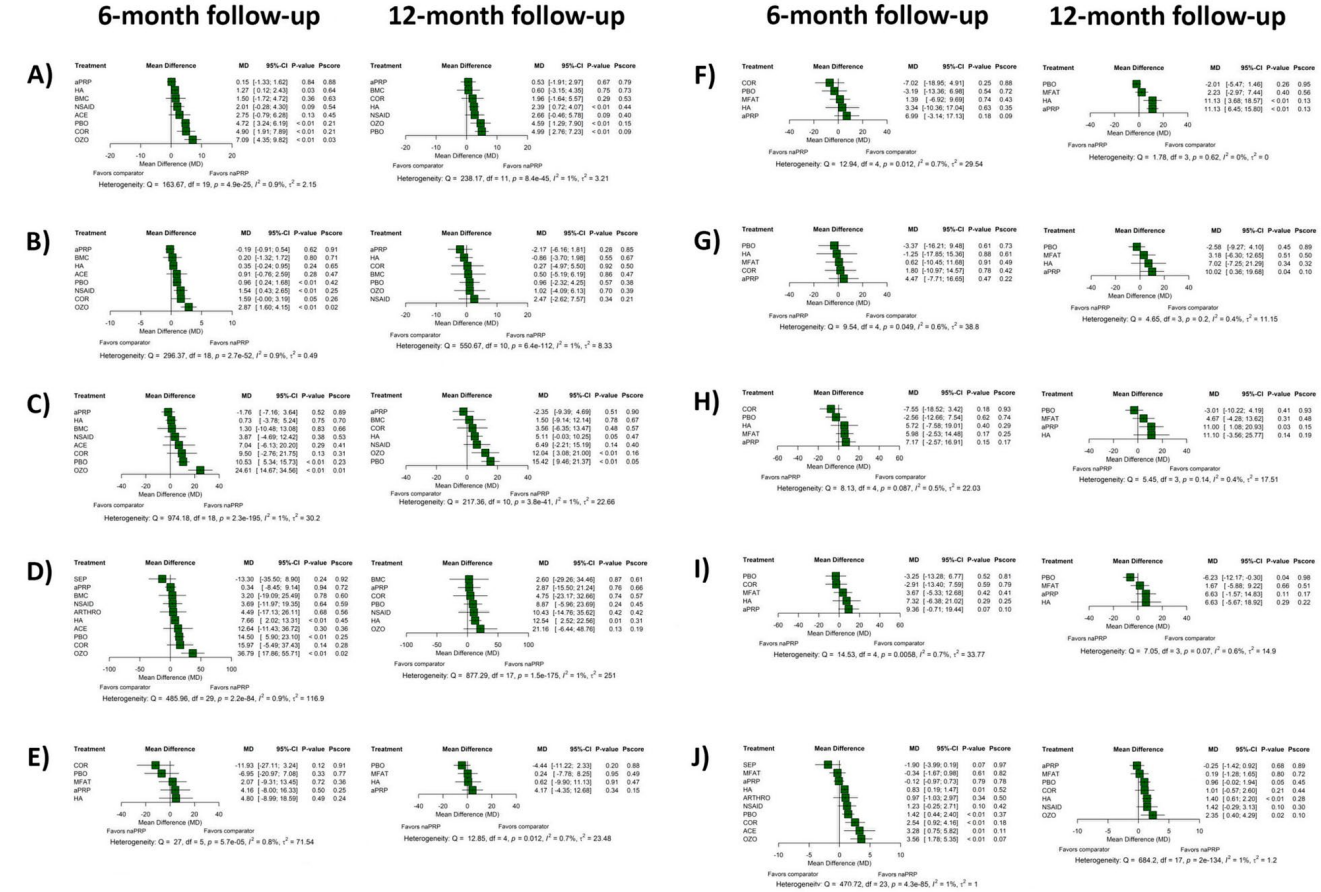
Forest plot of network meta-analysis results comparing the effect of different activation states of PRP, using nonactivated platelet-rich plasma (naPRP) as the reference comparator across primary outcomes: **A** WOMAC Pain, **B** WOMAC Stiffness, **C** WOMAC Physical Function, **D** WOMAC Total, **E** KOOS Pain, **F** KOOS Activities of Daily Living, **G** KOOS Sport and Recreation Function, **H** KOOS Knee-Related Quality of Life, **I** KOOS Symptoms, **J** KOOS Total, and **K** VAS after 6 and 12 months. Effect sizes are presented as mean differences (MD) with 95% confidence intervals (CI). As specified in each figure, the relative position of effect estimates favoring naPRP—to the left or to the right of the vertical line—was determined by the specific configuration of each clinical outcome scale. Reported *p*-values correspond to treatment comparisons versus the reference comparator. *p*-Scores were included to indicate the relative ranking and certainty of each treatment within the network

**Fig. 4:**
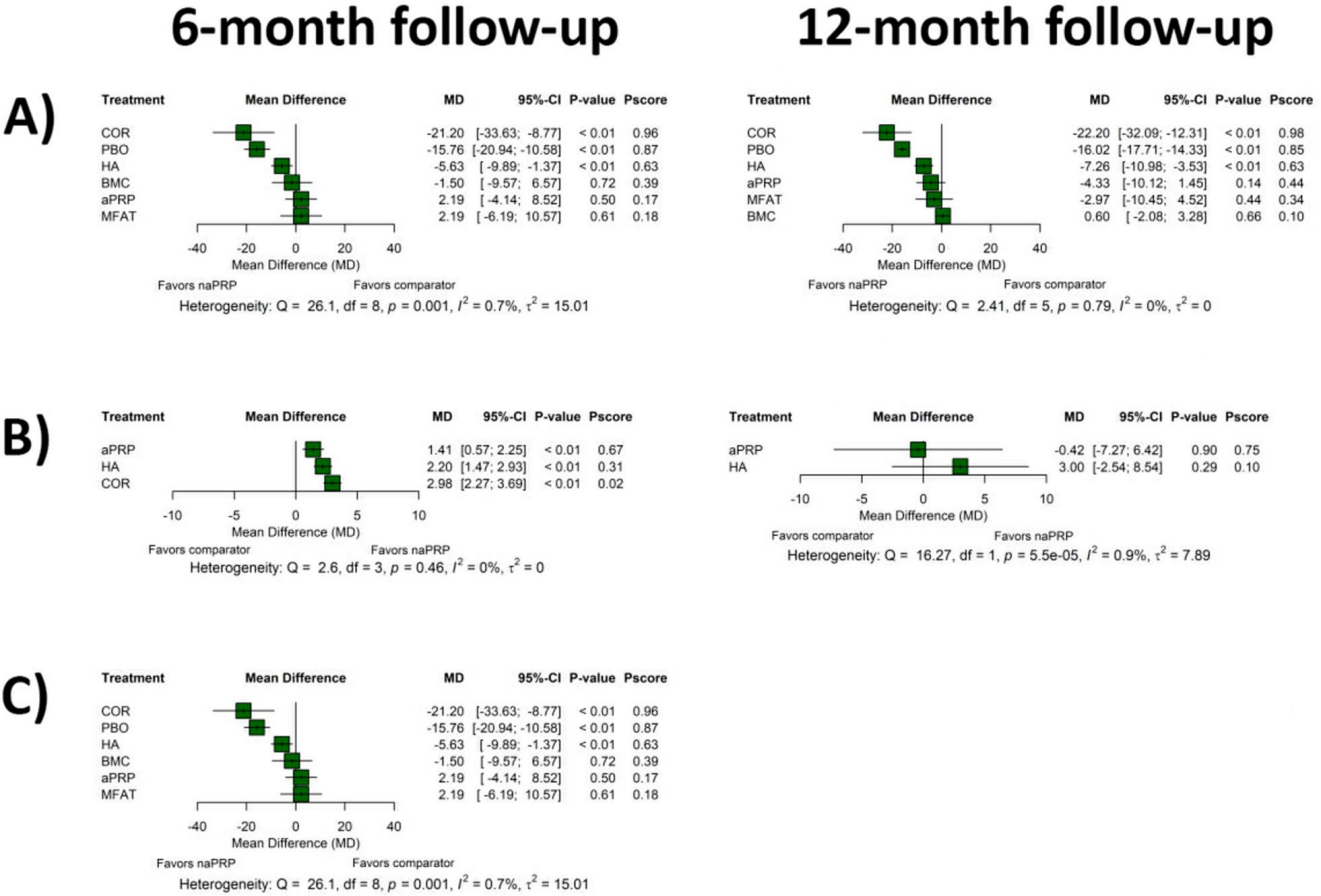
Forest plot of network meta-analysis results comparing the effect of different activation states of PRP, using nonactivated platelet-rich plasma (naPRP) as the reference comparator across secondary outcomes: **A** IKDC, **B** Lequesne Index, and **C** EQ-VAS after 6 and 12 months. Effect sizes are presented as mean differences (MD) with 95% confidence intervals (CI). As specified in each figure, the relative position of effect estimates favoring naPRP—to the left or to the right of the vertical line—was determined by the specific configuration of each clinical outcome scale. Reported *p*-values correspond to treatment comparisons versus the reference comparator. *p*-Scores were included to indicate the relative ranking and certainty of each treatment within the network

As revealed by the network meta-analytic synthesis, no differences in most primary or secondary outcomes—WOMAC Pain (*p* = 0.84), WOMAC Stiffness (*p* = 0.62), WOMAC Physical Function (*p* = 0.52), WOMAC Total (*p* = 0.94), KOOS Pain, KOOS Activities of Daily Living (*p* = 0.18), KOOS Sport and Recreation Function (*p* = 0.47), KOOS Symptoms (*p* = 0.068), KOOS Knee-Related Quality of Life (*p* = 0.15), VAS (*p* = 0.79), and IKDC (*p* = 0.17)—were detected between PRP formulations on the basis of activation status at the 6-months follow-up time point (Figs. 3 and 4). Conversely, PRP exogenous activation was associated with improved KOOS scores after 12 months, particularly in the KOOS Activities of Daily Living (*p* ≤ 0.01), KOOS Sport and Recreation Function (*p* = 0.042), and KOOS Knee-Related Quality of Life (*p* = 0.030) subscales. For the KOOS Activities of Daily Living and KOOS Knee-Related Quality of Life subscales, the observed changes were not only statistically significant but also exceeded the established minimal clinically important differences (MCID) of 9.2 and 10.3, respectively [110]. However, no differences were identified between activated and nonactivated PRP concerning WOMAC (WOMAC Pain: *p* = 0.92; WOMAC Stiffness: *p* = 0.26; WOMAC Physical Function *p* = 0.54; WOMAC Total: *p* = 0.56), KOOS Pain (*p* = 0.34), KOOS Symptoms (*p* = 0.11), VAS (*p* = 0.68), or IKDC (*p* = 0.14) after 12 months (Figs. 3 and 4). Results and relevant data from meta-analyses stratified by PRP activation status can be consulted in Supplementary Material.

### Mishra’s classification

**Fig. 5:**
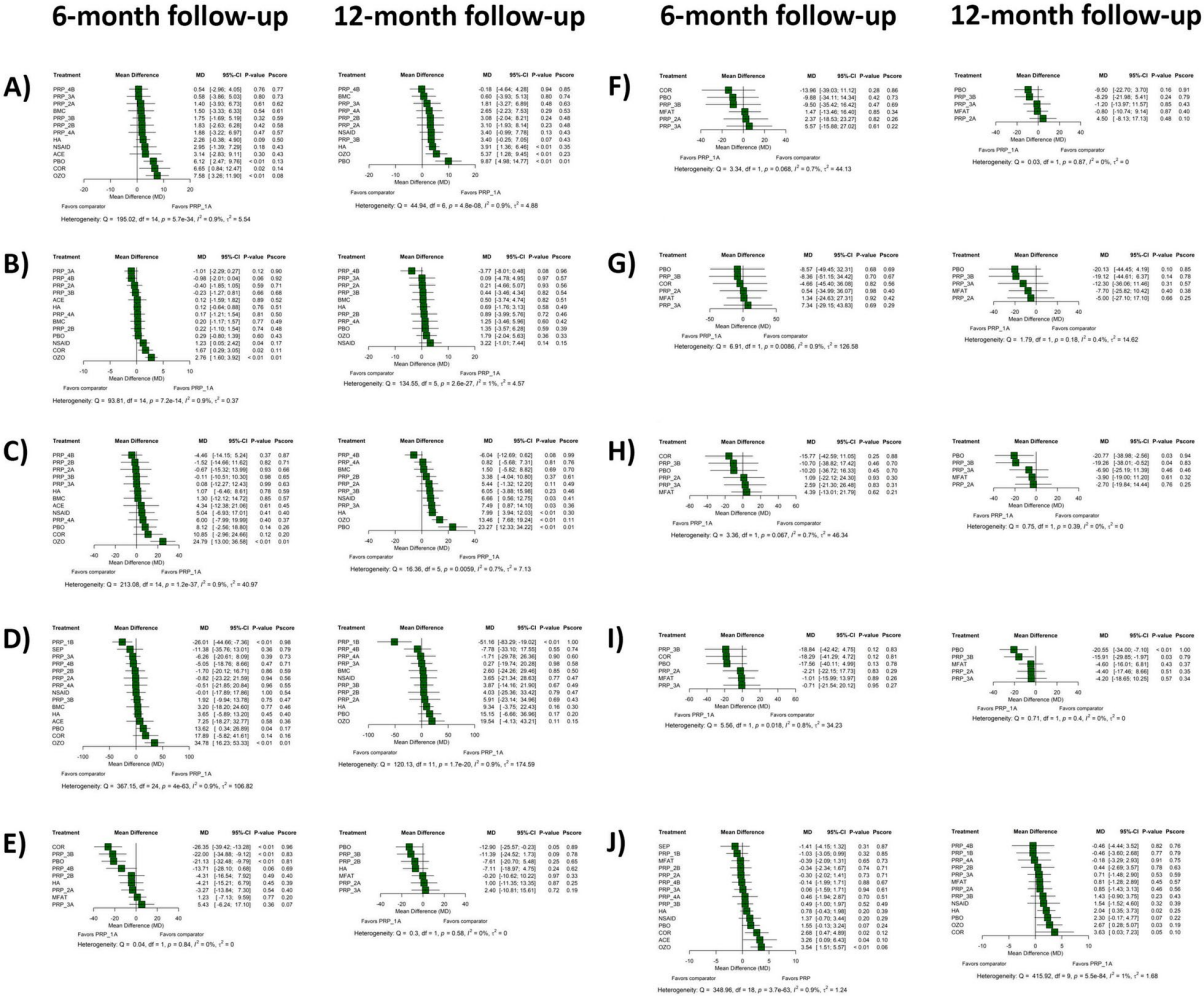
Forest plot of network meta-analysis results comparing the effect of different types of PRP as categorized by Mishra’s classification system, using nonactivated platelet rich and leukocyte rich platelet-rich plasma (1A) as the reference comparator across primary outcomes: **A** WOMAC Pain, **B** WOMAC Stiffness, **C** WOMAC Physical Function, **D** WOMAC Total, **E** KOOS Pain, **F** KOOS Activities of Daily Living, **G** KOOS Sport and Recreation Function, **H** KOOS Knee-Related Quality of Life, **I** KOOS Symptoms, **J** KOOS Total, and **K** VAS after 6 and 12 months. Effect sizes are presented as mean differences (MD) with 95% confidence intervals (CI). As specified in each figure, the relative position of effect estimates favoring PRP type 1A—to the left or to the right of the vertical line—was determined by the specific configuration of each clinical outcome scale. Reported *p*-values correspond to treatment comparisons versus the reference comparator. *p*-Scores were included to indicate the relative ranking and certainty of each treatment within the network

Effect size estimates for primary outcome scales computed via network meta-analysis are graphically represented in Fig. 5. No significant effect of PRP type was detected on any WOMAC subscale regardless of the time point considered. However, significantly lower overall WOMAC scores indicating improved pain and function-related outcomes were only detected in the comparison of leukocyte-rich and platelet-low nonactivated PRP (1B) with leukocyte-rich and platelet-rich non-activated PRP (1A) at both the 6-month and the 12-month follow up (*p* ≤ 0.01). At 6 months, the observed differences between the above-mentioned Mishra categories exceed the MCID for WOMAC Overall (6.4), indicating that, considering nonactivated leukocyte-rich PRPs, interventions based on platelet-low PRPs produced clinically relevant improvements in patient-reported outcomes at this time point when compared with platelet-rich PRPs [111]. On the other hand, no significant differences were observed when compared with leukocyte-poor formulations (types 3 and 4). Moreover, leukocyte-rich and platelet-rich non-activated PRP (1A) was associated with higher KOOS scores relative to leukocyte-poor and platelet-low non-activated PRP (3B) across specific KOOS domains, including KOOS pain at 6 months (*p* ≤ 0.01), and also KOOS Knee-Related Quality of Life (*p* = 0.044) and KOOS Symptoms at 12 months (*p* = 0.025). At the 6-month follow-up, the observed mean differences between Mishra categories 1A and 3B exceed the MCID reported for KOOS Pain (9.3). At 12 months, these intergroup differences surpassed the MCID thresholds for KOOS Knee-Related Quality of Life (10.3) and KOOS Symptoms (8.2), suggesting that the observed improvements are both statistically robust and clinically meaningful [110]. However, these differences disappeared with leukocyte-poor and platelet-low activated PRP (type 4B).

**Fig. 6:**
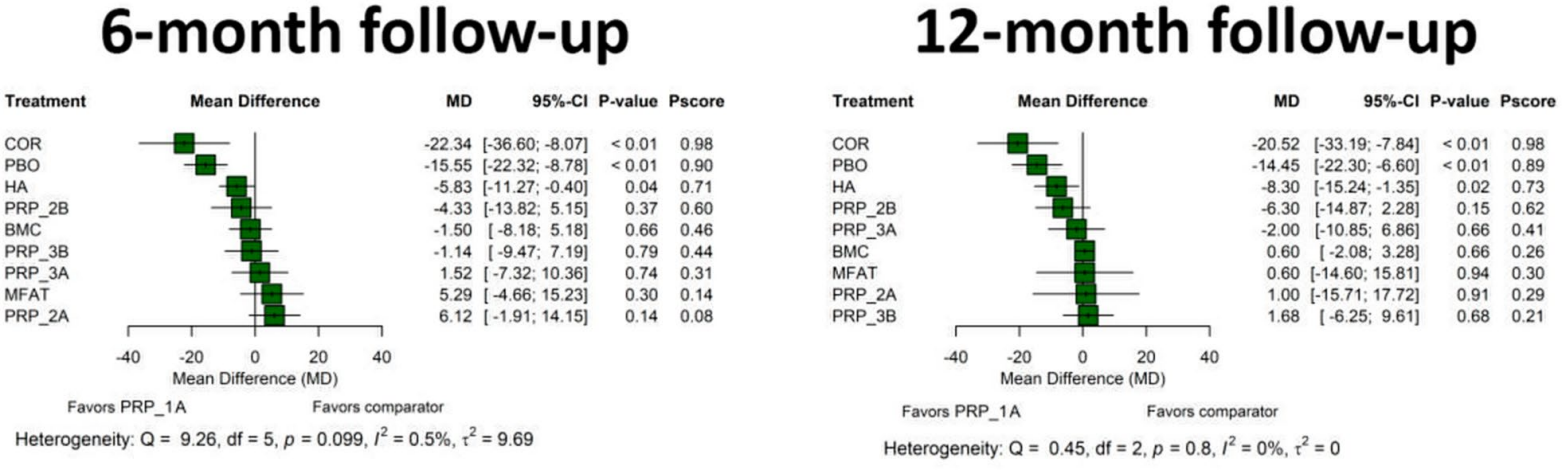
Forest plot of network meta-analysis results using non-activated platelet rich and leukocyte rich platelet-rich plasma (1A PRP according to Mishra’s classification system) as the reference comparator for IKDC after 6 and 12 months. Effect sizes are presented as mean differences with 95% confidence intervals (CI). As specified in each figure, the relative position of effect estimates favoring PRP type 1A—to the left of the vertical line—was determined by the specific configuration of IKDC scale. Reported *p*-values correspond to treatment comparisons versus the reference comparator. *p*-Scores were included to indicate the relative ranking and certainty of each treatment within the network

Regarding VAS scale, no statistically significant differences were observed between Mishra’s categories (Table 2) at 6 months or 12 months follow-up (*p* ≥ 0.05). Among the secondary outcomes (Fig. 6), between-group differences in IKDC were not statistically significant after comparing PRP treatment types based on Mishra’s classification regardless of the time point considered (*p* ≥ 0.05). Nevertheless, network meta-analysis could only be performed for the IKDC scores after categorizing PRP-based treatments by Mishra’s classification system. This limitation can be attributed to the small number of studies reporting Lequesne Index and EQ-VAS outcomes, which prevented the comparison of multiple PRP arms and reduced shared comparators across studies, ultimately leading to poor network connectivity. It should be noted that the number of studies providing direct evidence for comparisons between PRP types is minimal. As a consequence, most of the evidence resulting from network meta-analyses was derived indirectly via comparisons across multiple independent studies. In most cases, this led to a sparse network structure with a significant dependence on indirect evidence, which suggests that the results should be interpreted with caution.

## Discussion

This systematic review and network meta-analysis included 56 RCTs involving 5251 participants, exploring the effect of PRP composition on clinical outcomes in KOA, with particular reference to platelet concentration, leukocyte content, and activation status. The main findings of this study suggest that PRP exogenous activation was associated with improvements in KOOS scores for Sport and Recreation Function, Activities of Daily Living, and Knee-Related Quality of Life after 12 months, with the latter two exceeding the MCID [110]. In addition, variations in platelet concentration in activated PRP did not exhibit a statistically significant influence on functional and pain-related outcomes at 6 and 12 months. Our findings suggest that overall platelet concentration may represent a relevant compositional parameter that significantly influences the efficacy of nonactivated PRP but does not critically influence clinical outcomes following PRP exogenous activation. The functional profile of activated PRP is substantially different from that of its nonactivated counterpart. This divergence may reflect differences in the temporal dynamics and mechanisms of growth factor release, which critically modulates its biological activity and therapeutic potential [112, 113]. Compositional heterogeneity could also be a primary factor influencing the biological and physical properties of PRP formulations [114–116].

To date, a limited number of systematic reviews addressing the effect of PRP activation on its clinical efficacy have been published. The present systematic review includes 57 PRP arms, of which 66.7% do not perform exogenous activation, 31.6% activate with calcium derivatives, mainly calcium chloride, and only 1 study (1.7%) activates without calcium, using autologous thrombin. As outlined in the “Results” section, PRP activation leads to improvements across several domains of the KOOS scale (12 months). These results are in line with a recent systematic review and meta-analysis published by Simental-Mendía et al. [44], which included 14 RCT and concluded that exogenously activated PRP is more clinically effective than nonactivated PRP in improving pain and functional scores in patients with KOA. In contrast, studies using nonactivated PRP did not demonstrate significant pain relief or functional improvement compared with placebo [44]. Although PRP activation has been frequently described in individual studies, systematic reviews and meta-analyses generally do not account for this factor separately [117–120].

Several systematic reviews have examined the impact of platelet concentration on the effectiveness of PRP for treating KOA [111, 120–122], musculoskeletal disorders [119], and tennis elbow [123]. However, their conclusions were not primarily derived from a statistical comparison between different types of PRPs via network meta-analytic approaches that integrate evidence from both direct and indirect sources. Conversely, several studies have performed subgroup analysis by classifying the PRPs into multiple categories. Bensa et al. classified PRP formulations into two different categories on a platelet concentration basis: low: < 800,000 platelets/μL and high: ≥ 800,000 platelets/μL [111]. On the other hand, Bagheri et al. classified PRP according to the platelets, activation, white cell classifi cation system (PAW) classification system [122], whereas Berrigan et al. divided PRP formulations into three categoriess (platelet dosages < 5 billion, 5–10 billion, and > 10 billion) [119]. Recently, Oeding et al. defined high-dose PRP as the one with platelet enrichment factor of 3 or documented supraphysiological platelet dose [123]. The number of studies included in each subgroup varied both across PRP types and among systematic reviews—reaching up to two in some cases—and only Berrigan et al. [119] required a minimum of three studies to synthesize subgroups. Furthermore, comparators were not consistent across studies or systematic reviews. Isolation of the platelet concentration from other factors that could affect the biological properties of the PRP—i.e., centrifugation parameters, leukocytes content and profile, activation status, and other relevant variables—could constrain the ultimate objective of defining the optimal PRP compositional characteristics. The evaluation tools that have been used to quantify and compare the performance of the PRP formulations are validated and standardized questionnaires designed to record patient-reported outcomes [124]. However, differences are observed across these tools in terms of the domains covered and scoring criteria. Collectively, these factors have hindered the identification of a platelet concentration that optimizes PRP function.

The role of leukocytes in PRP, and especially for KOA treatment, is a complex and debated topic with contradictory findings across systematic reviews. The present analyses did not find significant evidence to support leukocyte enrichment in activated PRP as an effect modifier. For instance, Abbas et al. in 2022 [125] performed a network meta-analysis to explore the effect of WBC concentration on PRP infiltration for KOA. They concluded that there was no significant difference considering any outcome measures or local adverse reactions between LP-PRP and LR-PRP, including SUCRA rankings demonstrating a preference for LP-PRP. Similarly, Corsini et al. [126] suggested that LP-PRP, particularly with reduced neutrophil content, demonstrated superior efficacy in the KOA treatment, leading to better scores and pain relief than LR-PRP. In the same way, a systematic review and meta-analysis conducted by Kim et al. [118] and including 32 studies comparing the effect of PRP in KOA observed no significant differences in clinical outcomes between LP-PRP and LR-PRP, but the infiltration with LR-PRP was associated with a significantly higher risk of local adverse reactions compared to LP-PRP, specifically pain and swelling. Conversely, a systematic review conducted by Riboh et al. [117] found no significant differences in the incidence of local adverse reactions between LR-PRP and LP-PRP. However, the above-mentioned systematic review only included nine studies, of which only six were RCTs [117].

To address the limited number of clinical studies that directly assess PRP formulations with different composition and manufacturing properties, several studies [117, 121, 125] have adopted the advanced statistical technique of network meta-analysis to combine direct and indirect evidence within a single analytical framework inferred through a network of interventions, allowing the comparison of different treatment alternatives for a condition, even when no direct comparisons have been conducted in individual studies [127]. Yu et al. [121] recently conducted a network meta-analysis involving 23 RCTs, in which the treatment efficacy in KOA was measured only using the WOMAC scale. The authors classified PRP protocols according to four variables, leukocyte concentration (poor versus rich), platelet enrichment relative to WB (< 3-fold, 3–5-fold, and ≥ 5-fold), the use of anticoagulants, and PRP exogenous activation, generating 14 categories. The comparator groups consisted of HA and saline. Considering WOMAC function, statistically significant differences were observed, favoring leukocyte-poor and moderate-increase PRP (short term), leukocyte-rich and moderate-to-high PRP, and leukocyte-poor and low-to-high PRP (medium term). For WOMAC pain, statistically significant differences have been detected favoring leukocyte-rich and low-to-high PRP and leukocyte-poor and moderate-to-high PRP (short term), and high PRP (leukocyte-poor or leukocyte-rich) (medium term). For WOMAC stiffness, statistically significant differences have been observed for leukocyte-rich and low PRP (short term) and leukocyte-poor and high PRP (medium term). However, no significant effects were reported in the long term for any of the subscales studied [121]. This underscores the influence of the type of patient-reported outcome measure on the characteristics of PRP associated with significant positive effects.

The present comprehensive review included 56 RCT comprising 69 comparisons, thus allowing the authors to conduct a network meta-analysis that incorporates a substantially larger number of studies than previous reviews [111, 119–123, 126]. To the best of our knowledge, this is the first time that Mishra’s classification [48] has been used in a systematic review to analyze the most effective categories for treating KOA. Mishra’s classification system categorizes PRP into eight groups based on leukocyte content (higher or lower than the peripheral whole blood (WB) baseline), platelet content (higher or lower than five times the WB value), and activation status [48]. Although there are at least nine classifications of PRP [128], Mishra’s was the only one that could be used in this review to classify PRP formulations as a consequence of deficient reporting. This system allowed us to classify 55 out of 57 types of PRP (96.5%). Although the classification proposed by Kon et al. in 2020 is more comprehensive [129], it could not be used to categorize PRP formulations owing to insufficient description of their composition and characteristics.

The inclusion of PRP characterization variables in systematic reviews incorporates specific methodological challenges. Lim et al. [21] recently reported that only 12.1% of studies with level I and II evidence in orthopedics adequately described the methods used to obtain PRP. These data show a slight increase of just over 2% compared with a 2017 systematic review by Chahla et al. [23], which also identified that PRP collection methods and subsequent characterization were poorly described, thereby making it impossible in many cases to compare studies. Although expert consensus groups emphasize that PRP is a valid treatment option for KOA, especially for mild to moderate grades, they also highlight the importance of achieving standardization of product characteristics because the optimal parameters remain largely undefined [130–133]. Thus, platelet concentration should be routinely measured and reported for quality control and research purposes. However, the ideal dose or the optimal platelet concentration range for KOA treatment is currently undetermined, and there is a lack of strong consensus on how to define PRP using a specific threshold. To further reinforce this observation, a recent meta-analysis concluded that the administered platelet dose does not significantly influence the effect of PRP therapy for KOA. These findings were supported by both subgroup analyses and meta-regression [134]. Regarding leukocytes, is generally accepted that their concentration needs to be categorized for reporting purposes. However, expert consensus opinion is contradictory. While the Biologics Association favors leukocyte-poor PRP, other groups highlight inconclusive meta-analyses and consider both leukocyte-poor and rich-PRP to be valid options for KOA management. Finally, regarding activation, the different consensus groups state that PRP classification systems must be based on the activation method. About 60% of the participants agreed that PRP activation is clinically necessary prior to its use [130–133]. On the basis of the current evidence, activated PRP appears to be more promising than nonactivated PRP for mild-to-moderate KOA. However, no consistent benefit of higher platelet or leukocyte doses has been demonstrated. Routine standardization and reporting of PRP composition are strongly recommended.

### Limitations

Despite its valuable findings, this study has certain methodological limitations. Firstly, there are the limitations inherent to each RCT, such as the heterogeneity of the experimental designs, population demographics, severity of KOA, PRP manufacturing and application protocols, administration regimens, measured outcomes, and follow-up points. Secondly, while some clinical outcome measures target similar parameters, the results obtained in the present review varied according to the reported scale. In this sense, differences in the number of items, their specificity, and their variability could affect statistical significance [124, 135–137]. For instance, KOOS domains comprise more detailed questions regarding specific activities, enhancing their sensitivity to subtle changes [135]. In contrast, WOMAC includes fewer, broader items, which could mask improvements [136]. Additionally, the alignment of scales and reference values—for instance, KOOS scores range from 0 to 100, with higher values indicating better outcomes, whereas WOMAC scale are scored in the opposite direction, with higher scores reflecting greater pain—may influence outcome measures. Ceiling or floor effects may limit the ability to detect changes and influence statistical significance. These methodological limitations may partly explain the differences in statistical significance observed between comparable outcome scales that assess similar variables of pain and function in KOA. The mean age of the analyzed population could also influence the disparity in results [124, 135–137].

Thirdly, the lack of standardized reporting and the scarcity of detailed methodological descriptions required the use of indirect estimates based on manufacturer specifications, published hematological data, and baseline whole-blood reference ranges. The classification strategy was deliberately designed to maximize simplicity while minimizing misclassification, employing the Mishra system with broad categories that remain sufficiently informative for comparative analyses. While these practices introduce some uncertainty, they enable the synthesis of a large and clinically informative evidence base, particularly important in the context of network meta-analysis where direct evidence is limited. The observed associations regarding the clinical effect of exogenous activation and PRP composition should therefore be interpreted in the context of the available evidence rather than as definitive causal effects. Furthermore, misclassification bias may arise in studies where PRP activation status is not explicitly reported, as the absence of information regarding exogenous activation was interpreted as evidence of nonactivation. Future studies should provide standardized, directly measured PRP composition to allow more robust causal inference.

Fourthly, a further limitation of the present network meta-analysis is the heterogeneity of comparators included across the selected studies. The networks incorporate a wide range of interventions—including placebo, HA, COR, MFAT, BMC, NSAIDs, structured exercise programs, or arthroscopic procedures—which differ substantially in their mechanisms of action, expected duration of effect, and typical clinical outcomes. Such diversity may introduce additional variability into effect estimates, potentially reducing their precision and interpretability. Differences in pharmacodynamics, biological activity, and procedural invasiveness among these comparators may influence both short- and long-term outcomes in ways that cannot be fully adjusted for in indirect comparisons. Although network meta-analysis allows the integration of both direct and indirect evidence and can provide estimates even in the absence of head-to-head trials, the assumption of transitivity requires that differences across studies do not systematically bias relative effect estimates. In the present network, the wide range of comparator mechanisms represents a potential source of inconsistency and imprecision, which must be considered when interpreting results.

## Conclusions

Although PRP is effective for KOA, our findings do not support the “more is better” approach regarding platelet and leukocyte enrichment. However, activation of PRP may confer benefits. Future RCTs directly comparing well-characterized PRP formulations are needed to identify the PRP properties associated with optimal clinical outcomes.

## Supplementary Information

Additional file1 (PDF 10089 KB)

## Data Availability

The datasets generated during and/or analyzed during the current study are available from the corresponding author on reasonable request.

## Abbreviations

ARTHRO: Arthroscopy
BMC: Bone marrow concentrate
CI: Confidence interval
COR: Corticosteroids
EQ-VAS: EuroQol visual analog scale
HA: Hyaluronic acid
IA: Intra-articular
ICRS: International Cartilage Repair Society
IKDC: International Knee Documentation Committee Score
IQR: Interquartile range
KC: Knee circumference
KL: Kellgren and Lawrence
KOA: Knee osteoarthritis
KOOS: Knee Injury and Osteoarthritis Outcome Score
MCID: Minimal clinically important difference
MD: Mean differences
MFAT: Microfragmented adipose tissue
MRI: Magnetic resonance imaging
NA: Not applicable
NR: Not reported
NSAID: Nonsteroidal anti-inflammatory drugs
OA: Osteoarthritis
OARSI: Osteoarthritis Research Society International
OMERATC: Outcome measures in rheumatology
OZO: Ozone therapy
na: Non-activated
PRP: Platelet-rich plasma
RCT: Randomized controlled trial
ROM: Range of motion
SEP: Structured exercise program
SUCRA: Surface under the cumulative ranking curve
VAS: Visual analog scale
WBC: White blood cells
WOMAC: Western Ontario and McMaster Universities Arthritis Index Score
