# Supplementary material for: Influence of platelet-rich plasma composition on pain and functional performance in knee osteoarthritis: a systematic review and network meta-analysis
Source: Knee Surg Relat Res. 2026 Apr 21;38:17. doi: 10.1186/s43019-026-00318-4 (PMC13101336; doi:10.1186/s43019-026-00318-4)
Supplement: Supplementary file 1 — Additional file1 (PDF 10089 KB) [file 43019_2026_318_MOESM1_ESM.pdf]

Knee Surgery & Related Research

Title: Influence of Platelet-rich Plasma Composition on Pain and Functional Performance in Knee Osteoarthritis: A Systematic Review and network Meta-Analysis

Running title: PRP composition in Knee Osteoarthritis

Supplementary Material Index

Search Strategy..1
PRP Characterization...3
Table S1. Characterization of whole blood and platelet-rich plasma of the studies included in the systematic review..4
Table S2. PRP dosing and administration regimen..8
Table S3. Classification of platelet-rich plasma applied in the studies included in the systematic review according to Mishra's classification system...................................12
Table S4. Data sources for PRP characterization...14
Effect of PRP activation..16
WOMAC Pain..17
Figure S1. Contour enhanced funnel plot for trials of the effect of PRP stratified by platelet activation status (activated PRP = aPRP, and non-activated PRP = naPRP) vs different non-surgical control treatments (ACE: Acetaminophen, BMC: Bone Marrow Concentrate, COR: Corticosteroids, HA: Hyaluronic Acid, NSAID: Non-steroidal Anti-Inflammatory Drugs, OZO: Ozone therapy, and PBO: Placebo) on WOMAC Pain at the following time points: A) 6 months, and B) 12 months. The vertical line represents the pooled effect estimate. Contour lines indicate regions of statistical significance (p<0.01, p<0.05, and p<0.10). Asymmetry in the distribution of studies may suggest potential publication bias or small-study effects...18
Figure S2. Network plot representing direct comparisons among interventions included in the network meta-analysis for WOMAC Pain at the following time points: A) 6 months, and B) 12 months. PRP treatments were stratified by platelet activation status (activated PRP = aPRP, and non-activated PRP = naPRP) vs different non-surgical control treatments (ACE: Acetaminophen, BMC: Bone Marrow Concentrate, COR: Corticosteroids, HA: Hyaluronic Acid, NSAID: Non-steroidal Anti-Inflammatory Drugs, OZO: Ozone therapy, and PBO: Placebo). Node size is proportional to the total number of participants receiving each intervention, and edge thickness reflects the number of studies contributing to each direct comparison...19
Figure S3. Node-split forest plot representing inconsistency assessment in the network meta-analysis of the effect of PRP stratified by platelet activation status (activated PRP =aPRP, and non-activated PRP = naPRP) vs different non-surgical control treatments (ACE: Acetaminophen, BMC: Bone Marrow Concentrate, COR: Corticosteroids, HA: Hyaluronic Acid, NSAID: Non-steroidal Anti-Inflammatory Drugs, OZO: Ozone therapy, and PBO: Placebo) on WOMAC Pain at the following time points: A) 6 months, and B) 12 months. Each treatment comparison includes both direct and indirect estimates for mean difference (MD) with corresponding 95% confidence intervals...20
Figure S4. Direct evidence plot representing direct evidence proportion for each assessment corresponding to the network meta-analysis of the effect of PRP stratified by platelet activation status (activated PRP = aPRP, and non-activated PRP = naPRP) vs different non-surgical control treatments (ACE: Acetaminophen, BMC: Bone Marrow Concentrate, COR: Corticosteroids, HA: Hyaluronic Acid, NSAID: Non-steroidal Anti-Inflammatory Drugs, OZO: Ozone therapy, and PBO: Placebo) on WOMAC Pain at the following time points: A) 6 months, and B) 12 months. Key geometry metrics depicted include minimal path length, representing the shortest distance between nodes, and mean path length, reflecting the

shortest parallel connections between nodes, and mean path length, quantifying the average shortest path across all pairs of interventions within the network........21
WOMAC Stiffness........22
Figure S5. Contour enhanced funnel plot for trials of the effect of PRP stratified by platelet activation status (activated PRP = aPRP, and non-activated PRP = naPRP) vs different non-surgical control treatments (ACE: Acetaminophen, BMC: Bone Marrow Concentrate, COR: Corticosteroids, HA: Hyaluronic Acid, NSAID: Non-steroidal Anti-Inflammatory Drugs, OZO: Ozone therapy, and PBO: Placebo) on WOMAC Stiffness at the following time points: A) 6 months, and B) 12 months. The vertical line represents the pooled effect estimate. Contour lines indicate regions of statistical significance (p<0.01, p<0.05, and p<0.10). Asymmetry in the distribution of studies may suggest potential publication bias or small-study effects........23
Figure S6. Network plot representing direct comparisons among interventions included in the network meta-analysis for WOMAC Stiffness at the following time points: A) 6 months, and B) 12 months. PRP treatments were stratified by platelet activation status (activated PRP = aPRP, and non-activated PRP = naPRP) vs different non-surgical control treatments (ACE: Acetaminophen, BMC: Bone Marrow Concentrate, COR: Corticosteroids, HA: Hyaluronic Acid, NSAID: Non-steroidal Anti-Inflammatory Drugs, OZO: Ozone therapy, and PBO: Placebo). Node size is proportional to the total number of participants receiving each intervention, and edge thickness reflects the number of studies contributing to each direct comparison........24
Figure S7. Node-split forest plot representing inconsistency assessment in the network meta-analysis of the effect of PRP stratified by platelet activation status (activated PRP =aPRP, and non-activated PRP = naPRP) vs different non-surgical control treatments (ACE: Acetaminophen, BMC: Bone Marrow Concentrate, COR: Corticosteroids, HA: Hyaluronic Acid, NSAID: Non-steroidal Anti-Inflammatory Drugs, OZO: Ozone therapy, and PBO: Placebo) on WOMAC Stiffness at the following time points: A) 6 months, and B) 12 months. Each treatment comparison includes both direct and indirect estimates for mean difference (MD) with corresponding 95% confidence intervals........25
Figure S8. Direct evidence plot representing direct evidence proportion for each assessment corresponding to the network meta-analysis of the effect of PRP stratified by platelet activation status (activated PRP = aPRP, and non-activated PRP = naPRP) vs different non-surgical control treatments (ACE: Acetaminophen, BMC: Bone Marrow Concentrate, COR: Corticosteroids, HA: Hyaluronic Acid, NSAID: Non-steroidal Anti-Inflammatory Drugs, OZO: Ozone therapy, and PBO: Placebo) on WOMAC Stiffness at the following time points: A) 6 months, and B) 12 months. Key geometry metrics depicted include minimal path length, representing the shortest distance between nodes, and mean path length, reflecting the shortest parallel connections between nodes, and mean path length, quantifying the average shortest path across all pairs of interventions within the network........26
WOMAC Physical Function........27
Figure S9. Contour enhanced funnel plot for trials of the effect of PRP stratified by platelet activation status (activated PRP = aPRP, and non-activated PRP = naPRP) vs different non-surgical control treatments (ACE: Acetaminophen, BMC: Bone Marrow Concentrate, COR: Corticosteroids, HA: Hyaluronic Acid, NSAID: Non-steroidal Anti-Inflammatory Drugs, OZO: Ozone therapy, and PBO: Placebo) on

WOMAC Physical Function at the following time points: A) 6 months, and B) 12 months. The vertical line represents the pooled effect estimate. Contour lines indicate regions of statistical significance (p<0.01, p<0.05, and p<0.10). Asymmetry in the distribution of studies may suggest potential publication bias or small-study effects..28

Figure S10. Network plot representing direct comparisons among interventions included in the network meta-analysis for WOMAC Physical Function at the following time points: A) 6 months, and B) 12 months. PRP treatments were stratified by platelet activation status (activated PRP = aPRP, and non-activated PRP = naPRP) vs different non-surgical control treatments (ACE: Acetaminophen, BMC: Bone Marrow Concentrate, COR: Corticosteroids, HA: Hyaluronic Acid, NSAID: Non-steroidal Anti-Inflammatory Drugs, OZO: Ozone therapy, and PBO: Placebo). Node size is proportional to the total number of participants receiving each intervention, and edge thickness reflects the number of studies contributing to each direct comparison..29

Figure S11. Node-split forest plot representing inconsistency assessment in the network meta-analysis of the effect of PRP stratified by platelet activation status (activated PRP =aPRP, and non-activated PRP = naPRP) vs different non-surgical control treatments (ACE: Acetaminophen, BMC: Bone Marrow Concentrate, COR: Corticosteroids, HA: Hyaluronic Acid, NSAID: Non-steroidal Anti-Inflammatory Drugs, OZO: Ozone therapy, and PBO: Placebo) on WOMAC Physical Function at the following time points: A) 6 months, and B) 12 months. Each treatment comparison includes both direct and indirect estimates for mean difference (MD) with corresponding 95%confidence intervals...30

Figure S12. Direct evidence plot representing direct evidence proportion for each assessment corresponding to the network meta-analysis of the effect of PRP stratified by platelet activation status (activated PRP = aPRP, and non-activated PRP = naPRP) vs different non-surgical control treatments (ACE: Acetaminophen, BMC: Bone Marrow Concentrate, COR: Corticosteroids, HA: Hyaluronic Acid, NSAID: Non-steroidal Anti-Inflammatory Drugs, OZO: Ozone therapy, and PBO: Placebo) on WOMAC Physical Function at the following time points: A) 6 months, and B) 12 months. Key geometry metrics depicted include minimal path length, representing the shortest distance between nodes, and mean path length, reflecting the shortest parallel connections between nodes, and mean path length, quantifying the average shortest path across all pairs of interventions within the network...31

WOMAC Total...32

Figure S13. Contour enhanced funnel plot for trials of the effect of PRP stratified by platelet activation status (activated PRP = aPRP, and non-activated PRP = naPRP) vs different non-surgical control treatments (ACE: Acetaminophen, BMC: Bone Marrow Concentrate, COR: Corticosteroids, HA: Hyaluronic Acid, NSAID: Non-steroidal Anti-Inflammatory Drugs, OZO: Ozone therapy, and PBO: Placebo) on WOMAC Total at the following time points: A) 6 months, and B) 12 months. The vertical line represents the pooled effect estimate. Contour lines indicate regions of statistical significance (p<0.01, p<0.05, and p<0.10). Asymmetry in the distribution of studies may suggest potential publication bias or small-study effects...33

Figure S14. Network plot representing direct comparisons among interventions included in the network meta-analysis for WOMAC Total at the following time points: A) 6 months, and B) 12 months. PRP treatments were stratified by platelet activation status (activated PRP = aPRP, and non-activated PRP = naPRP) vs different non-surgical control treatments (ACE: Acetaminophen, BMC: Bone

Marrow Concentrate, COR: Corticosteroids, HA: Hyaluronic Acid, NSAID: Non-steroidal Anti-Inflammatory Drugs, OZO: Ozone therapy, and PBO: Placebo). Node size is proportional to the total number of participants receiving each intervention, and edge thickness reflects the number of studies contributing to each direct comparison..34

Figure S15. Node-split forest plot representing inconsistency assessment in the network meta-analysis of the effect of PRP stratified by platelet activation status (activated PRP =aPRP, and non-activated PRP = naPRP) vs different non-surgical control treatments (ACE: Acetaminophen, BMC: Bone Marrow Concentrate, COR: Corticosteroids, HA: Hyaluronic Acid, NSAID: Non-steroidal Anti-Inflammatory Drugs, OZO: Ozone therapy, and PBO: Placebo) on WOMAC Total at the following time points: A) 6 months, and B) 12 months. Each treatment comparison includes both direct and indirect estimates for mean difference (MD) with corresponding 95% confidence intervals..35

Figure S16. Direct evidence plot representing direct evidence proportion for each assessment corresponding to the network meta-analysis of the effect of PRP stratified by platelet activation status (activated PRP = aPRP, and non-activated PRP = naPRP) vs different non-surgical control treatments (ACE: Acetaminophen, BMC: Bone Marrow Concentrate, COR: Corticosteroids, HA: Hyaluronic Acid, NSAID: Non-steroidal Anti-Inflammatory Drugs, OZO: Ozone therapy, and PBO: Placebo) on WOMAC Total at the following time points: A) 6 months, and B) 12 months. Key geometry metrics depicted include minimal path length, representing the shortest distance between nodes, and mean path length, reflecting the shortest parallel connections between nodes, and mean path length, quantifying the average shortest path across all pairs of interventions within the network...36

KOOS Pain...37

Figure S17. Contour enhanced funnel plot for trials of the effect of PRP stratified by platelet activation status (activated PRP = aPRP, and non-activated PRP = naPRP) vs different non-surgical control treatments (COR: Corticosteroids, HA: Hyaluronic Acid, MFAT: Microfragmented Adipose Tissue, and PBO: Placebo) on KOOS Pain at the following time points: A) 6 months, and B) 12 months. The vertical line represents the pooled effect estimate. Contour lines indicate regions of statistical significance (p<0.01, p<0.05, and p<0.10). Asymmetry in the distribution of studies may suggest potential publication bias or small-study effects..38

Figure S18. Network plot representing direct comparisons among interventions included in the network meta-analysis for KOOS Pain at the following time points: A) 6 months, and B) 12 months. PRP treatments were stratified by platelet activation status (activated PRP = aPRP, and non-activated PRP = naPRP) vs different non-surgical control treatments (COR: Corticosteroids, HA: Hyaluronic Acid, MFAT: Microfragmented Adipose Tissue, and PBO: Placebo). Node size is proportional to the total number of participants receiving each intervention, and edge thickness reflects the number of studies contributing to each direct comparison..39

Figure S19. Node-split forest plot representing inconsistency assessment in the network meta-analysis of the effect of PRP stratified by platelet activation status (activated PRP =aPRP, and non-activated PRP = naPRP) vs different non-surgical control treatments (COR: Corticosteroids, HA: Hyaluronic Acid, MFAT: Microfragmented Adipose Tissue, and PBO: Placebo) on KOOS Pain at the following time points: A) 6 months, and B) 12 months. Each treatment comparison includes both direct and indirect estimates for mean difference (MD) with

corresponding 95%confidence intervals..40
Figure S20. Direct evidence plot representing direct evidence proportion for each assessment corresponding to the network meta-analysis of the effect of PRP stratified by platelet activation status (activated PRP = aPRP, and non-activated PRP = naPRP) vs different non-surgical control treatments (COR: Corticosteroids, HA: Hyaluronic Acid, MFAT: Microfragmented Adipose Tissue, and PBO: Placebo) on KOOS Pain at the following time points: A) 6 months, and B) 12 months. Key geometry metrics depicted include minimal path length, representing the shortest distance between nodes, and mean path length, reflecting the shortest parallel connections between nodes, and mean path length, quantifying the average shortest path across all pairs of interventions within the network..41
KOOS Activities of Daily Living..42
Figure S21. Contour enhanced funnel plot for trials of the effect of PRP stratified by platelet activation status (activated PRP = aPRP, and non-activated PRP = naPRP) vs different non-surgical control treatments (COR: Corticosteroids, HA: Hyaluronic Acid, MFAT: Microfragmented Adipose Tissue, and PBO: Placebo) on KOOS Activities of Daily Living at the following time points: A) 6 months, and B) 12 months. The vertical line represents the pooled effect estimate. Contour lines indicate regions of statistical significance (p<0.01, p<0.05, and p<0.10). Asymmetry in the distribution of studies may suggest potential publication bias or small-study effects..43
Figure S22. Network plot representing direct comparisons among interventions included in the network meta-analysis for KOOS Activities of Daily Living at the following time points: A) 6 months, and B) 12 months. PRP treatments were stratified by platelet activation status (activated PRP = aPRP, and non-activated PRP = naPRP) vs different non-surgical control treatments (COR: Corticosteroids, HA: Hyaluronic Acid, MFAT: Microfragmented Adipose Tissue, and PBO: Placebo). Node size is proportional to the total number of participants receiving each intervention, and edge thickness reflects the number of studies contributing to each direct comparison..44
Figure S23. Node-split forest plot representing inconsistency assessment in the network meta-analysis of the effect of PRP stratified by platelet activation status (activated PRP =aPRP, and non-activated PRP = naPRP) vs different non-surgical control treatments (COR: Corticosteroids, HA: Hyaluronic Acid, MFAT: Microfragmented Adipose Tissue, and PBO: Placebo) on KOOS Activities of Daily Living at the following time points: A) 6 months, and B) 12 months. Each treatment comparison includes both direct and indirect estimates for mean difference (MD) with corresponding 95%confidence intervals..45
Figure S24. Direct evidence plot representing direct evidence proportion for each assessment corresponding to the network meta-analysis of the effect of PRP stratified by platelet activation status (activated PRP = aPRP, and non-activated PRP = naPRP) vs different non-surgical control treatments (COR: Corticosteroids, HA: Hyaluronic Acid, MFAT: Microfragmented Adipose Tissue, and PBO: Placebo) on KOOS Activities of Daily Living at the following time points: A) 6 months, and B) 12 months. Key geometry metrics depicted include minimal path length, representing the shortest distance between nodes, and mean path length, reflecting the shortest parallel connections between nodes, and mean path length, quantifying the average shortest path across all pairs of interventions within the network..46
KOOS Sports and Recreation Function.......47

Figure S25. Contour enhanced funnel plot for trials of the effect of PRP stratified by platelet activation status (activated PRP = aPRP, and non-activated PRP = naPRP) vs different non-surgical control treatments (COR: Corticosteroids, HA: Hyaluronic Acid, MFAT: Microfragmented Adipose Tissue, and PBO: Placebo) on KOOS Sports and Recreation Function at the following time points: A) 6 months, and B) 12 months. The vertical line represents the pooled effect estimate. Contour lines indicate regions of statistical significance (p<0.01, p<0.05, and p<0.10). Asymmetry in the distribution of studies may suggest potential publication bias or small-study effects..48
Figure S26. Network plot representing direct comparisons among interventions included in the network meta-analysis for KOOS Sports and Recreation Function at the following time points: A) 6 months, and B) 12 months. PRP treatments were stratified by platelet activation status (activated PRP = aPRP, and non-activated PRP = naPRP) vs different non-surgical control treatments (COR: Corticosteroids, HA: Hyaluronic Acid, MFAT: Microfragmented Adipose Tissue, and PBO: Placebo). Node size is proportional to the total number of participants receiving each intervention, and edge thickness reflects the number of studies contributing to each direct comparison...49
Figure S27. Node-split forest plot representing inconsistency assessment in the network meta-analysis of the effect of PRP stratified by platelet activation status (activated PRP =aPRP, and non-activated PRP = naPRP) vs different non-surgical control treatments (ACE: Acetaminophen, BMC: Bone Marrow Concentrate, COR: Corticosteroids, HA: Hyaluronic Acid, NSAID: Non-steroidal Anti-Inflammatory Drugs, and OZO: Ozone therapy) on KOOS Sports and Recreation Function at the following time points: A) 6 months, and B) 12 months. Each treatment comparison includes both direct and indirect estimates for mean difference (MD) with corresponding 95% confidence intervals...50
Figure S28. Direct evidence plot representing direct evidence proportion for each assessment corresponding to the network meta-analysis of the effect of PRP stratified by platelet activation status (activated PRP = aPRP, and non-activated PRP = naPRP) vs different non-surgical control treatments (COR: Corticosteroids, HA: Hyaluronic Acid, MFAT: Microfragmented Adipose Tissue, and PBO: Placebo) on KOOS Sports and Recreation Function at the following time points: A) 6 months, and B) 12 months. Key geometry metrics depicted include minimal path length, representing the shortest distance between nodes, and mean path length, reflecting the shortest parallel connections between nodes, and mean path length, quantifying the average shortest path across all pairs of interventions within the network...51
KOOS Knee-Related Quality of Life...52
Figure S29. Contour enhanced funnel plot for trials of the effect of PRP stratified by platelet activation status (activated PRP = aPRP, and non-activated PRP = naPRP) vs different non-surgical control treatments (COR: Corticosteroids, HA: Hyaluronic Acid, MFAT: Microfragmented Adipose Tissue, and PBO: Placebo) on KOOS Knee-Related Quality of Life at the following time points: A) 6 months, and B) 12 months. The vertical line represents the pooled effect estimate. Contour lines indicate regions of statistical significance (p<0.01, p<0.05, and p<0.10). Asymmetry in the distribution of studies may suggest potential publication bias or small-study effects..53
Figure S30. Network plot representing direct comparisons among interventions included in the network meta-analysis for KOOS Knee-Related Quality of Life at the following time points: A) 6 months, and B) 12 months. PRP treatments were stratified by platelet activation status (activated PRP = aPRP, and non-activated

PRP = naPRP) vs different non-surgical control treatments (COR: Corticosteroids, HA: Hyaluronic Acid, MFAT: Microfragmented Adipose Tissue, and PBO: Placebo). Node size is proportional to the total number of participants receiving each intervention, and edge thickness reflects the number of studies contributing to each direct comparison..54
Figure S31. Node-split forest plot representing inconsistency assessment in the network meta-analysis of the effect of PRP stratified by platelet activation status (activated PRP =aPRP, and non-activated PRP = naPRP) vs different non-surgical control treatments (COR: Corticosteroids, HA: Hyaluronic Acid, MFAT: Microfragmented Adipose Tissue, and PBO: Placebo) on KOOS Knee-Related Quality of Life at the following time points: A) 6 months, and B) 12 months. Each treatment comparison includes both direct and indirect estimates for mean difference (MD) with corresponding 95% confidence intervals..........................55
Figure S32. Direct evidence plot representing direct evidence proportion for each assessment corresponding to the network meta-analysis of the effect of PRP stratified by platelet activation status (activated PRP = aPRP, and non-activated PRP = naPRP) vs different non-surgical control treatments (COR: Corticosteroids, HA: Hyaluronic Acid, MFAT: Microfragmented Adipose Tissue, and PBO: Placebo) on KOOS Knee-Related Quality of Life at the following time points: A) 6 months, and B) 12 months. Key geometry metrics depicted include minimal path length, representing the shortest distance between nodes, and mean path length, reflecting the shortest parallel connections between nodes, and mean path length, quantifying the average shortest path across all pairs of interventions within the network...56
KOOS Symptoms...57
Figure S33. Contour enhanced funnel plot for trials of the effect of PRP stratified by platelet activation status (activated PRP = aPRP, and non-activated PRP = naPRP) vs different non-surgical control treatments (COR: Corticosteroids, HA: Hyaluronic Acid, MFAT: Microfragmented Adipose Tissue, and PBO: Placebo) on KOOS Symptoms at the following time points: A) 6 months, and B) 12 months. The vertical line represents the pooled effect estimate. Contour lines indicate regions of statistical significance (p<0.01, p<0.05, and p<0.10). Asymmetry in the distribution of studies may suggest potential publication bias or small-study effects...58
Figure S34. Network plot representing direct comparisons among interventions included in the network meta-analysis for KOOS Symptoms at the following time points: A) 6 months, and B) 12 months. PRP treatments were stratified by platelet activation status (activated PRP = aPRP, and non-activated PRP = naPRP) vs different non-surgical control treatments (COR: Corticosteroids, HA: Hyaluronic Acid, MFAT: Microfragmented Adipose Tissue, and PBO: Placebo). Node size is proportional to the total number of participants receiving each intervention, and edge thickness reflects the number of studies contributing to each direct comparison...59
Figure S35. Node-split forest plot representing inconsistency assessment in the network meta-analysis of the effect of PRP stratified by platelet activation status (activated PRP =aPRP, and non-activated PRP = naPRP) vs different non-surgical control treatments (COR: Corticosteroids, HA: Hyaluronic Acid, MFAT: Microfragmented Adipose Tissue, and PBO: Placebo) on KOOS Symptoms at the following time points: A) 6 months, and B) 12 months. Each treatment comparison includes both direct and indirect estimates for mean difference (MD) with corresponding 95% confidence intervals..60

Figure S36. Direct evidence plot representing direct evidence proportion for each assessment corresponding to the network meta-analysis of the effect of PRP stratified by platelet activation status (activated PRP = aPRP, and non-activated PRP = naPRP) vs different non-surgical control treatments (COR: Corticosteroids, HA: Hyaluronic Acid, MFAT: Microfragmented Adipose Tissue, and PBO: Placebo) on KOOS Symptoms at the following time points: A) 6 months, and B) 12 months. Key geometry metrics depicted include minimal path length, representing the shortest distance between nodes, and mean path length, reflecting the shortest parallel connections between nodes, and mean path length, quantifying the average shortest path across all pairs of interventions within the network..61

VAS...62

Figure S37. Contour enhanced funnel plot for trials of the effect of PRP stratified by platelet activation status (activated PRP = aPRP, and non-activated PRP = naPRP) vs different non-surgical control treatments (ACE: Acetaminophen, ARTHRO: Arthroscopy, BMC: Bone Marrow Concentrate, COR: Corticosteroids, HA: Hyaluronic Acid, MFAT: Microfragmented Adipose Tissue, NSAID: Non-steroidal Anti-Inflammatory Drugs, OZO: Ozone therapy, and PBO: Placebo) on VAS at the following time points: A) 6 months, and B) 12 months. The vertical line represents the pooled effect estimate. Contour lines indicate regions of statistical significance (p<0.01, p<0.05, and p<0.10). Asymmetry in the distribution of studies may suggest potential publication bias or small-study effects..63

Figure S38. Network plot representing direct comparisons among interventions included in the network meta-analysis for VAS at the following time points: A) 6 months, and B) 12 months. PRP treatments were stratified by platelet activation status (activated PRP = aPRP, and non-activated PRP = naPRP) vs different non-surgical control treatments (ACE: Acetaminophen, ARTHRO: Arthroscopy, BMC: Bone Marrow Concentrate, COR: Corticosteroids, HA: Hyaluronic Acid, MFAT: Microfragmented Adipose Tissue, NSAID: Non-steroidal Anti-Inflammatory Drugs, OZO: Ozone therapy, and PBO: Placebo). Node size is proportional to the total number of participants receiving each intervention, and edge thickness reflects the number of studies contributing to each direct comparison..64

Figure S39. Node-split forest plot representing inconsistency assessment in the network meta-analysis of the effect of PRP stratified by platelet activation status (activated PRP =aPRP, and non-activated PRP = naPRP) vs different non-surgical control treatments (ACE: Acetaminophen, ARTHRO: Arthroscopy, BMC: Bone Marrow Concentrate, COR: Corticosteroids, HA: Hyaluronic Acid, MFAT: Microfragmented Adipose Tissue, NSAID: Non-steroidal Anti-Inflammatory Drugs, OZO: Ozone therapy, and PBO: Placebo) on VAS at the following time points: A) 6 months, and B) 12 months. Each treatment comparison includes both direct and indirect estimates for mean difference (MD) with corresponding 95%confidence intervals..65

Figure S40. Direct evidence plot representing direct evidence proportion for each assessment corresponding to the network meta-analysis of the effect of PRP stratified by platelet activation status (activated PRP = aPRP, and non-activated PRP = naPRP) vs different non-surgical control treatments (ACE: Acetaminophen, ARTHRO: Arthroscopy, BMC: Bone Marrow Concentrate, COR: Corticosteroids, HA: Hyaluronic Acid, MFAT: Microfragmented Adipose Tissue, NSAID: Non-steroidal Anti-Inflammatory Drugs, OZO: Ozone therapy, and PBO: Placebo) on VAS at the following time points: A) 6 months, and B) 12 months. Key geometry

metrics depicted include minimal path length, representing the shortest distance between nodes, and mean path length, reflecting the shortest parallel connections between nodes, and mean path length, quantifying the average shortest path across all pairs of interventions within the network........66

IKDC........67

Figure S41. Contour enhanced funnel plot for trials of the effect of PRP stratified by platelet activation status (activated PRP = aPRP, and non-activated PRP = naPRP) vs different non-surgical control treatments (BMC: Bone Marrow Concentrate, COR: Corticosteroids, HA: Hyaluronic Acid, MFAT: Microfragmented Adipose Tissue, PBO: Placebo, and SEP: Structured Exercise Program) on IKDC at the following time points: A) 6 months, and B) 12 months. The vertical line represents the pooled effect estimate. Contour lines indicate regions of statistical significance (p<0.01, p<0.05, and p<0.10). Asymmetry in the distribution of studies may suggest potential publication bias or small-study effects........68

Figure S42. Network plot representing direct comparisons among interventions included in the network meta-analysis for IKDC at the following time points: A) 6 months, and B) 12 months. PRP treatments were stratified by platelet activation status (activated PRP = aPRP, and non-activated PRP = naPRP) vs different non-surgical control treatments (BMC: Bone Marrow Concentrate, COR: Corticosteroids, HA: Hyaluronic Acid, MFAT: Microfragmented Adipose Tissue, PBO: Placebo, and SEP: Structured Exercise Program). Node size is proportional to the total number of participants receiving each intervention, and edge thickness reflects the number of studies contributing to each direct comparison........69

Figure S43. Node-split forest plot representing inconsistency assessment in the network meta-analysis of the effect of PRP stratified by platelet activation status (activated PRP =aPRP, and non-activated PRP = naPRP) vs different non-surgical control treatments (BMC: Bone Marrow Concentrate, COR: Corticosteroids, HA: Hyaluronic Acid, MFAT: Microfragmented Adipose Tissue, PBO: Placebo, and SEP: Structured Exercise Program) on IKDC at the following time points: A) 6 months, and B) 12 months. Each treatment comparison includes both direct and indirect estimates for mean difference (MD) with corresponding 95%confidence intervals........70

Figure S44. Direct evidence plot representing direct evidence proportion for each assessment corresponding to the network meta-analysis of the effect of PRP stratified by platelet activation status (activated PRP = aPRP, and non-activated PRP = naPRP) vs different non-surgical control treatments (BMC: Bone Marrow Concentrate, COR: Corticosteroids, HA: Hyaluronic Acid, MFAT: Microfragmented Adipose Tissue, PBO: Placebo, and SEP: Structured Exercise Program) on IKDC at the following time points: A) 6 months, and B) 12 months. Key geometry metrics depicted include minimal path length, representing the shortest distance between nodes, and mean path length, reflecting the shortest parallel connections between nodes, and mean path length, quantifying the average shortest path across all pairs of interventions within the network........71

Lequesne Index........72

Figure S45. Contour enhanced funnel plot for trials of the effect of PRP stratified by platelet activation status (activated PRP = aPRP, and non-activated PRP = naPRP) vs different non-surgical control treatments (COR: Corticosteroids, and HA: Hyaluronic Acid) on Lequesne Index at the following time points: A) 6 months, and B) 12 months. The vertical line represents the pooled effect estimate. Contour

lines indicate regions of statistical significance (p<0.01, p<0.05, and p<0.10). Asymmetry in the distribution of studies may suggest potential publication bias or small-study effects....73
Figure S46. Network plot representing direct comparisons among interventions included in the network meta-analysis for Lequesne Index at the following time points: A) 6 months, and B) 12 months. PRP treatments were stratified by platelet activation status (activated PRP = aPRP, and non-activated PRP = naPRP) vs different non-surgical control treatments (COR: Corticosteroids, and HA: Hyaluronic Acid). Node size is proportional to the total number of participants receiving each intervention, and edge thickness reflects the number of studies contributing to each direct comparison....74
Figure S47. Node-split forest plot representing inconsistency assessment in the network meta-analysis of the effect of PRP stratified by platelet activation status (activated PRP =aPRP, and non-activated PRP = naPRP) vs different non-surgical control treatments (COR: Corticosteroids, and HA: Hyaluronic Acid) on Lequesne Index at the following time points: A) 6 months, and B) 12 months. Each treatment comparison includes both direct and indirect estimates for mean difference (MD) with corresponding 95%confidence intervals....75
Figure S48. Direct evidence plot representing direct evidence proportion for each assessment corresponding to the network meta-analysis of the effect of PRP stratified by platelet activation status (activated PRP = aPRP, and non-activated PRP = naPRP) vs different non-surgical control treatments (COR: Corticosteroids, and HA: Hyaluronic Acid) on Lequesne Index at the following time points: A) 6 months, and B) 12 months. Key geometry metrics depicted include minimal path length, representing the shortest distance between nodes, and mean path length, reflecting the shortest parallel connections between nodes, and mean path length, quantifying the average shortest path across all pairs of interventions within the network....76
Effect of PRP categories according to Mishra's classification system....77
WOMAC Pain....78
Figure S49. Contour enhanced funnel plot for trials of the effect of PRP categorized according to Mishra's classification system vs different non-surgical control treatments (ACE: Acetaminophen, BMC: Bone Marrow Concentrate, COR: Corticosteroids, HA: Hyaluronic Acid, NSAID: Non-steroidal Anti-Inflammatory Drugs, OZO: Ozone therapy, and PBO: Placebo) on WOMAC Pain at the following time points: A) 6 months, and B) 12 months. The vertical line represents the pooled effect estimate. Contour lines indicate regions of statistical significance (p<0.01, p<0.05, and p<0.10). Asymmetry in the distribution of studies may suggest potential publication bias or small-study effects....79
Figure S50. Network plot representing direct comparisons among interventions included in the network meta-analysis for WOMAC Pain at the following time points: A) 6 months, and B) 12 months. PRP treatments were categorized according to Mishra's classification system vs different non-surgical control treatments (ACE: Acetaminophen, BMC: Bone Marrow Concentrate, COR: Corticosteroids, HA: Hyaluronic Acid, NSAID: Non-steroidal Anti-Inflammatory Drugs, OZO: Ozone therapy, and PBO: Placebo). Node size is proportional to the total number of participants receiving each intervention, and edge thickness reflects the number of studies contributing to each direct comparison....80
Figure S51. Node-split forest plot representing inconsistency assessment in the network meta-analysis of the effect of PRP categorized according to Mishra's classification system vs different non-surgical control treatments (ACE:

Acetaminophen, BMC: Bone Marrow Concentrate, COR: Corticosteroids, HA: Hyaluronic Acid, NSAID: Non-steroidal Anti-Inflammatory Drugs, OZO: Ozone therapy, and PBO: Placebo) on WOMAC Pain at the following time points: A) 6 months, and B) 12 months. Each treatment comparison includes both direct and indirect estimates for mean difference (MD) with corresponding 95% confidence intervals..81
Figure S52. Direct evidence plot representing direct evidence proportion for each assessment corresponding to the network meta-analysis of the effect of PRP categorized according to Mishra's classification system vs different non-surgical control treatments (ACE: Acetaminophen, BMC: Bone Marrow Concentrate, COR: Corticosteroids, HA: Hyaluronic Acid, NSAID: Non-steroidal Anti-Inflammatory Drugs, OZO: Ozone therapy, and PBO: Placebo) on WOMAC Pain at the following time points: A) 6 months, and B) 12 months. Key geometry metrics depicted include minimal path length, representing the shortest distance between nodes, and mean path length, reflecting the shortest parallel connections between nodes, and mean path length, quantifying the average shortest path across all pairs of interventions within the network...82
WOMAC Stiffness...83
Figure S53. Contour enhanced funnel plot for trials of the effect of PRP categorized according to Mishra's classification system vs different non-surgical control treatments (ACE: Acetaminophen, BMC: Bone Marrow Concentrate, COR: Corticosteroids, HA: Hyaluronic Acid, NSAID: Non-steroidal Anti-Inflammatory Drugs, OZO: Ozone therapy, and PBO: Placebo) on WOMAC Stiffness at the following time points: A) 6 months, and B) 12 months. The vertical line represents the pooled effect estimate. Contour lines indicate regions of statistical significance (p<0.01, p<0.05, and p<0.10). Asymmetry in the distribution of studies may suggest potential publication bias or small-study effects..84
Figure S54. Network plot representing direct comparisons among interventions included in the network meta-analysis for WOMAC Stiffness at the following time points: A) 6 months, and B) 12 months. PRP treatments were categorized according to Mishra's classification system vs different non-surgical control treatments (ACE: Acetaminophen, BMC: Bone Marrow Concentrate, COR: Corticosteroids, HA: Hyaluronic Acid, NSAID: Non-steroidal Anti-Inflammatory Drugs, OZO: Ozone therapy, and PBO: Placebo). Node size is proportional to the total number of participants receiving each intervention, and edge thickness reflects the number of studies contributing to each direct comparison..85
Figure S55. Node-split forest plot representing inconsistency assessment in the network meta-analysis of the effect of PRP categorized according to Mishra's classification system vs different non-surgical control treatments (ACE: Acetaminophen, BMC: Bone Marrow Concentrate, COR: Corticosteroids, HA: Hyaluronic Acid, NSAID: Non-steroidal Anti-Inflammatory Drugs, OZO: Ozone therapy, and PBO: Placebo) on WOMAC Stiffness at the following time points: A) 6 months, and B) 12 months. Each treatment comparison includes both direct and indirect estimates for mean difference (MD) with corresponding 95% confidence intervals..86
Figure S56. Direct evidence plot representing direct evidence proportion for each assessment corresponding to the network meta-analysis of the effect of PRP categorized according to Mishra's classification system vs different non-surgical control treatments (ACE: Acetaminophen, BMC: Bone Marrow Concentrate, COR: Corticosteroids, HA: Hyaluronic Acid, NSAID: Non-steroidal Anti-Inflammatory

Drugs, OZO: Ozone therapy, and PBO: Placebo) on WOMAC Stiffness at the following time points: A) 6 months, and B) 12 months. Key geometry metrics depicted include minimal path length, representing the shortest distance between nodes, and mean path length, reflecting the shortest parallel connections between nodes, and mean path length, quantifying the average shortest path across all pairs of interventions within the network..87
WOMAC Physical function...88
Figure S57. Contour enhanced funnel plot for trials of the effect of PRP categorized according to Mishra's classification system vs different non-surgical control treatments (ACE: Acetaminophen, BMC: Bone Marrow Concentrate, COR: Corticosteroids, HA: Hyaluronic Acid, NSAID: Non-steroidal Anti-Inflammatory Drugs, OZO: Ozone therapy, and PBO: Placebo) on WOMAC Physical Function at the following time points: A) 6 months, and B) 12 months. The vertical line represents the pooled effect estimate. Contour lines indicate regions of statistical significance (p<0.01, p<0.05, and p<0.10). Asymmetry in the distribution of studies may suggest potential publication bias or small-study effects..89
Figure S58. Network plot representing direct comparisons among interventions included in the network meta-analysis for WOMAC Physical Function at the following time points: A) 6 months, and B) 12 months. PRP treatments were categorized according to Mishra's classification system vs different non-surgical control treatments (ACE: Acetaminophen, BMC: Bone Marrow Concentrate, COR: Corticosteroids, HA: Hyaluronic Acid, NSAID: Non-steroidal Anti-Inflammatory Drugs, OZO: Ozone therapy, and PBO: Placebo). Node size is proportional to the total number of participants receiving each intervention, and edge thickness reflects the number of studies contributing to each direct comparison..90
Figure S59. Node-split forest plot representing inconsistency assessment in the network meta-analysis of the effect of PRP categorized according to Mishra's classification system vs different non-surgical control treatments (ACE: Acetaminophen, BMC: Bone Marrow Concentrate, COR: Corticosteroids, HA: Hyaluronic Acid, NSAID: Non-steroidal Anti-Inflammatory Drugs, OZO: Ozone therapy, and PBO: Placebo) on WOMAC Physical Function at the following time points: A) 6 months, and B) 12 months. Each treatment comparison includes both direct and indirect estimates for mean difference (MD) with corresponding 95% confidence intervals...91
Figure S60. Direct evidence plot representing direct evidence proportion for each assessment corresponding to the network meta-analysis of the effect of PRP categorized according to Mishra's classification system vs different non-surgical control treatments (ACE: Acetaminophen, BMC: Bone Marrow Concentrate, COR: Corticosteroids, HA: Hyaluronic Acid, NSAID: Non-steroidal Anti-Inflammatory Drugs, OZO: Ozone therapy, and PBO: Placebo) on WOMAC Physical Function at the following time points: A) 6 months, and B) 12 months. Key geometry metrics depicted include minimal path length, representing the shortest distance between nodes, and mean path length, reflecting the shortest parallel connections between nodes, and mean path length, quantifying the average shortest path across all pairs of interventions within the network..92
WOMAC Total...93
Figure S61. Contour enhanced funnel plot for trials of the effect of PRP categorized according to Mishra's classification system vs different non-surgical control treatments (ACE: Acetaminophen, BMC: Bone Marrow Concentrate, COR: Corticosteroids, HA: Hyaluronic Acid, NSAID: Non-steroidal Anti-Inflammatory

Drugs, OZO: Ozone therapy, and PBO: Placebo) on WOMAC Total at the following time points: A) 6 months, and B) 12 months. The vertical line represents the pooled effect estimate. Contour lines indicate regions of statistical significance (p<0.01, p<0.05, and p<0.10). Asymmetry in the distribution of studies may suggest potential publication bias or small-study effects..94

Figure S62. Network plot representing direct comparisons among interventions included in the network meta-analysis for WOMAC Total at the following time points: A) 6 months, and B) 12 months. PRP treatments were categorized according to Mishra's classification system vs different non-surgical control treatments (ACE: Acetaminophen, BMC: Bone Marrow Concentrate, COR: Corticosteroids, HA: Hyaluronic Acid, NSAID: Non-steroidal Anti-Inflammatory Drugs, OZO: Ozone therapy, and PBO: Placebo). Node size is proportional to the total number of participants receiving each intervention, and edge thickness reflects the number of studies contributing to each direct comparison...95

Figure S63. Node-split forest plot representing inconsistency assessment in the network meta-analysis of the effect of PRP categorized according to Mishra's classification system vs different non-surgical control treatments (ACE: Acetaminophen, BMC: Bone Marrow Concentrate, COR: Corticosteroids, HA: Hyaluronic Acid, NSAID: Non-steroidal Anti-Inflammatory Drugs, OZO: Ozone therapy, and PBO: Placebo) on WOMAC Total at the following time points: A) 6 months, and B) 12 months. Each treatment comparison includes both direct and indirect estimates for mean difference (MD) with corresponding 95% confidence intervals...96

Figure S64. Direct evidence plot representing direct evidence proportion for each assessment corresponding to the network meta-analysis of the effect of PRP categorized according to Mishra's classification system vs different non-surgical control treatments (ACE: Acetaminophen, BMC: Bone Marrow Concentrate, COR: Corticosteroids, HA: Hyaluronic Acid, NSAID: Non-steroidal Anti-Inflammatory Drugs, OZO: Ozone therapy, and PBO: Placebo) on WOMAC Total at the following time points: A) 6 months, and B) 12 months. Key geometry metrics depicted include minimal path length, representing the shortest distance between nodes, and mean path length, reflecting the shortest parallel connections between nodes, and mean path length, quantifying the average shortest path across all pairs of interventions within the network..97

KOOS Pain...98

Figure S65. Contour enhanced funnel plot for trials of the effect of PRP categorized according to Mishra's classification system vs different non-surgical control treatments (COR: Corticosteroids, HA: Hyaluronic Acid, MFAT: Microfragmented Adipose Tissue, and PBO: Placebo) on KOOS Pain at the following time points: A) 6 months, and B) 12 months. The vertical line represents the pooled effect estimate. Contour lines indicate regions of statistical significance (p<0.01, p<0.05, and p<0.10). Asymmetry in the distribution of studies may suggest potential publication bias or small-study effects...99

Figure S66. Network plot representing direct comparisons among interventions included in the network meta-analysis for KOOS Pain at the following time points: A) 6 months, and B) 12 months. PRP treatments were categorized according to Mishra's classification system vs different non-surgical control treatments (COR: Corticosteroids, HA: Hyaluronic Acid, MFAT: Microfragmented Adipose Tissue, and PBO: Placebo). Node size is proportional to the total number of participants receiving each intervention, and edge thickness reflects the number of studies contributing to each direct comparison...100

Figure S67. Node-split forest plot representing inconsistency assessment in the network meta-analysis of the effect of PRP categorized according to Mishra's classification system vs different non-surgical control treatments (COR: Corticosteroids, HA: Hyaluronic Acid, MFAT: Microfragmented Adipose Tissue, and PBO: Placebo) on KOOS Pain at the following time points: A) 6 months, and B) 12 months. Each treatment comparison includes both direct and indirect estimates for mean difference (MD) with corresponding 95% confidence intervals..101
Figure S68. Direct evidence plot representing direct evidence proportion for each assessment corresponding to the network meta-analysis of the effect of PRP categorized according to Mishra's classification system vs different non-surgical control treatments (COR: Corticosteroids, HA: Hyaluronic Acid, MFAT: Microfragmented Adipose Tissue, and PBO: Placebo) on KOOS Pain at the following time points: A) 6 months, and B) 12 months. Key geometry metrics depicted include minimal path length, representing the shortest distance between nodes, and mean path length, reflecting the shortest parallel connections between nodes, and mean path length, quantifying the average shortest path across all pairs of interventions within the network..102
KOOS Activities of Daily Living...103
Figure S69. Contour enhanced funnel plot for trials of the effect of PRP categorized according to Mishra's classification system vs different non-surgical control treatments (COR: Corticosteroids, HA: Hyaluronic Acid, MFAT: Microfragmented Adipose Tissue, and PBO: Placebo) on KOOS Activities of Daily Living at the following time points: A) 6 months, and B) 12 months. The vertical line represents the pooled effect estimate. Contour lines indicate regions of statistical significance (p<0.01, p<0.05, and p<0.10). Asymmetry in the distribution of studies may suggest potential publication bias or small-study effects...104
Figure S70. Network plot representing direct comparisons among interventions included in the network meta-analysis for KOOS Activities of Daily Living at the following time points: A) 6 months, and B) 12 months. PRP treatments were categorized according to Mishra's classification system vs different non-surgical control treatments (COR: Corticosteroids, HA: Hyaluronic Acid, MFAT: Microfragmented Adipose Tissue, and PBO: Placebo). Node size is proportional to the total number of participants receiving each intervention, and edge thickness reflects the number of studies contributing to each direct comparison............105
Figure S71. Node-split forest plot representing inconsistency assessment in the network meta-analysis of the effect of PRP categorized according to Mishra's classification system vs different non-surgical control treatments (COR: Corticosteroids, HA: Hyaluronic Acid, MFAT: Microfragmented Adipose Tissue, and PBO: Placebo) on KOOS Activities of Daily Living at the following time points: A) 6 months, and B) 12 months. Each treatment comparison includes both direct and indirect estimates for mean difference (MD) with corresponding 95% confidence intervals..106
Figure S72. Direct evidence plot representing direct evidence proportion for each assessment corresponding to the network meta-analysis of the effect of PRP categorized according to Mishra's classification system vs different non-surgical control treatments (COR: Corticosteroids, HA: Hyaluronic Acid, MFAT: Microfragmented Adipose Tissue, and PBO: Placebo) on KOOS Activities of Daily Living at the following time points: A) 6 months, and B) 12 months. Key geometry metrics depicted include minimal path length, representing the shortest distance between nodes, and mean path length, reflecting the shortest parallel

connections between nodes, and mean path length, quantifying the average shortest path across all pairs of interventions within the network......................107
KOOS Sport and Recreation Function..108
Figure S73. Contour enhanced funnel plot for trials of the effect of PRP categorized according to Mishra's classification system vs different non-surgical control treatments (COR: Corticosteroids, HA: Hyaluronic Acid, MFAT: Microfragmented Adipose Tissue, and PBO: Placebo) on KOOS Sport and Recreation Function at the following time points: A) 6 months, and B) 12 months. The vertical line represents the pooled effect estimate. Contour lines indicate regions of statistical significance (p<0.01, p<0.05, and p<0.10). Asymmetry in the distribution of studies may suggest potential publication bias or small-study effects...109
Figure S74. Network plot representing direct comparisons among interventions included in the network meta-analysis for KOOS Sport and Recreation Function at the following time points: A) 6 months, and B) 12 months. PRP treatments were categorized according to Mishra's classification system vs different non-surgical control treatments (COR: Corticosteroids, HA: Hyaluronic Acid, MFAT: Microfragmented Adipose Tissue, and PBO: Placebo). Node size is proportional to the total number of participants receiving each intervention, and edge thickness reflects the number of studies contributing to each direct comparison...110
Figure S75. Node-split forest plot representing inconsistency assessment in the network meta-analysis of the effect of PRP categorized according to Mishra's classification system vs different non-surgical control treatments (COR: Corticosteroids, HA: Hyaluronic Acid, MFAT: Microfragmented Adipose Tissue, and PBO: Placebo) on KOOS Sport and Recreation Function at the following time points: A) 6 months, and B) 12 months. Each treatment comparison includes both direct and indirect estimates for mean difference (MD) with corresponding 95% confidence intervals...111
Figure S76. Direct evidence plot representing direct evidence proportion for each assessment corresponding to the network meta-analysis of the effect of PRP categorized according to Mishra's classification system vs different non-surgical control treatments (COR: Corticosteroids, HA: Hyaluronic Acid, MFAT: Microfragmented Adipose Tissue, and PBO: Placebo) on KOOS Sport and Recreation Function at the following time points: A) 6 months, and B) 12 months. Key geometry metrics depicted include minimal path length, representing the shortest distance between nodes, and mean path length, reflecting the shortest parallel connections between nodes, and mean path length, quantifying the average shortest path across all pairs of interventions within the network...112
KOOS Knee-Related Quality of Life...113
Figure S77. Contour enhanced funnel plot for trials of the effect of PRP categorized according to Mishra's classification system vs different non-surgical control treatments (COR: Corticosteroids, HA: Hyaluronic Acid, MFAT: Microfragmented Adipose Tissue, and PBO: Placebo) on KOOS Knee-Related Quality of Life at the following time points: A) 6 months, and B) 12 months. The vertical line represents the pooled effect estimate. Contour lines indicate regions of statistical significance (p<0.01, p<0.05, and p<0.10). Asymmetry in the distribution of studies may suggest potential publication bias or small-study effects...114
Figure S78. Network plot representing direct comparisons among interventions included in the network meta-analysis for KOOS Knee-Related Quality of Life at the

following time points: A) 6 months, and B) 12 months. PRP treatments were categorized according to Mishra's classification system vs different non-surgical control treatments (COR: Corticosteroids, HA: Hyaluronic Acid, MFAT: Microfragmented Adipose Tissue, and PBO: Placebo). Node size is proportional to the total number of participants receiving each intervention, and edge thickness reflects the number of studies contributing to each direct comparison......115

Figure S79. Node-split forest plot representing inconsistency assessment in the network meta-analysis of the effect of PRP categorized according to Mishra's classification system vs different non-surgical control treatments (COR: Corticosteroids, HA: Hyaluronic Acid, MFAT: Microfragmented Adipose Tissue, and PBO: Placebo) on KOOS Knee-Related Quality of Life at the following time points: A) 6 months, and B) 12 months. Each treatment comparison includes both direct and indirect estimates for mean difference (MD) with corresponding 95% confidence intervals......116

Figure S80. Direct evidence plot representing direct evidence proportion for each assessment corresponding to the network meta-analysis of the effect of PRP categorized according to Mishra's classification system vs different non-surgical control treatments (COR: Corticosteroids, HA: Hyaluronic Acid, MFAT: Microfragmented Adipose Tissue, and PBO: Placebo) on KOOS Knee-Related Quality of Life at the following time points: A) 6 months, and B) 12 months. Key geometry metrics depicted include minimal path length, representing the shortest distance between nodes, and mean path length, reflecting the shortest parallel connections between nodes, and mean path length, quantifying the average shortest path across all pairs of interventions within the network......117

KOOS Symptoms......118

Figure S81. Contour enhanced funnel plot for trials of the effect of PRP categorized according to Mishra's classification system vs different non-surgical control treatments (COR: Corticosteroids, HA: Hyaluronic Acid, MFAT: Microfragmented Adipose Tissue, and PBO: Placebo) on KOOS Symptoms at the following time points: A) 6 months, and B) 12 months. The vertical line represents the pooled effect estimate. Contour lines indicate regions of statistical significance (p<0.01, p<0.05, and p<0.10). Asymmetry in the distribution of studies may suggest potential publication bias or small-study effects......119

Figure S82. Network plot representing direct comparisons among interventions included in the network meta-analysis for KOOS Symptoms at the following time points: A) 6 months, and B) 12 months. PRP treatments were categorized according to Mishra's classification system vs different non-surgical control treatments (COR: Corticosteroids, HA: Hyaluronic Acid, MFAT: Microfragmented Adipose Tissue, and PBO: Placebo). Node size is proportional to the total number of participants receiving each intervention, and edge thickness reflects the number of studies contributing to each direct comparison......120

Figure S83. Node-split forest plot representing inconsistency assessment in the network meta-analysis of the effect of PRP categorized according to Mishra's classification system vs different non-surgical control treatments (COR: Corticosteroids, HA: Hyaluronic Acid, MFAT: Microfragmented Adipose Tissue, and PBO: Placebo) on KOOS Symptoms at the following time points: A) 6 months, and B) 12 months. Each treatment comparison includes both direct and indirect estimates for mean difference (MD) with corresponding 95% confidence intervals......121

Figure S84. Direct evidence plot representing direct evidence proportion for each assessment corresponding to the network meta-analysis of the effect of PRP categorized according to Mishra's classification system vs different non-surgical control treatments (COR: Corticosteroids, HA: Hyaluronic Acid, MFAT: Microfragmented Adipose Tissue, and PBO: Placebo) on KOOS Symptoms at the following time points: A) 6 months, and B) 12 months. Key geometry metrics depicted include minimal path length, representing the shortest distance between nodes, and mean path length, reflecting the shortest parallel connections between nodes, and mean path length, quantifying the average shortest path across all pairs of interventions within the network...122

VAS...123

Figure S85. Contour enhanced funnel plot for trials of the effect of PRP categorized according to Mishra's classification system vs different non-surgical control treatments (ACE: Acetaminophen, ARTHRO: Arthroscopy, BMC: Bone Marrow Concentrate, COR: Corticosteroids, HA: Hyaluronic Acid, MFAT: Microfragmented Adipose Tissue, NSAID: Non-steroidal Anti-Inflammatory Drugs, OZO: Ozone therapy, and PBO: Placebo) on VAS at the following time points: A) 6 months, and B) 12 months. The vertical line represents the pooled effect estimate. Contour lines indicate regions of statistical significance (p<0.01, p<0.05, and p<0.10). Asymmetry in the distribution of studies may suggest potential publication bias or small-study effects...124

Figure S86. Network plot representing direct comparisons among interventions included in the network meta-analysis for VAS at the following time points: A) 6 months, and B) 12 months. PRP treatments were categorized according to Mishra's classification system vs different non-surgical control treatments (ACE: Acetaminophen, ARTHRO: Arthroscopy, BMC: Bone Marrow Concentrate, COR: Corticosteroids, HA: Hyaluronic Acid, MFAT: Microfragmented Adipose Tissue, NSAID: Non-steroidal Anti-Inflammatory Drugs, OZO: Ozone therapy, and PBO: Placebo). Node size is proportional to the total number of participants receiving each intervention, and edge thickness reflects the number of studies contributing to each direct comparison...125

Figure S87. Node-split forest plot representing inconsistency assessment in the network meta-analysis of the effect of PRP categorized according to Mishra's classification system vs different non-surgical control treatments (ACE: Acetaminophen, ARTHRO: Arthroscopy, BMC: Bone Marrow Concentrate, COR: Corticosteroids, HA: Hyaluronic Acid, MFAT: Microfragmented Adipose Tissue, NSAID: Non-steroidal Anti-Inflammatory Drugs, OZO: Ozone therapy, and PBO: Placebo) on VAS at the following time points: A) 6 months, and B) 12 months. Each treatment comparison includes both direct and indirect estimates for mean difference (MD) with corresponding 95% confidence intervals.........................126

Figure S88. Direct evidence plot representing direct evidence proportion for each assessment corresponding to the network meta-analysis of the effect of PRP categorized according to Mishra's classification system vs different non-surgical control treatments (ACE: Acetaminophen, ARTHRO: Arthroscopy, BMC: Bone Marrow Concentrate, COR: Corticosteroids, HA: Hyaluronic Acid, MFAT: Microfragmented Adipose Tissue, NSAID: Non-steroidal Anti-Inflammatory Drugs, OZO: Ozone therapy, and PBO: Placebo) on VAS at the following time points: A) 6 months, and B) 12 months. Key geometry metrics depicted include minimal path length, representing the shortest distance between nodes, and mean path length, reflecting the shortest parallel connections between nodes, and mean path length, quantifying the average shortest path across all pairs of interventions within the network...127

IKDC..128

Figure S89. Contour enhanced funnel plot for trials of the effect of PRP categorized according to Mishra's classification system vs different non-surgical control treatments (BMC: Bone Marrow Concentrate, COR: Corticosteroids, HA: Hyaluronic Acid, MFAT: Microfragmented Adipose Tissue, PBO: Placebo, and SEP: Structured Exercise Program) on IKDC at the following time points: A) 6 months, and B) 12 months. The vertical line represents the pooled effect estimate. Contour lines indicate regions of statistical significance (p<0.01, p<0.05, and p<0.10). Asymmetry in the distribution of studies may suggest potential publication bias or small-study effects..129

Figure S90. Network plot representing direct comparisons among interventions included in the network meta-analysis for IKDC at the following time points: A) 6 months, and B) 12 months. PRP treatments were categorized according to Mishra's classification system vs different non-surgical control treatments (BMC: Bone Marrow Concentrate, COR: Corticosteroids, HA: Hyaluronic Acid, MFAT: Microfragmented Adipose Tissue, PBO: Placebo, and SEP: Structured Exercise Program). Node size is proportional to the total number of participants receiving each intervention, and edge thickness reflects the number of studies contributing to each direct comparison..130

Figure S91. Node-split forest plot representing inconsistency assessment in the network meta-analysis of the effect of PRP categorized according to Mishra's classification system vs different non-surgical control treatments (BMC: Bone Marrow Concentrate, COR: Corticosteroids, HA: Hyaluronic Acid, MFAT: Microfragmented Adipose Tissue, PBO: Placebo, and SEP: Structured Exercise Program) on IKDC at the following time points: A) 6 months, and B) 12 months. Each treatment comparison includes both direct and indirect estimates for mean difference (MD) with corresponding 95% confidence intervals..131

Figure S92. Direct evidence plot representing direct evidence proportion for each assessment corresponding to the network meta-analysis of the effect of PRP categorized according to Mishra's classification system vs different non-surgical control treatments (BMC: Bone Marrow Concentrate, COR: Corticosteroids, HA: Hyaluronic Acid, MFAT: Microfragmented Adipose Tissue, PBO: Placebo, and SEP: Structured Exercise Program) on IKDC at the following time points: A) 6 months, and B) 12 months. Key geometry metrics depicted include minimal path length, representing the shortest distance between nodes, and mean path length, reflecting the shortest parallel connections between nodes, and mean path length, quantifying the average shortest path across all pairs of interventions within the network..132

Search strategy:

- **PUBMED**

(("platelet rich plasma"[MeSH Terms] OR ("platelet rich"[All Fields] AND "plasma"[All Fields]) OR "platelet rich plasma"[All Fields] OR ("platelet"[All Fields] AND "rich"[All Fields] AND "plasma"[All Fields]) OR "platelet rich plasma"[All Fields] OR ("platelet rich plasma"[MeSH Terms] OR ("platelet rich"[All Fields] AND "plasma"[All Fields]) OR "platelet rich plasma"[All Fields] OR ("platelet"[All Fields] AND "rich"[All Fields] AND "plasma"[All Fields]) OR "platelet rich plasma"[All Fields]) OR (("blood platelets"[MeSH Terms] OR ("blood"[All Fields] AND "platelets"[All Fields]) OR "blood platelets"[All Fields] OR "platelet"[All Fields] OR "platelets"[All Fields] OR "platelet s"[All Fields] OR "plateletes"[All Fields]) AND "gel"[All Fields]) OR "platelet-concentrate"[All Fields] OR (("blood platelets"[MeSH Terms] OR ("blood"[All Fields] AND "platelets"[All Fields]) OR "blood platelets"[All Fields] OR "platelet"[All Fields] OR "platelets"[All Fields] OR "platelet s"[All Fields] OR "plateletes"[All Fields]) AND ("fibrin"[MeSH Terms] OR "fibrin"[All Fields] OR "fibrins"[All Fields] OR "fibrine"[All Fields])) OR (("blood platelets"[MeSH Terms] OR ("blood"[All Fields] AND "platelets"[All Fields]) OR "blood platelets"[All Fields] OR "platelet"[All Fields] OR "platelets"[All Fields] OR "platelet s"[All Fields] OR "plateletes"[All Fields]) AND "gel"[All Fields]) OR (("plasma"[MeSH Terms] OR "plasma"[All Fields] OR "plasmas"[All Fields] OR "plasma s"[All Fields]) AND (rich, in[Author] OR rich in[Author]) AND ("intercellular signaling peptides and proteins"[MeSH Terms] OR ("intercellular"[All Fields] AND "signaling"[All Fields] AND "peptides"[All Fields] AND "proteins"[All Fields]) OR "intercellular signaling peptides and proteins"[All Fields] OR ("growth"[All Fields] AND "factors"[All Fields]) OR "growth factors"[All Fields])) OR (("preparate"[All Fields] OR "preparates"[All Fields] OR "preparation"[All Fields] OR "preparations"[All Fields] OR "preparative"[All Fields] OR "preparatively"[All Fields] OR "prepare"[All Fields] OR "prepared"[All Fields] OR "prepares"[All Fields] OR "preparing"[All Fields]) AND (rich, in[Author] OR rich in[Author]) AND ("intercellular signaling peptides and proteins"[MeSH Terms] OR ("intercellular"[All Fields] AND "signaling"[All Fields] AND "peptides"[All Fields] AND "proteins"[All Fields]) OR "intercellular signaling peptides and proteins"[All Fields] OR ("growth"[All Fields] AND "factors"[All Fields]) OR "growth factors"[All Fields])) OR "prgf"[All Fields] OR ("platelet rich fibrin"[MeSH Terms] OR ("platelet rich"[All Fields] AND "fibrin"[All Fields]) OR "platelet rich fibrin"[All Fields] OR ("platelet"[All Fields] AND "rich"[All Fields] AND "fibrin"[All Fields]) OR "platelet rich fibrin"[All Fields]) OR ("platelet rich fibrin"[MeSH Terms] OR ("platelet rich"[All Fields] AND "fibrin"[All Fields]) OR "platelet rich fibrin"[All Fields] OR ("platelet"[All Fields] AND "rich"[All Fields] AND "fibrin"[All Fields]) OR "platelet rich fibrin"[All Fields]) OR (("blood platelets"[MeSH Terms] OR ("blood"[All Fields] AND "platelets"[All Fields]) OR "blood platelets"[All Fields] OR "platelet"[All Fields] OR "platelets"[All Fields] OR "platelet s"[All Fields] OR "plateletes"[All Fields]) AND ("lysate"[All Fields] OR "lysated"[All Fields] OR "lysates"[All Fields])) OR ("platelet-leukocyte"[All Fields] AND "gel"[All Fields]) OR (("blood platelets"[MeSH Terms] OR ("blood"[All Fields] AND "platelets"[All Fields]) OR "blood platelets"[All Fields] OR "platelet"[All Fields] OR "platelets"[All Fields] OR "platelet s"[All Fields] OR "plateletes"[All Fields]) AND ("releasate"[All Fields] OR "releasates"[All Fields]))) AND "osteoarthritis"[Title] AND

"knee"[Title]) NOT "systematic"[Title] NOT "in vitro"[Title] NOT "review"[Title] NOT "letter"[Publication Type] NOT "letter"[Title]

- **SCOPUS**

TITLE-ABS ((((platelet AND rich AND plasma) OR (platelet-rich AND plasma) OR (platelet AND gel) OR (platelet AND concentrate) OR (platelet AND fibrin) OR (platelet AND gel) OR (plasma AND rich AND in AND growth AND factors) OR (preparation AND rich AND in AND growth AND factors) OR (prgf) OR (platelet AND rich AND fibrin) OR (platelet-rich AND fibrin) OR (platelet AND lysate) OR (platelet-leukocyte AND gel) OR (platelet AND releasates)) AND ((osteoarthritis AND knee))) AND NOT ("systematic" OR "review" OR "in vitro" OR "letter"))

- **EMBASE**

(((platelet rich plasma or platelet-rich plasma or platelet gel or platelet-concentrate or platelet fibrin or platelet gel or plasma rich in growth factors or preparation rich in growth factors or prgf or platelet rich fibrin or platelet-rich fibrin or platelet lysate or platelet-leukocyte gel or platelet releasates) and (osteoarthritis and knee)) not (systematic or review or in vitro or letter)).ti.

- **Cochrane Central Register of Controlled Trials (CENTRAL)**

(platelet rich plasma) OR (platelet-rich plasma) OR (platelet gel) OR (platelet-concentrate) OR (platelet fibrin) OR (platelet gel) OR (plasma rich in growth factors) OR (preparation rich in growth factors) OR prgf OR (platelet rich fibrin) OR (platelet-rich fibrin) OR (platelet lysate) OR (platelet-leukocyte gel) OR (platelet releasates) in Title Abstract Keyword AND osteoarthritis AND knee in Title Abstract Keyword NOT "systematic" OR "in vitro" OR "review" OR "letter" in Title Abstract Keyword - (Word variations have been searched)

PRP Characterization

Table S1. Characterization of whole blood and platelet-rich plasma of the studies included in the systematic review.

Ref	First Author	Blood						Platelet-rich plasma									
		PLA ($x10^9$/L)	WBC ($x10^9$/L)	ERI ($x10^{12}$/L)	LYM (%)	MON (%)	NEU (%)	PLA ($x10^9$/L)	WBC ($x10^9$/L)	ERI ($x10^{12}$/L)	LYM (%)	MON (%)	NEU (%)	PLA (fold)	Platelet Recovery Rate (%)	LEU (fold)	ERI (fold)
(54)	Acosta-Olivo	NR	NR	NR	NR	NR	NR	NR	NR	NR	NR	NR	NR	9.3	90	5	NR
(55)	Anz	238	8.5	4.03	NR	NR	NR	1216	15	0.12	NR	NR	NR	5	NR	1.73	0.03
(56)	Arliani	238 ± 38	NR	NR	NR	NR	NR	470 ± 45	NR	Free	NR	NR	NR	2.1 ± 0.2	60 ± 10	0.6 ± 0.1	NR
(57)	Bansal	230	NR	NR	NR	NR	NR	1438 ± 176	0	NR	NR	NR	NR	NR	90	NR	NR
(58)	Baria (2022)	177.6 ± 43.7	5.4 ± 1.5	3.7 ± 0.8	30.8 ± 8.3	8.8 ± 1.7	57.3 ± 9.7	2673.7 ± 1139.0	25.4 ± 13.3	0.4 ± 0.7	67.8 ± 12.5	18.6 ± 6.1	57.3 ± 9.7	NR	NR	NR	NR
(59)	Baria (2024)	186.1 ± 47.5	5.5 ± 1.5	3.6 ± 0.7	32.9 ± 7.3	8.9 ± 1.6	54.7 ± 8.5	2977.1 ± 1046.3	27.9 ± 12.8	0.2 ± 0.1	70.6 ± 9.9	17.9 ± 4.9	10.5 ± 9.4	NR	NR	NR	NR
(60)	Bennell	271.0	6.6	4.8	NR	NR	NR	325	1.2	0.03	NR	NR	NR	NR	80	NR	NR
(61)	Buendía-López	283 ± 6	NR	NR	NR	NR	NR	1095 ± 23	LP-PRP	NR	NR	NR	NR	3.87	NR	NR	NR
(62)	Chu	193.5 ± 52.1	6.3 ± 2.1	NR	NR	NR	NR	832.1 ± 269.3	0.4 ± 0.5	NR	NR	NR	NR	4.3 (3.6–4.5)	NR	NR	NR
(63)	Ciapini	NR	NR	NR	NR	NR	NR	NR	10-13%	<0.3%	NR	NR	NR	1.60	NR	0.2	0.007
(64)	Cole	NR	NR	NR	NR	NR	NR	NR	0.79 ± 0.77	NR	NR	NR	NR	1.73 ± 0.35	NR	NR	NR
(65)	Dulic	320.4	7.1	NR	NR	NR	NR	2179.3	16.16	NR	NR	NR	NR	7.23	NR	2.2	NR
(66)	Duymus	238	1%	93%	NR	NR	NR	1600-2100	1%	5%	NR	NR	NR	7-9	NR	NR	NR
(67)	Elik	NR	NR	NR	NR	NR	NR	900-1100	5-10	NR	NR	NR	NR	4-6	NR	1	NR
(68)	Elksniņš-Finogejevs	NR	NR	NR	NR	NR	NR	NR	0.2%	2.8%	NR	NR	NR	2.9	NR	NR	NR

(continued on next page)

Table S1. *continued*

Ref	First Author	Blood PLA (x10⁹/L)	Blood WBC (x10⁹/L)	Blood ERI (x10¹²/L)	Blood LYM (%)	Blood MON (%)	Blood NEU (%)	Platelet-rich plasma PLA (x10⁹/L)	Platelet-rich plasma WBC (x10⁹/L)	Platelet-rich plasma ERI (x10¹²/L)	Platelet-rich plasma LYM (%)	Platelet-rich plasma MON (%)	Platelet-rich plasma NEU (%)	PLA (fold)	Platelet Recovery Rate (%)	LEU (fold)	ERI (fold)
(69)	Filardo	NR	NR	NR	NR	NR	NR	NR	NR	NR	NR	NR	NR	4.6 ± 1.4	NR	1.1 ± 0.5	NR
(70)	Forogh	283	NR	NR	NR	NR	NR	1501	NR	NR	NR	NR	NR	5.30	NR	NR	NR
(71)	Ghai	227.35 ± 9.4	5.1 ± 0.9	NR	NR	NR	NR	310.1	NR	NR	NR	NR	NR	NR	NR	NR	NR
(72)	Görmeli	NR	NR	NR	NR	NR	NR	1118	NR	NR	NR	NR	NR	5.2	NR	NR	NR
(73)	Huang	NR	NR	NR	NR	NR	NR	NR	NR	NR	NR	NR	NR	2	80	<1	NR
(74)	Joshi Jubert	NR	NR	NR	NR	NR	NR	990	0.6	NR	NR	NR	NR	NR	NR	NR	NR
(75)	Karaborklu	201.1	NR	NR	NR	NR	NR	673.9	1.65	0.31	NR	NR	NR	3.4	76	NR	NR
(76)	Kaszynski	204 ± 48	NR	NR	NR	NR	NR	1720 ± 170	NR	NR	NR	NR	NR	8.4	NR	NR	NR
(77)	Küçükakkaş	NR	NR	NR	NR	NR	NR	1743 ± 145	6.5 ±0.8	negligible levels	NR	NR	NR	7.7 ± 3.5	NR	NR	NR
(78)	Lamo de Espinosa	NR	NR	NR	NR	NR	NR	NR	NR	NR	NR	NR	NR	2-3	NR	NR	NR
(79)	Lana	155 - 315	NR	NR	NR	NR	NR	800 - 1600	9.7 ± 3.4	NR	NR	NR	NR	5 - 8	NR	NR	NR
(80)	Lewis	238 ± 38	NR	NR	NR	NR	NR	470 ± 45	NR	Free	NR	NR	NR	2.1 ± 0.2	60 ± 10	0.6 ± 0.1	NR
(81)	Li	209.35 ± 33.0	5.8 ± 1.6	NR	NR	NR	NR	826.4 ± 41.3	15.8 ± 3.3	NR	NR	NR	NR	4	NR	2.5-2.9	NR
(82)	Lin	NR	NR	NR	NR	NR	NR	NR	30%	<0.1%	NR	NR	NR	1.8 ± 0.3	90	0.6	0.01
(83)	Louis	242 ± 60	6.4 ± 1.4	4.69 ± 0.4	NR	NR	NR	800 ± 276	0.86 ± 1.24	0.08 ± 0.05	NR	NR	17.1 ± 12.6	3.3 ± 0.7	23.2 ± 6.7	0.1 ± 0.2	NR
(84)	Malanin	263.5 ± 13.1	5.0 ± 0.1	4.6 ± 0.2	NR	NR	NR	912 ± 40	NR	NR	NR	NR	NR	NR	NR	NR	NR

(continued on next page)

Table S1. *continued*

Ref	First Author	Blood PLA (x10⁹/L)	Blood WBC (x10⁹/L)	Blood ERI (x10¹²/L)	Blood LYM (%)	Blood MON (%)	Blood NEU (%)	Platelet-rich plasma PLA (x10⁹/L)	Platelet-rich plasma WBC (x10⁹/L)	Platelet-rich plasma ERI (x10¹²/L)	Platelet-rich plasma LYM (%)	Platelet-rich plasma MON (%)	Platelet-rich plasma NEU (%)	PLA (fold)	Platelet Recovery Rate (%)	LEU (fold)	ERI (fold)
(85)	Montanez-Heredia	177 ± 56	NR	NR	NR	NR	NR	952 ± 371	0.4 ± 0.5	0.05 ± 0.03	NR	NR	NR	5.6 ± 1.8	NR	NR	NR
(86)	Nunes-Tamashiro	244 ± 43.4	NR	NR	NR	NR	NR	1119.5 ± 152.9	NR	NR	NR	NR	NR	4.61 ± 0.37	NR	NR	NR
(87)	Park	238 ± 56	6.3 ± 1.3	4.4 ± 0.5	NR	NR	NR	935 ± 354	29.38 ± 8.72	0.9 ± 0.7	NR	NR	NR	NR	NR	NR	NR
(88)	Patel	NR	NR	NR	NR	NR	NR	310.14	0.00	NR	NR	NR	NR	NR	NR	NR	NR
(89)	Raeissadat (2015)	NR	NR	NR	NR	NR	NR	NR	7.8 ± 11.3	NR	NR	NR	NR	5.2 ± 1.5	NR	NR	NR
(90)	Raeissadat (2020)	NR	NR	NR	NR	NR	NR	NR	NR	NR	NR	NR	NR	4.6 ± 0.7	NR	NR	NR
(91)	Reyes-Sosa	NR	NR	NR	NR	NR	NR	NR	NR	NR	NR	NR	NR	2-3	NR	NR	NR
(92)	Sánchez	NR	NR	NR	NR	NR	NR	NR	NR	NR	NR	NR	NR	2-3	NR	NR	NR
(93)	Sdeek	324 ± 25	NR	NR	NR	NR	NR	2664 ± 970	NR	NR	NR	NR	NR	8.2	NR	NR	NR
(94)	Simental-Mendia	251.1 ± 69.1	6.9 ± 2.2	NR	NR	NR	NR	513.25 ± 189.3	0.52 ± 0.46	NR	NR	NR	NR	2.0	NR	0.075	NR
(95)	Singh	241	NR	NR	NR	NR	NR	1019	NR	NR	NR	NR	NR	3-5	NR	NR	NR
(96)	Smith	238 ± 38	NR	NR	NR	NR	NR	470 ± 45	NR	Free	NR	NR	NR	2.1 ± 0.2	60 ± 10	0.6 ± 0.1	NR
(97)	Spakova	150 ± 30	6.4 ± 2.3	3.8 ± 0.6	NR	NR	NR	680 ± 132	23.2 ± 7.6	1.4 ± 0.9	NR	NR	NR	4.5	NR	3.6	0.4
(98)	Su	140.7 ± 11.3	5.3 ± 0.5	NR	NR	NR	NR	789.68 ± 17.80	29.92 ± 1.54	NR	NR	NR	NR	5.6 ± 0.5	NR	NR	NR
(99)	Tschopp	238 ± 38	NR	NR	NR	NR	NR	470 ± 45	NR	Free	NR	NR	NR	2.1 ± 0.2	60 ± 10	0.6 ± 0.1	NR
(100)	Tucker	264.9 ± 62.8	6.8 ± 2.5	4.4 ± 0.8	NR	NR	NR	703.7 ± 308.3	2.6 ±1.0	0	NR	NR	NR	2.6 ± 0.7	NR	NR	NR

(continued on next page)

Table S1. *continued*

		Blood						Platelet-rich plasma									
Ref	First Author	PLA (x10^9/L)	WBC (x10^9/L)	ERI (x10^{12}/L)	LYM (%)	MON (%)	NEU (%)	PLA (x10^9/L)	WBC (x10^9/L)	ERI (x10^{12}/L)	LYM (%)	MON (%)	NEU (%)	PLA (fold)	Platelet Recovery Rate (%)	LEU (fold)	ERI (fold)
(101)	Uslu Güvendi	245	7.5	NR	NR	NR	NR	875	8.7	NR	NR	NR	NR	3.6	NR	1.2	NR
(102)	Vaquerizo	NR	NR	NR	NR	NR	NR	NR	NR	NR	NR	NR	NR	2-3	NR	NR	NR
(103)	Wang	NR	NR	NR	NR	NR	NR	NR	NR	NR	NR	NR	NR	2-6	85	Low	0.01
(104)	Wu	NR	NR	NR	NR	NR	NR	NR	30-40%	<0.1%	NR	NR	NR	1.7-1.8	NR	0.6	0.01
(105)	Xu	185 ± 45	NR	NR	NR	NR	NR	950 ± 173	NR	NR	NR	NR	NR	5.13	NR	NR	NR
(106)	Yaradilmis (L-PRP)	252.4	NR	NR	NR	NR	NR	1178 ± 321	43.6 ± 13.8	NR	NR	NR	53	4.6	NR	NR	NR
(106)	Yaradilmis (P-PRP)	247.3	NR	NR	NR	NR	NR	476 ± 127	0.9 ± 1.5	NR	NR	NR	28	1.9	NR	NR	NR
(107)	Yoshioka	240.2 ± 42.8	5.1 ± 0.8	4.5 ± 0.2	NR	NR	NR	475.4 ± 106.7	0.004 ± 0.02	0.007 ± 0.008	NR	NR	NR	2.0 ± 0.2	43.9 ± 4.4	0.0007 ± 0.003	NR
(108)	Yurtbay	NR	NR	NR	NR	NR	NR	1280	9-11	NR	NR	NR	NR	>5	NR	NR	NR
(109)	Zaffagnini	NR	NR	NR	NR	NR	NR	NR	NR	NR	NR	NR	NR	5.0	NR	1.5	NR

Table S2. PRP dosing and administration regimen.

Ref	First Author	Year	Image Guidance	Needle (G)[a]	Volume injected (mL)	Dose/infiltration (x10⁹/L)			Series of infiltration (n)	Injection timing (weeks)[e]	Total dose (x10⁹/L)[a]		
						PLA[b]	WBC[c]	ERI[d]			PLA[b]	WBC[c]	ERI[d]
(54)	Acosta-Olivo	2014	NO	NR	5	9.3	0.18	NR	2	2	18.6	0.36	NR
(55)	Anz	2022	US-guided	NR	7	8.5	0.10	0.84	1	NA	8.5	0.10	0.84
(56)	Arliani	2022	NO	NR	5	2.4	0.02	0	3	1	7.1	0.07	0
(57)	Bansal	2021	NO	NR	8	10.5	0	NR	1	NA	10.5	0	NR
(58)	Baria	2022	US-guided	25	5.1 ± 1.1	13.7	0.13	NR	1	NA	13.7	0.13	NR
(59)	Baria	2024	US-guided	25	5.2 ± 1.1	15.5	0.14	1.04	1	NA	15.5	0.14	1.04
(60)	Bennell	2021	US-guided	22	5	1.6	0.01	0.15	3	1	4.9	0.02	0.45
(61)	Buendía-López	2018	NO	NR	5	5.5	NR	NR	1	NA	5.5	NR	NR
(62)	Chu	2022	NO	NR	5	4.2	0	NR	3	1	12.5	0.01	NR
(63)	Ciapini	2023	NO	NR	4	1.3	0.01	0	3	4	3.8	0.02	0
(64)	Cole	2017	US-guided	NR	4	1.4	0	NR	3	1	4.1	0.01	NR
(65)	Dulic	2021	NO	NR	6	13.1	0.10	NR	1	NA	13.1	0.10	NR
(66)	Duymus	2017	NO	22	5	9.3	NR	NR	2	4	18.5	NR	NR
(67)	Elik	2020	NO	21	4	4.0	0.03	NR	3	1	12.0	0.09	NR
(68)	Elksniņš-Finogejevs	2020	US-guided	20	8	4.6	NR	NR	1	NA	4.6	NR	NR
(69)	Filardo	2015	NO	NR	5	4.6	0.04	NR	3	1	13.8	0.12	NR

(continued on next page)

Table S2. *Continued*

Ref	First Author	Year	Image Guidance	Needle (G)[a]	Volume injected (mL)	Dose/infiltration (x10⁹/L)			Series of infiltration (n)	Injection timing (weeks)[e]	Total dose (x10⁹/L)[a]		
						PLA[b]	WBC[c]	ERI[d]			PLA[b]	WBC[c]	ERI[d]
(70)	Forogh	2016	NO	NR	5	7.5	NR	NR	1	NA	7.5	NR	NR
(71)	Ghai	2019	US-guided	18	8	2.38	NR	NR	1	NA	2.4	NR	NR
(72)	Görmeli	2017	NO	22	5	5.6	NR	NR	3	1	16.8	NR	NR
(73)	Huang	2019	NO	NR	4	1.6	NR	NR	3	3	4.8	NR	NR
(74)	Joshi Jubert	2017	NO	21	4	4.0	0	NR	1	NA	4.0	0	NR
(75)	Karaborklu	2024	US-guided	22	6	4.04	0.01	1.86	3	1	12.1	0.03	5.58
(76)	Kaszynski	2022	NO	21	3	5.2	NR	NR	3	2	15.5	NR	NR
(77)	Küçükakkaş	2022	NO	NR	5	8.7	0.03	NR	1	NA	8.7	0.03	NR
(78)	Lamo de Espinosa	2021	NO	NR	NR	NR	0	NR	3	1	NR	0	NR
(79)	Lana	2016	US-guided	NR	5	6.0	0.05	NR	3	2	18.0	0.15	NR
(80)	Lewis	2022	NO	NR	4-6	2.4	NR	0	3	1	7.1	NR	0
(81)	Li	2023	US-guided	NR	4	3.3	0.06	NR	3	1	9.9	0.19	NR
(82)	Lin	2019	NO	NR	2	0.7	0.01	0	3	1	2.2	0.03	0
(83)	Louis	2018	US-guided	21	3	2.4	0	0.23	1	NA	2.4	0	0.23
(84)	Malanin	2017	NO	NR	2	1.8	NR	NR	3	1	5.5	NR	NR
(85)	Montanez-Heredia	2016	NO	NR	5	4.8	0	0.25	3	2	14.3	0.01	0.75

(continued on next page)

Table S2. *Continued*

Ref	First Author	Year	Image Guidance	Needle (G)[a]	Volume injected (mL)	Dose/infiltration (x10⁹/L)			Series of infiltration (n)	Injection timing (weeks)[e]	Total dose (x10⁹/L)[a]		
						PLA[b]	WBC[c]	ERI[d]			PLA[b]	WBC[c]	ERI[d]
(86)	Nunes-Tamashiro	2022	NO	21	2	2.2	NR	NR	1	NA	2.2	NR	NR
(87)	Park	2021	NO	NR	3	2.8	0.09	2.82	1	NA	2.8	0.09	2.82
(88)	Patel	2013	NO	18	8	2.39	0	NR	1	NA	2.4	0	NR
(89)	Raeissadat	2015	NO	22	4-6	5.2	0.04	NR	2	4	10.4	0.08	NR
(90)	Raeissadat	2020	NO	21	5	4.6	NR	NR	2	3	9.2	NR	NR
(91)	Reyes-Sosa	2020	NO	NR	3	1.5	0	0	2	2	3.0	0	0
(92)	Sánchez	2012	NO	NR	8	4.0	0	0	3	1	12.0	0	0
(93)	Sdeek	2021	NO	NR	2.5	6.7	NR	NR	3	2	20.0	NR	NR
(94)	Simental-Mendia	2016	NO	22	3	1.5	0	NR	3	2	4.6	0	NR
(95)	Singh	2022	NO	22	4-5	4.6	NR	NR	1	NA	4.6	NR	NR
(96)	Smith	2016	NO	NR	3-8	2.6	0	0	3	1	7.8	0.01	0
(97)	Spakova	2012	NO	NR	3	2.0	0.07	4.20	3	1	6.1	0.21	12.60
(98)	Su	2018	NO	NR	6	4.7	0.18	NR	2	2	9.5	0.36	NR
(99)	Tschopp	2023	Fluoroscopy-guided	NR	3	1.4	NR	0	1	NA	1.4	NR	0
(100)	Tucker	2021	US-guided	18	5	3.5	0.01	0	1	NA	3.5	0.01	0
(101)	Uslu Güvendi	2018	NO	NR	2.5	2.2	0.02	NR	1	NA	3.5	0.02	NR

(continued on next page)

Table S2. *Continued*

Ref	First Author	Year	Image Guidance	Needle (G)[a]	Volume injected (mL)	Dose/infiltration ($x10^9$/L)			Series of infiltration (n)	Injection timing (weeks)[e]	Total dose ($x10^9$/L)[a]		
						PLA[b]	WBC[c]	ERI[d]			PLA[b]	WBC[c]	ERI[d]
(102)	Vaquerizo	2013	NO	22	8	4.0	0	0	3	1	12.0	0	0
(103)	Wang	2022	NO	21	4	3.2	NR	0	1	NA	3.2	NR	0
(104)	Wu	2018	NO	NR	4	1.4	0.02	0	1	NA	1.4	0.02	0
(105)	Xu	2021	US-guided	25	4	1.9	NR	NR	3	2	5.6	NR	NR
(106)	Yaradilmis (L-PRP)	2020	NO	NR	NR	NR	NR	NR	3	1	NR	NR	NR
(106)	Yaradilmis (P-PRP)	2020	NO	NR	NR	NR	NR	NR	3	1	NR	NR	NR
(107)	Yoshioka	2024	NO	21	6	2.9	0	0.04	3	1	8.7	0	0.13
(108)	Yurtbay	2022	NO	NR	5	6.4	0.05	NR	1	NA	6.4	0.05	NR
(109)	Zaffagnini	2022	NO	22	5	5.0	0.05	NR	1	NA	5.0	0.05	NR

[a] *NR: Not reported*

[b] *PLA: Platelets*

[c] *WBC: White Blood Cells*

[d] *ERI: Erythrocytes*

[e] *NA: Not applicable*

Table S3. Classification of platelet-rich plasma applied in the studies included in the systematic review according to Mishra's classification system[44]

Reference	First Author	Year	Mishra's PRP classification
(54)	Acosta-Olivo	2014	1A
(55)	Anz	2022	1A
(56)	Arliani	2022	3B
(57)	Bansal	2021	3A
(58)	Baria	2022	1A
(59)	Baria	2024	1A
(60)	Bennell	2021	3B
(61)	Buendía-López	2018	4A
(62)	Chu	2022	3B
(63)	Ciapini	2023	3B
(64)	Cole	2017	3B
(65)	Dulic	2021	1A
(66)	Duymus	2017	1A
(67)	Elik	2020	2B
(68)	Elksniņš-Finogejevs	2020	3B
(69)	Filardo	2015	2B
(70)	Forogh	2016	2A
(71)	Ghai	2019	4B
(72)	Görmeli	2017	2A
(73)	Huang	2019	3B
(74)	Joshi Jubert	2017	3B
(75)	Karaborklu	2024	3B
(76)	Kaszynski	2022	3A
(77)	Küçükakkaş	2022	1A
(78)	Lamo de Espinosa	2021	4B
(79)	Lana	2016	2A
(80)	Lewis	2022	3B
(81)	Li	2023	1B

(continued on next page)

Table S3. *Continued*

Reference	First Author	Year	Mishra's PRP classification
(82)	Lin	2019	3B
(83)	Louis	2018	3A
(84)	Malanin	2017	2B
(85)	Montanez-Heredia	2016	3A
(86)	Nunes-Tamashiro	2022	ND
(87)	Park	2021	1A
(88)	Patel	2013	4B
(89)	Raeissadat	2015	1A
(90)	Raeissadat	2020	2B
(91)	Reyes-Sosa	2020	4B
(92)	Sánchez	2012	4B
(93)	Sdeek	2021	3A
(94)	Simental-Mendia	2016	4B
(95)	Singh	2022	ND
(96)	Smith	2016	3B
(97)	Spakova	2012	1B
(98)	Su	2018	2A
(99)	Tschopp	2023	3B
(100)	Tucker	2021	3B
(101)	Uslu Güvendi	2018	3B
(102)	Vaquerizo	2013	4B
(103)	Wang	2022	3B
(104)	Wu	2018	3B
(105)	Xu	2021	3A
(106)	Yaradilmis (L-PRP)	2020	1A
(106)	Yaradilmis (P-PRP)	2020	3B
(107)	Yoshioka	2024	3B
(108)	Yurtbay	2022	2A
(109)	Zaffagnini	2022	2A

ND: Not determined

Table S4. Data sources for PRP characterization

Ref	First Author	Year	Data source
(54)	Acosta-Olivo	2014	Website of the manufacturer
(55)	Anz	2022	One patient of the PRP group
(56)	Arliani	2022	Website of the manufacturer
(57)	Bansal	2021	Information included in the paper
(58)	Baria	2022	Information included in the paper
(59)	Baria	2024	Information included in the paper
(60)	Bennell	2021	Information included in the paper
(61)	Buendía-López	2018	Five patients of the PRP group
(62)	Chu	2022	Information included in the paper
(63)	Ciapini	2023	Website of the manufacturer
(64)	Cole	2017	Information included in the paper
(65)	Dulic	2021	Information included in the paper
(66)	Duymus	2017	Website of the manufacturer
(67)	Elik	2020	Information included in the paper - not all patients
(68)	Elksniņš-Finogejevs	2020	Website of the manufacturer
(69)	Filardo	2015	Information included in the paper
(70)	Forogh	2016	Five healthy persons unrelated to the study
(71)	Ghai	2019	Information included in the paper
(72)	Görmeli	2017	Information included in the paper
(73)	Huang	2019	Information included in the paper
(74)	Joshi Jubert	2017	Information included in the paper
(75)	Karaborklu	2024	Information included in the paper
(76)	Kaszynski	2022	Information included in the paper
(77)	Küçükakkaş	2022	Characterization of PRP refers to other paper
(78)	Lamo de Espinosa	2021	Website of the manufacturer
(79)	Lana	2016	Information included in the paper
(80)	Lewis	2022	Website of the manufacturer
(81)	Li	2023	Information included in the paper
(82)	Lin	2019	Website of the manufacturer

(continued on next page)

Table S4. *Continued*

Ref	First Author	Year	Data source
(83)	Louis	2018	Information included in the paper
(84)	Malanin	2017	Information included in the paper
(85)	Montanez-Heredia	2016	Information included in the paper
(86)	Nunes-Tamashiro	2022	Information included in the paper
(87)	Park	2021	Information included in the paper
(88)	Patel	2013	Information included in the paper
(89)	Raeissadat	2015	Information included in the paper
(90)	Raeissadat	2020	Information included in the paper
(91)	Reyes-Sosa	2020	Website of the manufacturer
(92)	Sánchez	2012	Website of the manufacturer
(93)	Sdeek	2021	Information included in the paper
(94)	Simental-Mendia	2016	Information included in the paper
(95)	Singh	2022	Information included in the paper
(96)	Smith	2016	Website of the manufacturer
(97)	Spakova	2012	Information included in the paper
(98)	Su	2018	Information included in the paper
(99)	Tschopp	2023	Website of the manufacturer
(100)	Tucker	2021	Information included in the paper
(101)	Uslu Güvendi	2018	Characterization performed in individuals no related with RCT
(102)	Vaquerizo	2013	Website of the manufacturer
(103)	Wang	2022	Website of the manufacturer
(104)	Wu	2018	Website of the manufacturer
(105)	Xu	2021	Information included in the paper
(106)	Yaradilmis	2020	Information included in the paper
(107)	Yoshioka	2024	Information included in the paper
(108)	Yurtbay	2022	Information included in the paper
(109)	Zaffagnini	2022	Information included in the paper

Effect of PRP activation

WOMAC Pain

A)

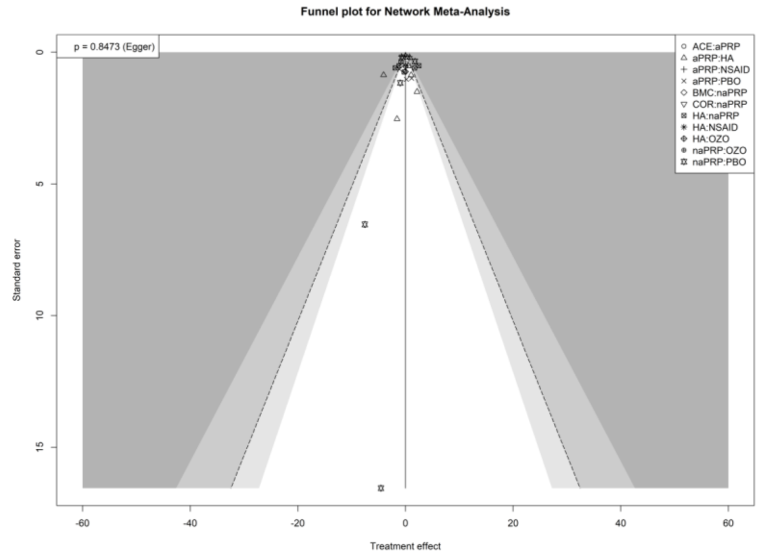

B)

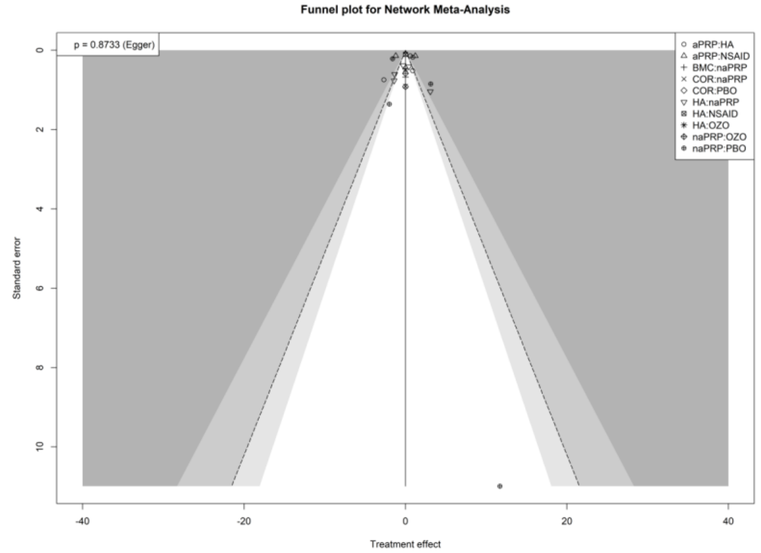

Figure S1. Contour enhanced funnel plot for trials of the effect of PRP stratified by platelet activation status (activated PRP = aPRP, and non-activated PRP = naPRP) vs different non-surgical control treatments (ACE: Acetaminophen, BMC: Bone Marrow Concentrate, COR: Corticosteroids, HA: Hyaluronic Acid, NSAID: Non-steroidal Anti-Inflammatory Drugs, and OZO: Ozone therapy) on WOMAC Pain at the following time points: A) 6 months, and B) 12 months. The vertical line represents the pooled effect estimate. Contour lines indicate regions of statistical significance (p<0.01, p<0.05, and p<0.10). Asymmetry in the distribution of studies may suggest potential publication bias or small-study effects.

A)

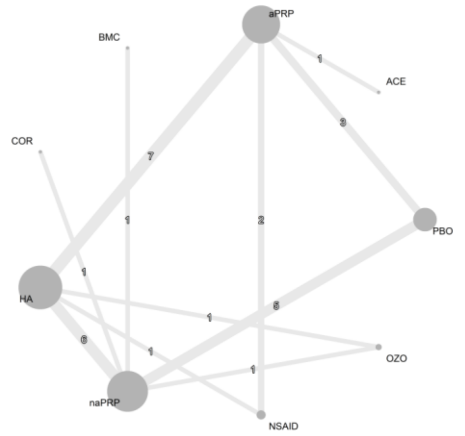

B)

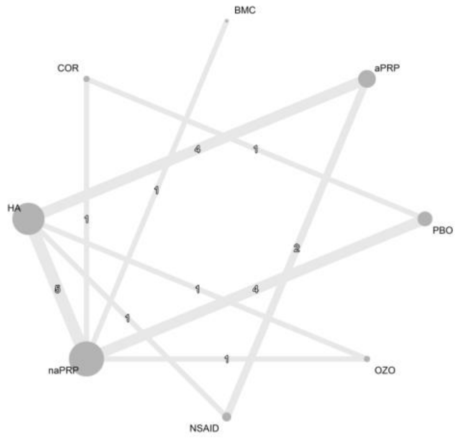

Figure S2. Network plot representing direct comparisons among interventions included in the network meta-analysis for WOMAC Pain at the following time points: A) 6 months, and B) 12 months. PRP treatments were stratified by platelet activation status (activated PRP = aPRP, and non-activated PRP = naPRP) vs different non-surgical control treatments (ACE: Acetaminophen, BMC: Bone Marrow Concentrate, COR: Corticosteroids, HA: Hyaluronic Acid, NSAID: Non-steroidal Anti-Inflammatory Drugs, and OZO: Ozone therapy). Node size is proportional to the total number of participants receiving each intervention, and edge thickness reflects the number of studies contributing to each direct comparison.

A)

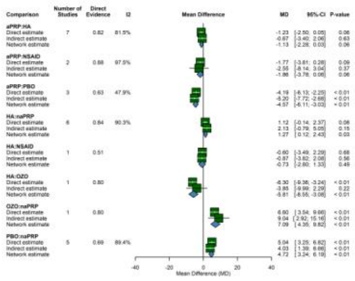

B)

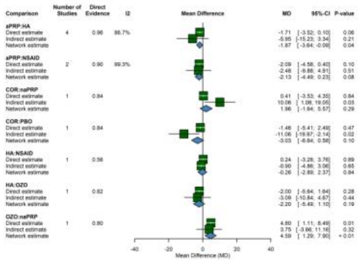

Figure S3. Node-split forest plot representing inconsistency assessment in the network meta-analysis of the effect of PRP stratified by platelet activation status (activated PRP = aPRP, and non-activated PRP = naPRP) vs different non-surgical control treatments (ACE: Acetaminophen, BMC: Bone Marrow Concentrate, COR: Corticosteroids, HA: Hyaluronic Acid, NSAID: Non-steroidal Anti-Inflammatory Drugs, and OZO: Ozone therapy) on WOMAC Pain at the following time points: A) 6 months, and B) 12 months. Each treatment comparison includes both direct and indirect estimates for mean difference (MD) with corresponding 95% confidence intervals.

A)

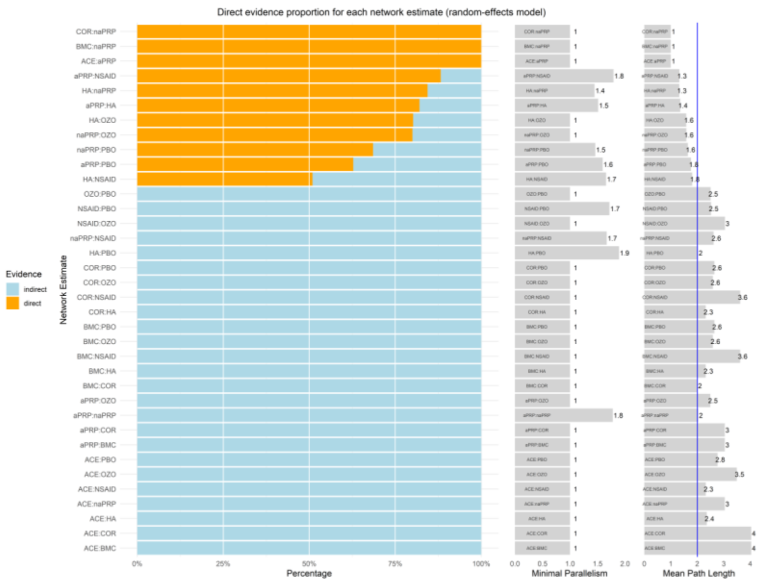

B)

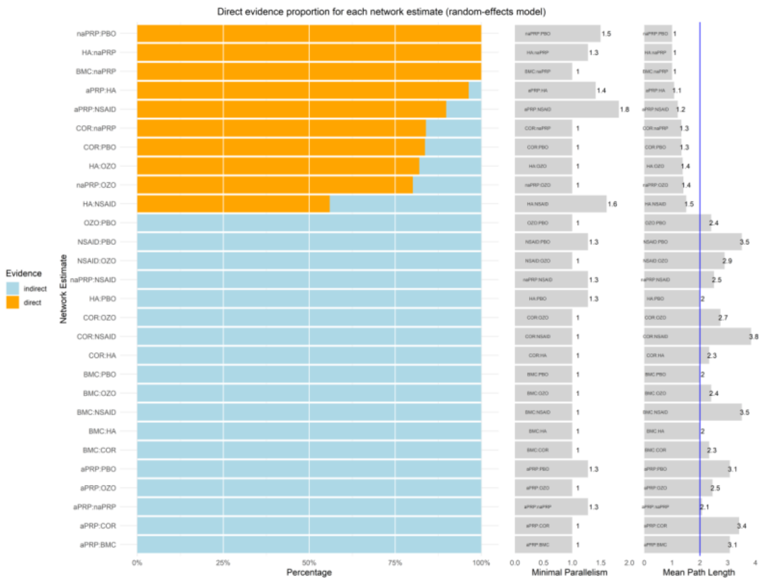

Figure S4. Direct evidence plot representing direct evidence proportion for each assessment corresponding to the network meta-analysis of the effect of PRP stratified by platelet activation status (activated PRP = aPRP, and non-activated PRP = naPRP) vs different non-surgical control treatments (ACE: Acetaminophen, BMC: Bone Marrow Concentrate, COR: Corticosteroids, HA: Hyaluronic Acid, NSAID: Non-steroidal Anti-Inflammatory Drugs, and OZO: Ozone therapy) on WOMAC Pain at the following time points: A) 6 months, and B) 12 months. Key geometry metrics depicted include minimal path length, representing the shortest distance between nodes, and mean path length, reflecting the shortest parallel connections between nodes, and mean path length, quantifying the average shortest path across all pairs of interventions within the network.

WOMAC Stiffness

A)

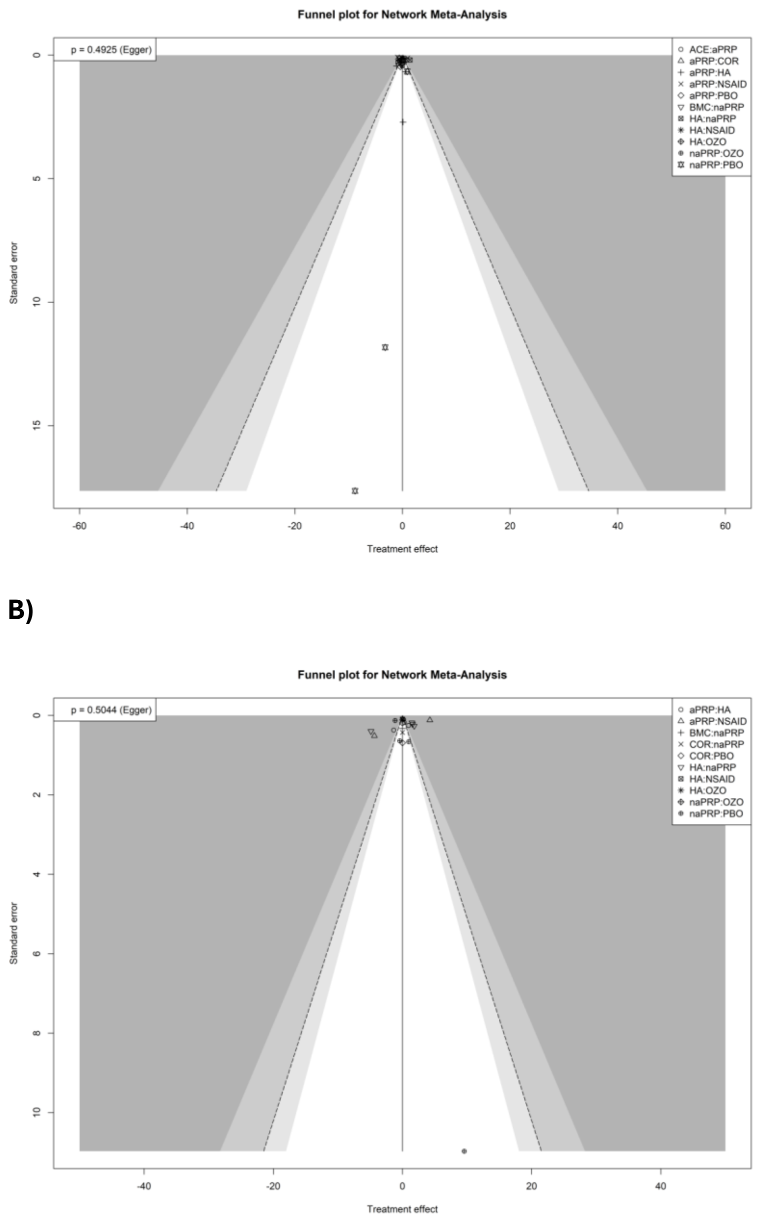

B)

Figure S5. Contour enhanced funnel plot for trials of the effect of PRP stratified by platelet activation status (activated PRP = aPRP, and non-activated PRP = naPRP) vs different non-surgical control treatments (ACE: Acetaminophen, BMC: Bone Marrow Concentrate, COR: Corticosteroids, HA: Hyaluronic Acid, NSAID: Non-steroidal Anti-Inflammatory Drugs, and OZO: Ozone therapy) on WOMAC Stiffness at the following time points: A) 6 months, and B) 12 months. The vertical line represents the pooled effect estimate. Contour lines indicate regions of statistical significance ($p<0.01$, $p<0.05$, and $p<0.10$). Asymmetry in the distribution of studies may suggest potential publication bias or small-study effects.

A)

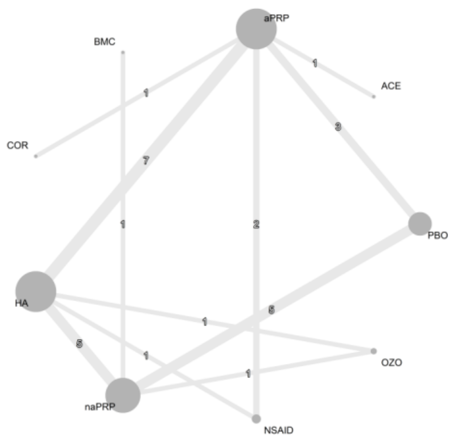

B)

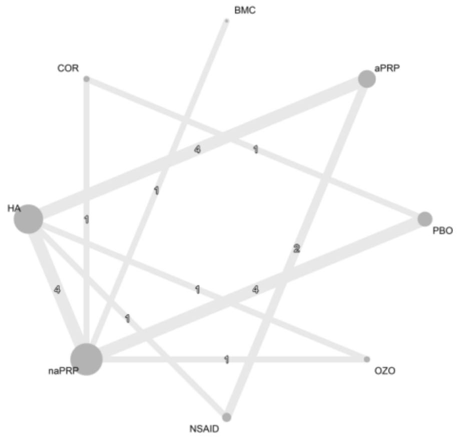

Figure S6. Network plot representing direct comparisons among interventions included in the network meta-analysis for WOMAC Stiffness at the following time points: A) 6 months, and B) 12 months. PRP treatments were stratified by platelet activation status (activated PRP = aPRP, and non-activated PRP = naPRP) vs different non-surgical control treatments (ACE: Acetaminophen, BMC: Bone Marrow Concentrate, COR: Corticosteroids, HA: Hyaluronic Acid, NSAID: Non-steroidal Anti-Inflammatory Drugs, OZO: Ozone therapy, and PBO: Placebo). Node size is proportional to the total number of participants receiving each intervention, and edge thickness reflects the number of studies contributing to each direct comparison.

A)

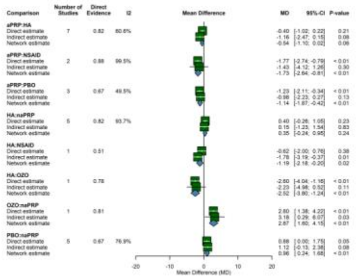

B)

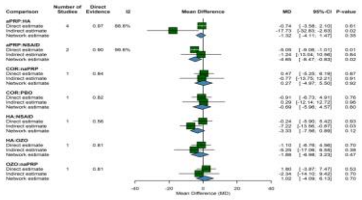

Figure S7. Node-split forest plot representing inconsistency assessment in the network meta-analysis of the effect of PRP stratified by platelet activation status (activated PRP = aPRP, and non-activated PRP = naPRP) vs different non-surgical control treatments (ACE: Acetaminophen, BMC: Bone Marrow Concentrate, COR: Corticosteroids, HA: Hyaluronic Acid, NSAID: Non-steroidal Anti-Inflammatory Drugs, OZO: Ozone therapy, and PBO: Placebo) on WOMAC Stiffness at the following time points: A) 6 months, and B) 12 months. Each treatment comparison includes both direct and indirect estimates for mean difference (MD) with corresponding 95% confidence intervals.

A)

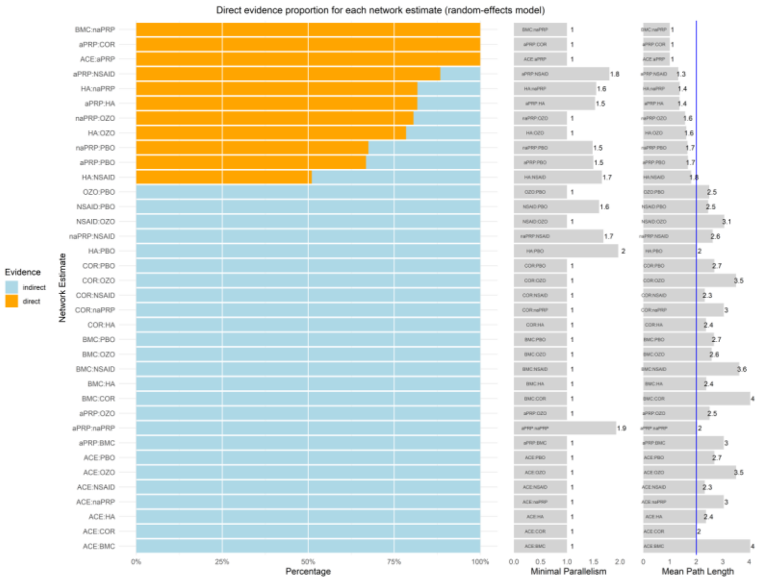

B)

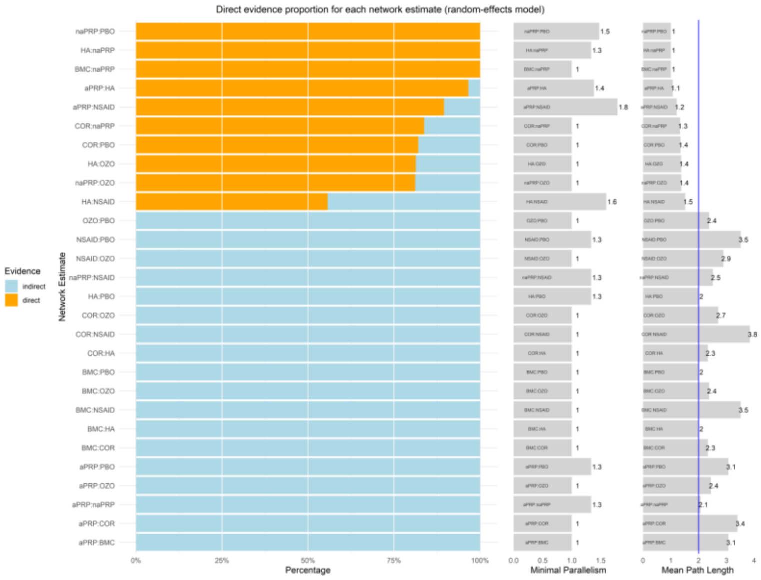

Figure S8. Direct evidence plot representing direct evidence proportion for each assessment corresponding to the network meta-analysis of the effect of PRP stratified by platelet activation status (activated PRP = aPRP, and non-activated PRP = naPRP) vs different non-surgical control treatments (ACE: Acetaminophen, BMC: Bone Marrow Concentrate, COR: Corticosteroids, HA: Hyaluronic Acid, NSAID: Non-steroidal Anti-Inflammatory Drugs, OZO: Ozone therapy, and PBO: Placebo) on WOMAC Stiffness at the following time points: A) 6 months, and B) 12 months. Key geometry metrics depicted include minimal path length, representing the shortest distance between nodes, and mean path length, reflecting the shortest parallel connections between nodes, and mean path length, quantifying the average shortest path across all pairs of interventions within the network.

WOMAC Physical Function

A)

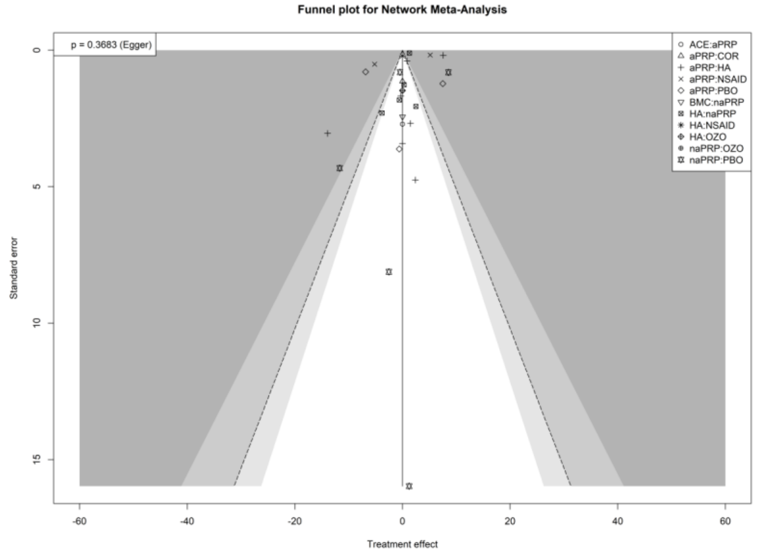

B)

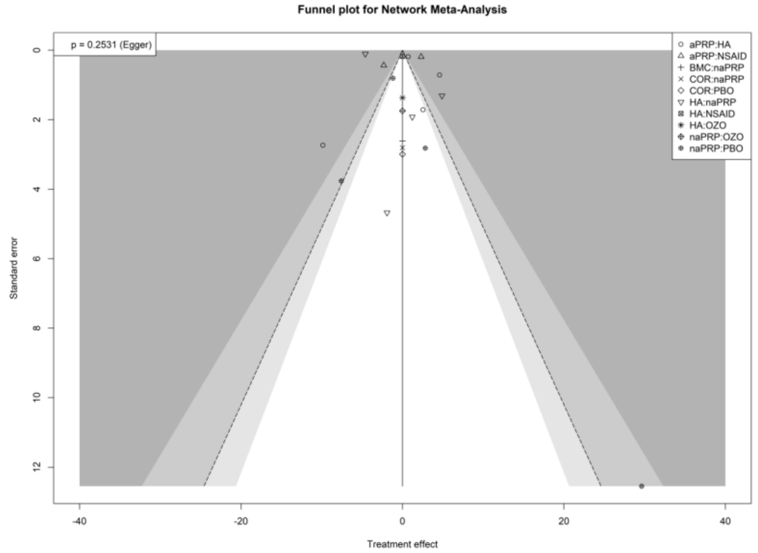

Figure S9. Contour enhanced funnel plot for trials of the effect of PRP stratified by platelet activation status (activated PRP = aPRP, and non-activated PRP = naPRP) vs different non-surgical control treatments (ACE: Acetaminophen, BMC: Bone Marrow Concentrate, COR: Corticosteroids, HA: Hyaluronic Acid, NSAID: Non-steroidal Anti-Inflammatory Drugs, OZO: Ozone therapy, and PBO: Placebo) on WOMAC Physical Function at the following time points: A) 6 months, and B) 12 months. The vertical line represents the pooled effect estimate. Contour lines indicate regions of statistical significance ($p<0.01$, $p<0.05$, and $p<0.10$). Asymmetry in the distribution of studies may suggest potential publication bias or small-study effects.

A)

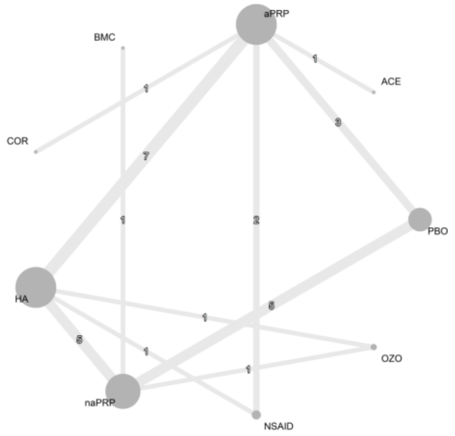

B)

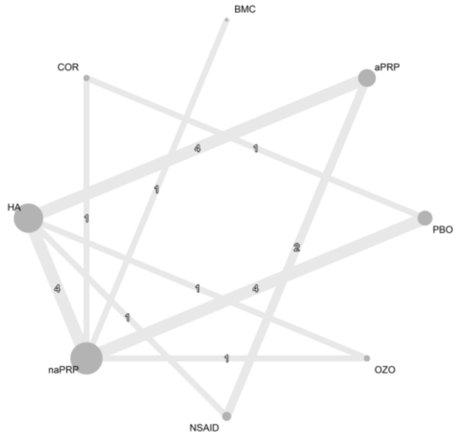

Figure S10. Network plot representing direct comparisons among interventions included in the network meta-analysis for WOMAC Physical Function at the following time points: A) 6 months, and B) 12 months. PRP treatments were stratified by platelet activation status (activated PRP = aPRP, and non-activated PRP = naPRP) vs different non-surgical control treatments (ACE: Acetaminophen, BMC: Bone Marrow Concentrate, COR: Corticosteroids, HA: Hyaluronic Acid, NSAID: Non-steroidal Anti-Inflammatory Drugs, OZO: Ozone therapy, and PBO: Placebo). Node size is proportional to the total number of participants receiving each intervention, and edge thickness reflects the number of studies contributing to each direct comparison.

A)

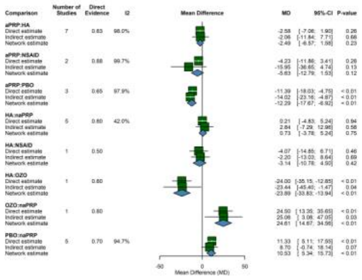

B)

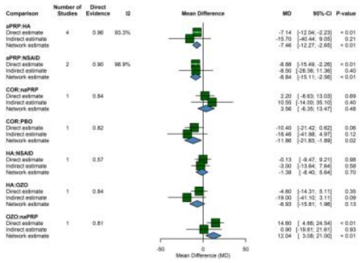

Figure S11. Node-split forest plot representing inconsistency assessment in the network meta-analysis of the effect of PRP stratified by platelet activation status (activated PRP = aPRP, and non-activated PRP = naPRP) vs different non-surgical control treatments (ACE: Acetaminophen, BMC: Bone Marrow Concentrate, COR: Corticosteroids, HA: Hyaluronic Acid, NSAID: Non-steroidal Anti-Inflammatory Drugs, OZO: Ozone therapy, and PBO: Placebo) on WOMAC Physical Function at the following time points: A) 6 months, and B) 12 months. Each treatment comparison includes both direct and indirect estimates for mean difference (MD) with corresponding 95% confidence intervals.

A)

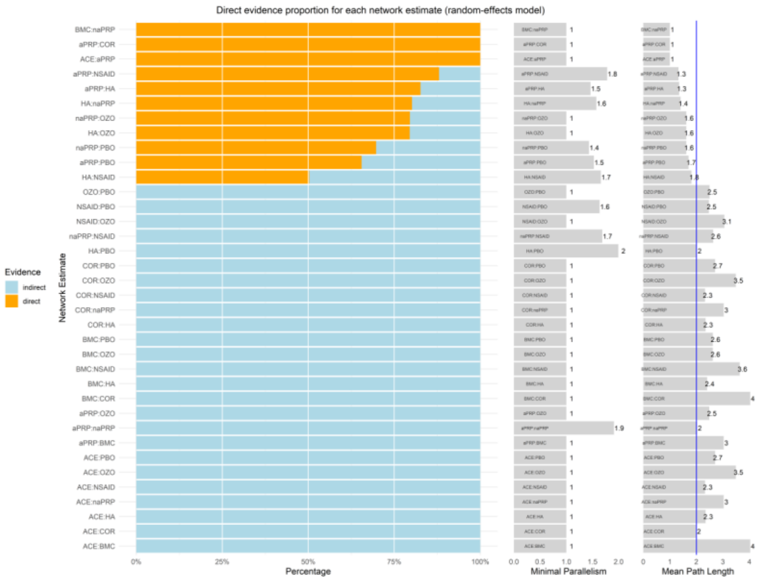

B)

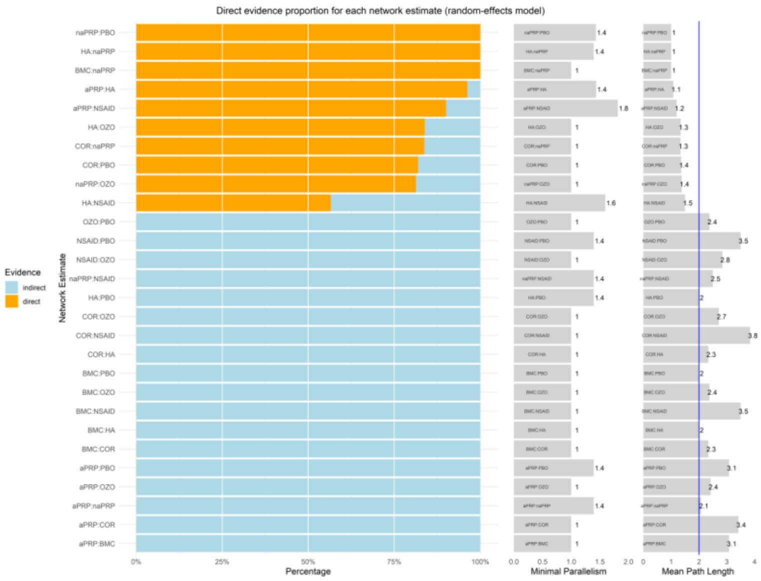

Figure S12. Direct evidence plot representing direct evidence proportion for each assessment corresponding to the network meta-analysis of the effect of PRP stratified by platelet activation status (activated PRP = aPRP, and non-activated PRP = naPRP) vs different non-surgical control treatments (ACE: Acetaminophen, BMC: Bone Marrow Concentrate, COR: Corticosteroids, HA: Hyaluronic Acid, NSAID: Non-steroidal Anti-Inflammatory Drugs, OZO: Ozone therapy, and PBO: Placebo) on WOMAC Physical Function at the following time points: A) 6 months, and B) 12 months. Key geometry metrics depicted include minimal path length, representing the shortest distance between nodes, and mean path length, reflecting the shortest parallel connections between nodes, and mean path length, quantifying the average shortest path across all pairs of interventions within the network.

WOMAC Total

A)

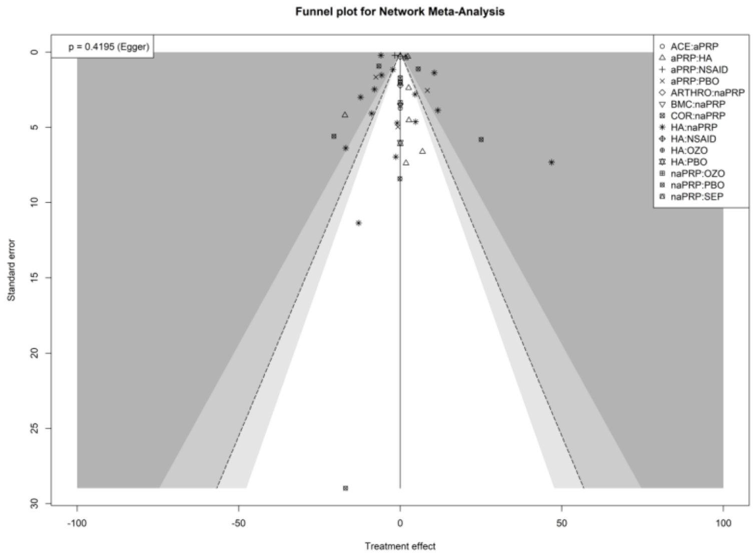

B)

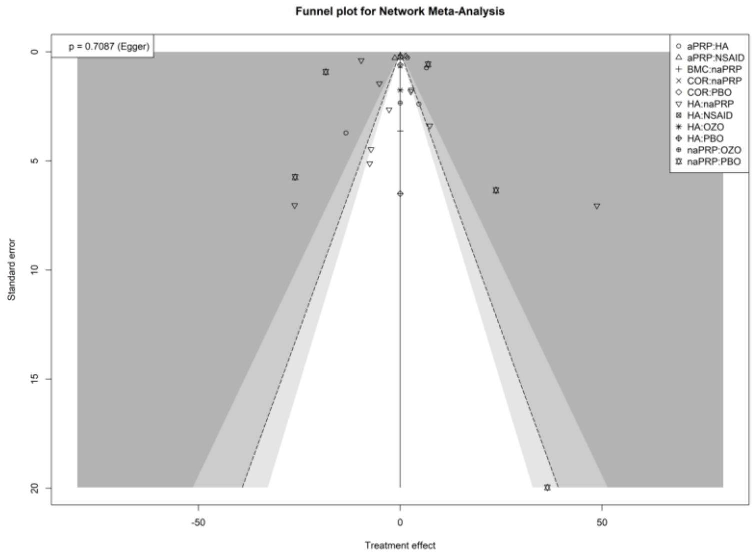

Figure S13. Contour enhanced funnel plot for trials of the effect of PRP stratified by platelet activation status (activated PRP = aPRP, and non-activated PRP = naPRP) vs different non-surgical control treatments (ACE: Acetaminophen, ARTHRO: Arthroscopy, BMC: Bone Marrow Concentrate, COR: Corticosteroids, HA: Hyaluronic Acid, NSAID: Non-steroidal Anti-Inflammatory Drugs, OZO: Ozone therapy, PBO: Placebo, and SEP: Structured Exercise Program) on WOMAC Total at the following time points: A) 6 months, and B) 12 months. The vertical line represents the pooled effect estimate. Contour lines indicate regions of statistical significance (*p*<0.01, *p*<0.05, and *p*<0.10). Asymmetry in the distribution of studies may suggest potential publication bias or small-study effects.

A)

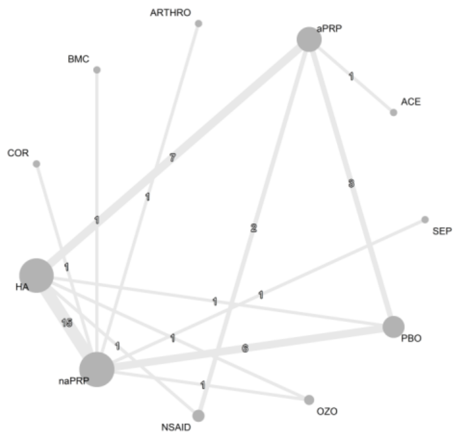

B)

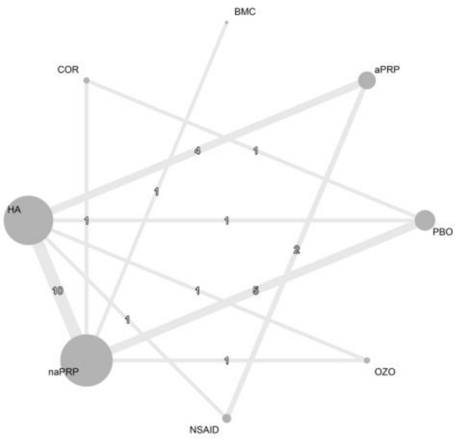

Figure S14. Network plot representing direct comparisons among interventions included in the network meta-analysis for WOMAC Total at the following time points: A) 6 months, and B) 12 months. PRP treatments were stratified by platelet activation status (activated PRP = aPRP, and non-activated PRP = naPRP) vs different non-surgical control treatments (ACE: Acetaminophen, BMC: Bone Marrow Concentrate, COR: Corticosteroids, HA: Hyaluronic Acid, NSAID: Non-steroidal Anti-Inflammatory Drugs, OZO: Ozone therapy, and PBO: Placebo). Node size is proportional to the total number of participants receiving each intervention, and edge thickness reflects the number of studies contributing to each direct comparison.

A)

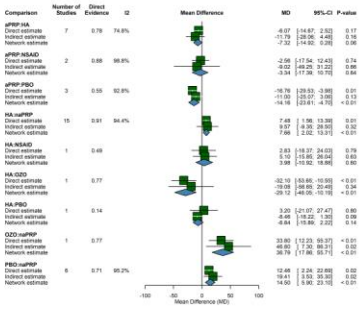

B)

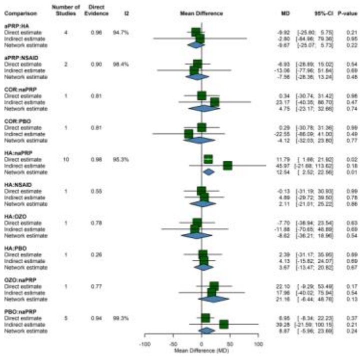

Figure S15. Node-split forest plot representing inconsistency assessment in the network meta-analysis of the effect of PRP stratified by platelet activation status (activated PRP = aPRP, and non-activated PRP = naPRP) vs different non-surgical control treatments (ACE: Acetaminophen, BMC: Bone Marrow Concentrate, COR: Corticosteroids, HA: Hyaluronic Acid, NSAID: Non-steroidal Anti-Inflammatory Drugs, OZO: Ozone therapy, and PBO: Placebo) on WOMAC Total at the following time points: A) 6 months, and B) 12 months. Each treatment comparison includes both direct and indirect estimates for mean difference (MD) with corresponding 95% confidence intervals.

A)

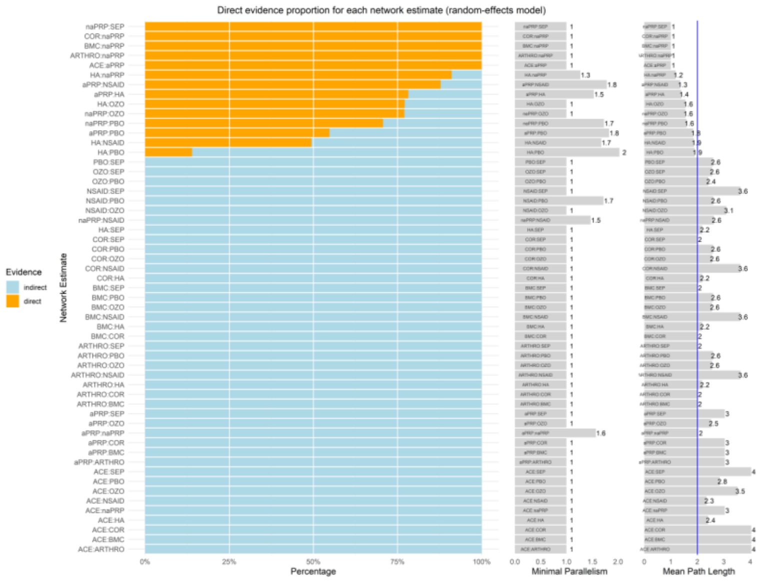

B)

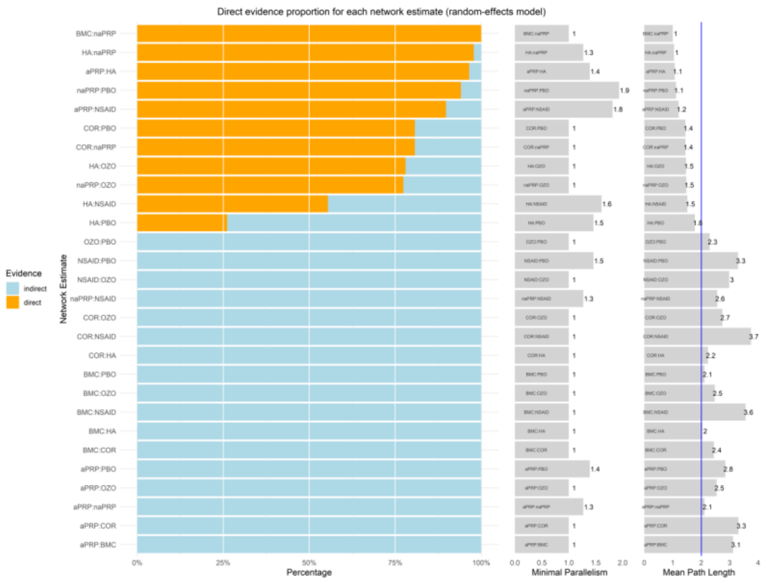

Figure S16. Direct evidence plot representing direct evidence proportion for each assessment corresponding to the network meta-analysis of the effect of PRP stratified by platelet activation status (activated PRP = aPRP, and non-activated PRP = naPRP) vs different non-surgical control treatments (ACE: Acetaminophen, BMC: Bone Marrow Concentrate, COR: Corticosteroids, HA: Hyaluronic Acid, NSAID: Non-steroidal Anti-Inflammatory Drugs, OZO: Ozone therapy, and PBO: Placebo) on WOMAC Total at the following time points: A) 6 months, and B) 12 months. Key geometry metrics depicted include minimal path length, representing the shortest distance between nodes, and mean path length, reflecting the shortest parallel connections between nodes, and mean path length, quantifying the average shortest path across all pairs of interventions within the network.

KOOS Pain

A)

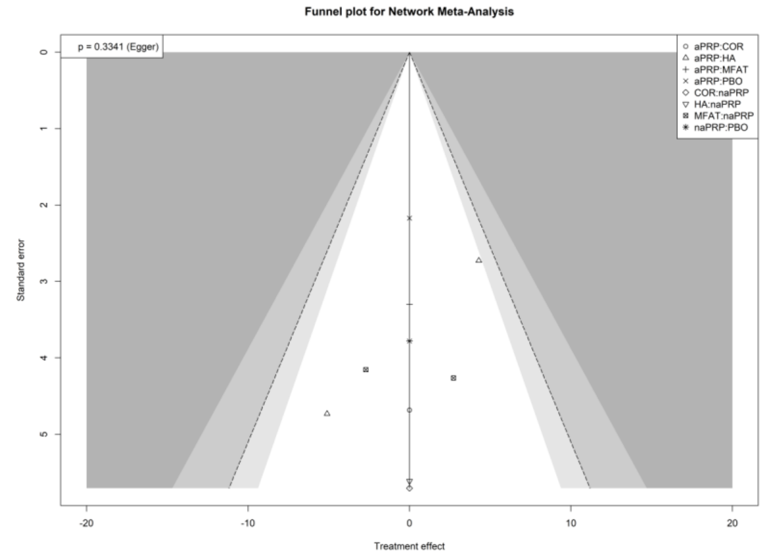

B)

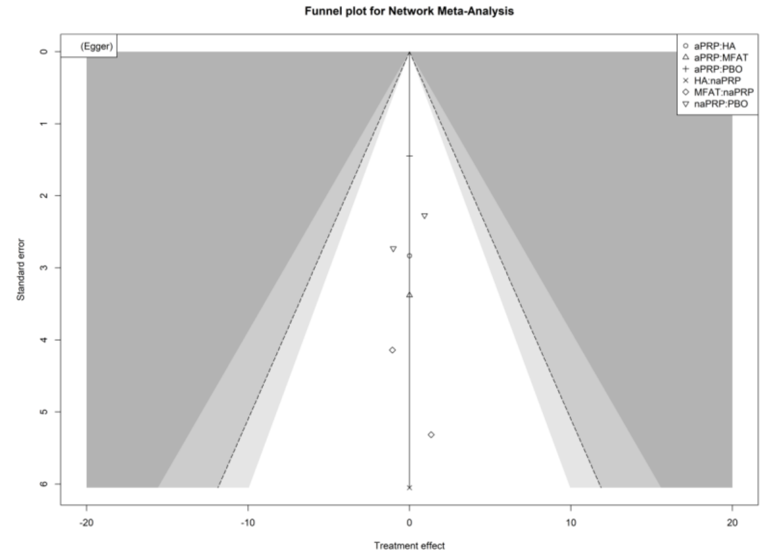

Figure S17. Contour enhanced funnel plot for trials of the effect of PRP stratified by platelet activation status (activated PRP = aPRP, and non-activated PRP = naPRP) vs different non-surgical control treatments (COR: Corticosteroids, HA: Hyaluronic Acid, MFAT: Microfragmented Adipose Tissue, and PBO: Placebo) on KOOS Pain at the following time points: A) 6 months, and B) 12 months. The vertical line represents the pooled effect estimate. Contour lines indicate regions of statistical significance (*p*<0.01, *p*<0.05, and *p*<0.10). Asymmetry in the distribution of studies may suggest potential publication bias or small-study effects.

A)

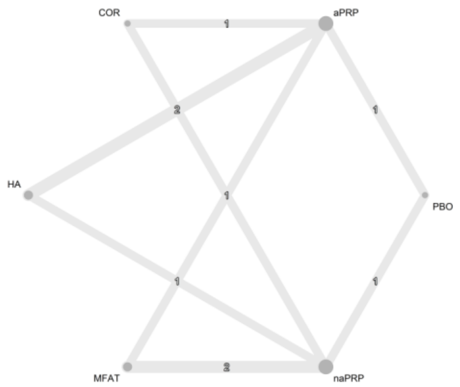

B)

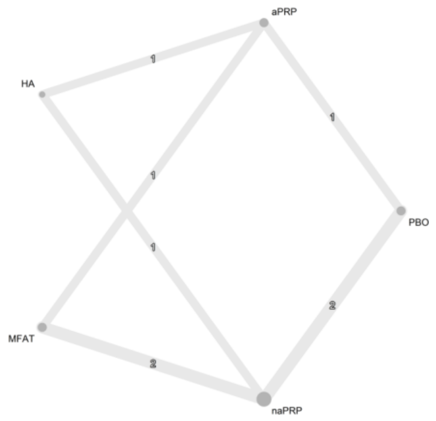

Figure S18. Network plot representing direct comparisons among interventions included in the network meta-analysis for KOOS Pain at the following time points: A) 6 months, and B) 12 months. PRP treatments were stratified by platelet activation status (activated PRP = aPRP, and non-activated PRP = naPRP) vs different non-surgical control treatments (COR: Corticosteroids, HA: Hyaluronic Acid, MFAT: Microfragmented Adipose Tissue, and PBO: Placebo). Node size is proportional to the total number of participants receiving each intervention, and edge thickness reflects the number of studies contributing to each direct comparison.

A)

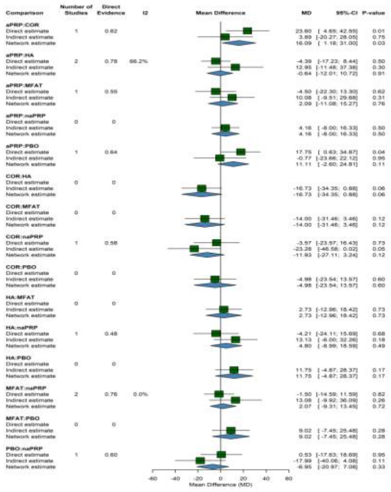

B)

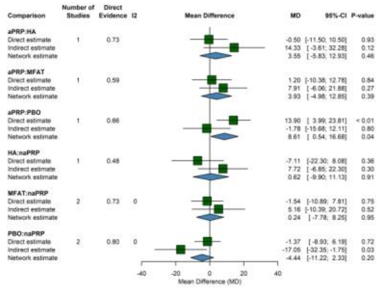

Figure S19. Node-split forest plot representing inconsistency assessment in the network meta-analysis of the effect of PRP stratified by platelet activation status (activated PRP = aPRP, and non-activated PRP = naPRP) vs different non-surgical control treatments (COR: Corticosteroids, HA: Hyaluronic Acid, MFAT: Microfragmented Adipose Tissue, and PBO: Placebo) on KOOS Pain at the following time points: A) 6 months, and B) 12 months. Each treatment comparison includes both direct and indirect estimates for mean difference (MD) with corresponding 95% confidence intervals.

A)

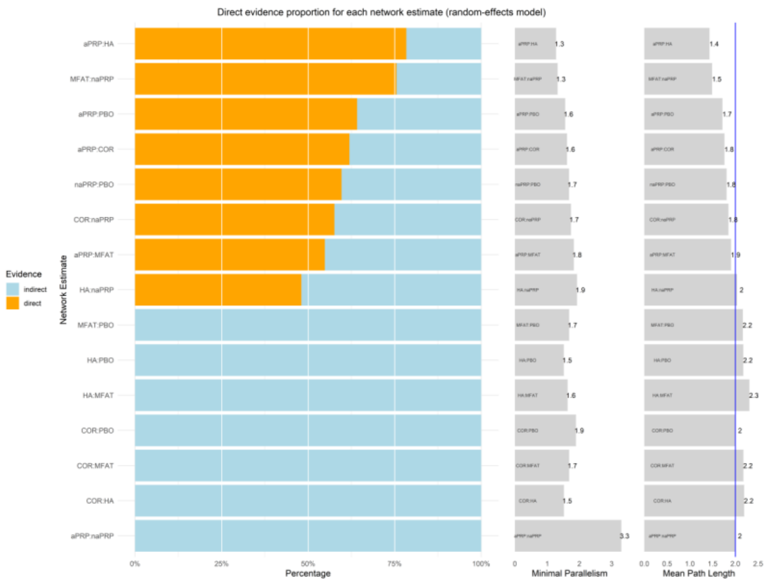

B)

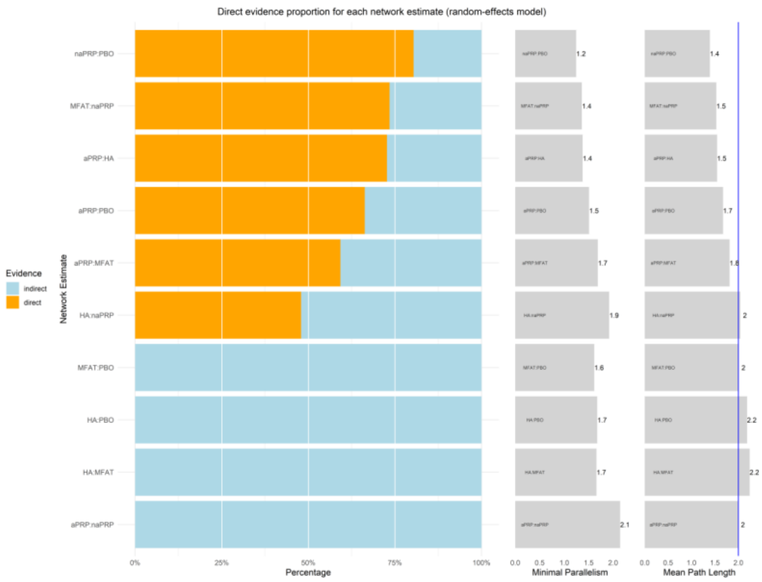

Figure S20. Direct evidence plot representing direct evidence proportion for each assessment corresponding to the network meta-analysis of the effect of PRP stratified by platelet activation status (activated PRP = aPRP, and non-activated PRP = naPRP) vs different non-surgical control treatments (COR: Corticosteroids, HA: Hyaluronic Acid, MFAT: Microfragmented Adipose Tissue, and PBO: Placebo) on KOOS Pain at the following time points: A) 6 months, and B) 12 months. Key geometry metrics depicted include minimal path length, representing the shortest distance between nodes, and mean path length, reflecting the shortest parallel connections between nodes, and mean path length, quantifying the average shortest path across all pairs of interventions within the network.

KOOS Activities of Daily Living

A)

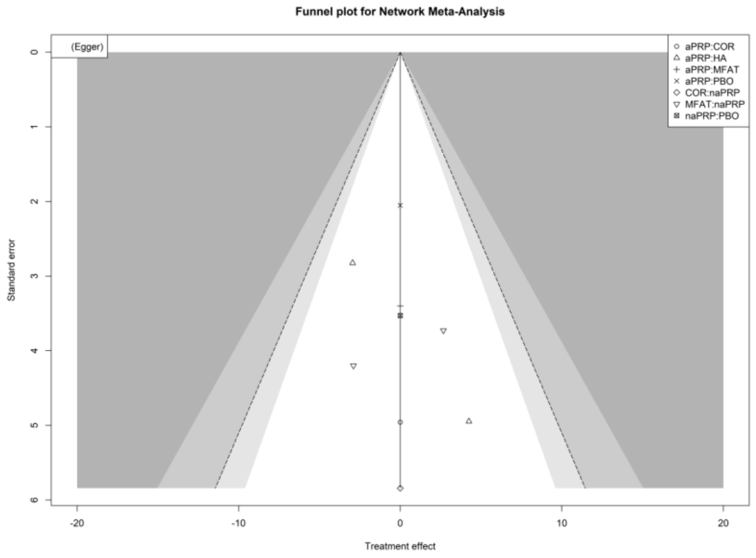

B)

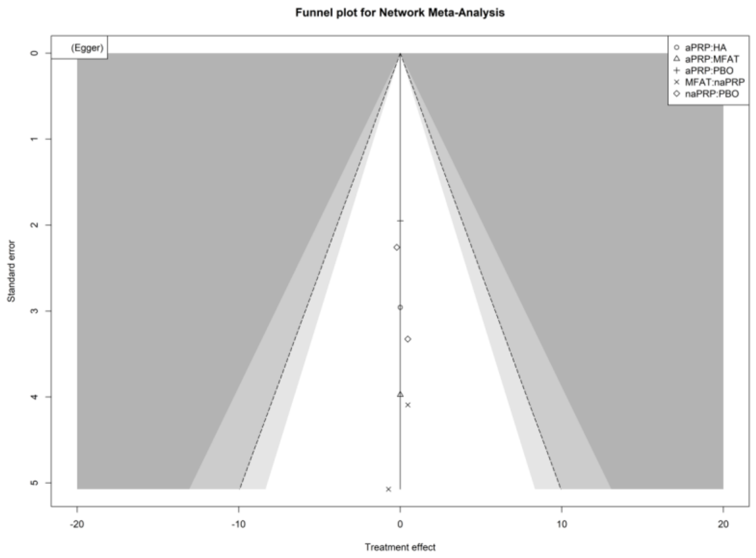

Figure S21. Contour enhanced funnel plot for trials of the effect of PRP stratified by platelet activation status (activated PRP = aPRP, and non-activated PRP = naPRP) vs different non-surgical control treatments (COR: Corticosteroids, HA: Hyaluronic Acid, MFAT: Microfragmented Adipose Tissue, and PBO: Placebo) on KOOS Activities of Daily Living at the following time points: A) 6 months, and B) 12 months. The vertical line represents the pooled effect estimate. Contour lines indicate regions of statistical significance (*p*<0.01, *p*<0.05, and *p*<0.10). Asymmetry in the distribution of studies may suggest potential publication bias or small-study effects.

A)

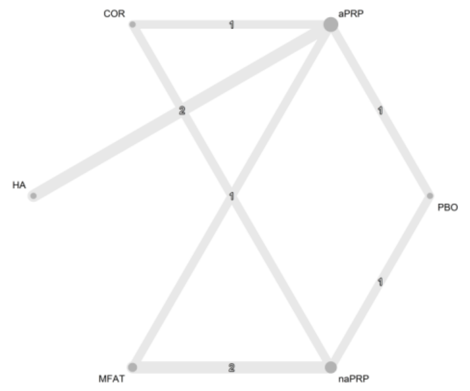

B)

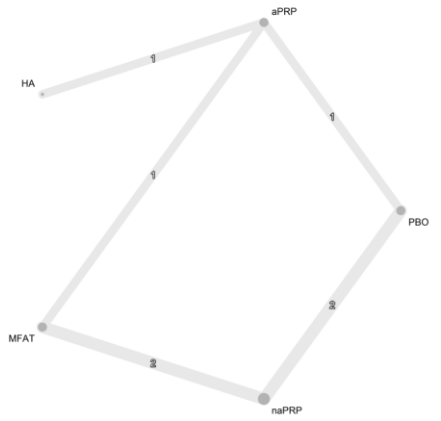

Figure S22. Network plot representing direct comparisons among interventions included in the network meta-analysis for KOOS Activities of Daily Living at the following time points: A) 6 months, and B) 12 months. PRP treatments were stratified by platelet activation status (activated PRP = aPRP, and non-activated PRP = naPRP) vs different non-surgical control treatments (COR: Corticosteroids, HA: Hyaluronic Acid, MFAT: Microfragmented Adipose Tissue, and PBO: Placebo). Node size is proportional to the total number of participants receiving each intervention, and edge thickness reflects the number of studies contributing to each direct comparison.

A)

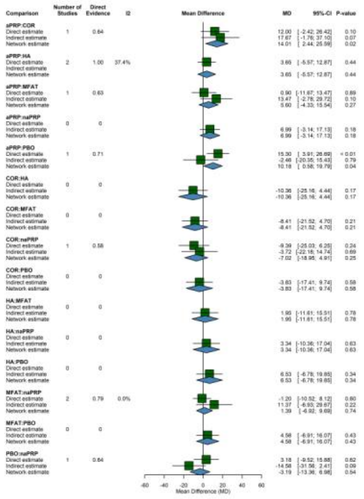

B)

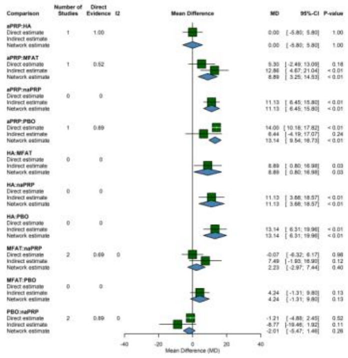

Figure S23. Node-split forest plot representing inconsistency assessment in the network meta-analysis of the effect of PRP stratified by platelet activation status (activated PRP = aPRP, and non-activated PRP = naPRP) vs different non-surgical control treatments (COR: Corticosteroids, HA: Hyaluronic Acid, MFAT: Microfragmented Adipose Tissue, and PBO: Placebo) on KOOS Activities of Daily Living at the following time points: A) 6 months, and B) 12 months. Each treatment comparison includes both direct and indirect estimates for mean difference (MD) with corresponding 95% confidence intervals.

A)

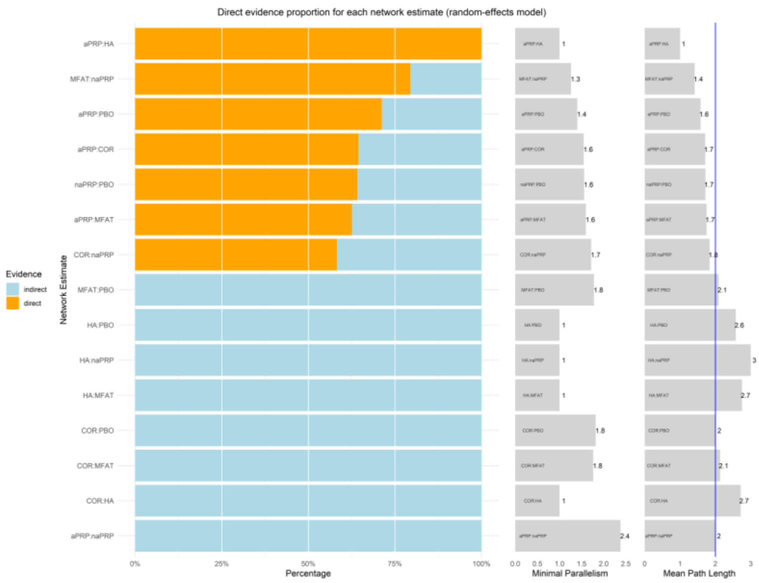

B)

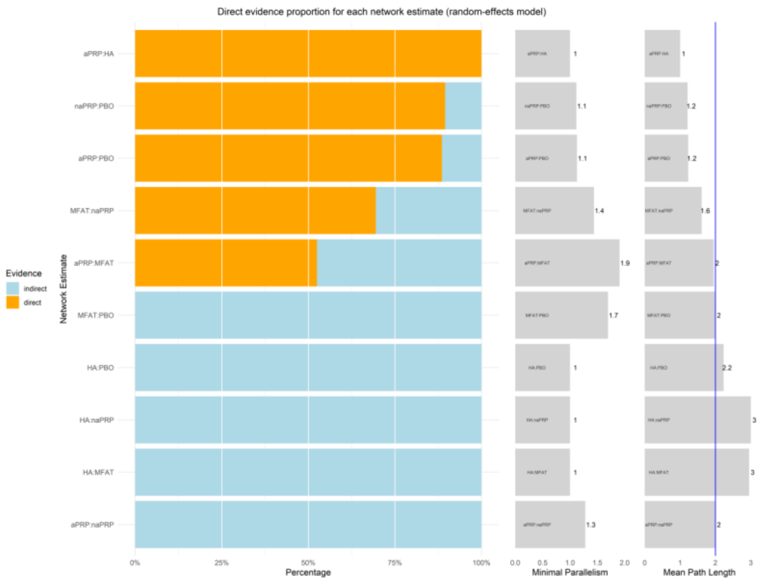

Figure S24. Direct evidence plot representing direct evidence proportion for each assessment corresponding to the network meta-analysis of the effect of PRP stratified by platelet activation status (activated PRP = aPRP, and non-activated PRP = naPRP) vs different non-surgical control treatments (COR: Corticosteroids, HA: Hyaluronic Acid, MFAT: Microfragmented Adipose Tissue, and PBO: Placebo) on KOOS Activities of Daily Living at the following time points: A) 6 months, and B) 12 months. Key geometry metrics depicted include minimal path length, representing the shortest distance between nodes, and mean path length, reflecting the shortest parallel connections between nodes, and mean path length, quantifying the average shortest path across all pairs of interventions within the network.

KOOS Sport and Recreation Function

A)

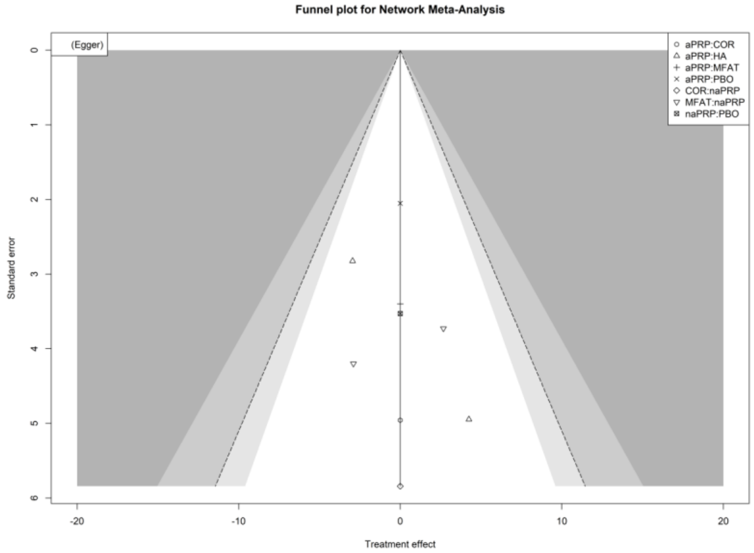

B)

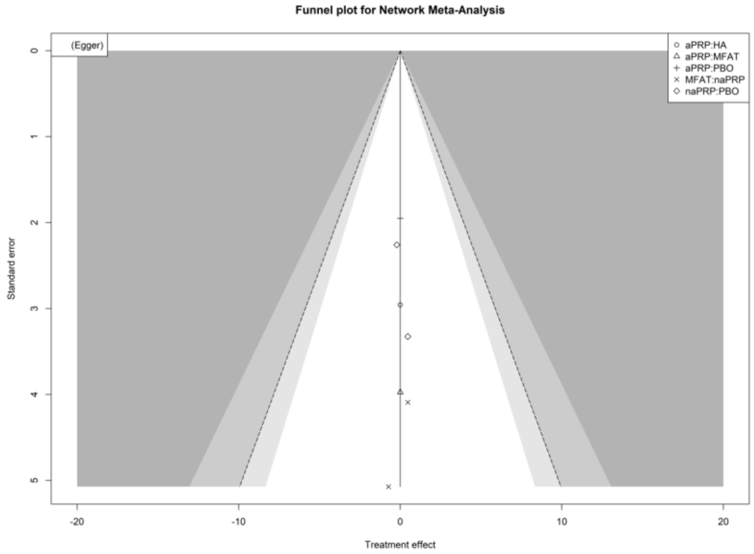

Figure S25. Contour enhanced funnel plot for trials of the effect of PRP stratified by platelet activation status (activated PRP = aPRP, and non-activated PRP = naPRP) vs different non-surgical control treatments (COR: Corticosteroids, HA: Hyaluronic Acid, MFAT: Microfragmented Adipose Tissue, and PBO: Placebo) on KOOS Sport and Recreation Function at the following time points: A) 6 months, and B) 12 months. The vertical line represents the pooled effect estimate. Contour lines indicate regions of statistical significance (*p*<0.01, *p*<0.05, and *p*<0.10). Asymmetry in the distribution of studies may suggest potential publication bias or small-study effects.

A)

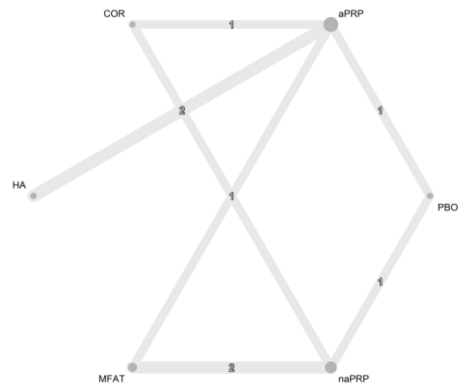

B)

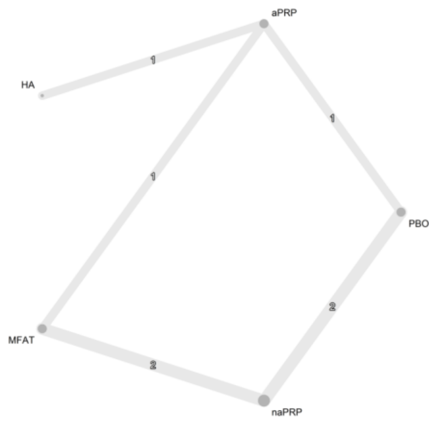

Figure S26. Network plot representing direct comparisons among interventions included in the network meta-analysis for KOOS Sport and Recreation Function at the following time points: A) 6 months, and B) 12 months. PRP treatments were stratified by platelet activation status (activated PRP = aPRP, and non-activated PRP = naPRP) vs different non-surgical control treatments (COR: Corticosteroids, HA: Hyaluronic Acid, MFAT: Microfragmented Adipose Tissue, and PBO: Placebo). Node size is proportional to the total number of participants receiving each intervention, and edge thickness reflects the number of studies contributing to each direct comparison.

A)

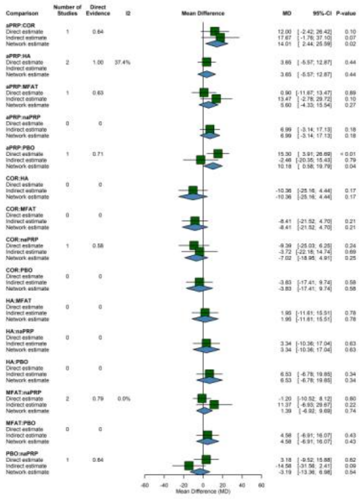

B)

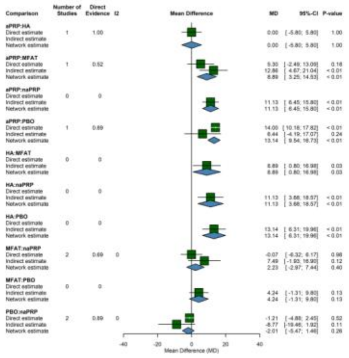

Figure S27. Node-split forest plot representing inconsistency assessment in the network meta-analysis of the effect of PRP stratified by platelet activation status (activated PRP = aPRP, and non-activated PRP = naPRP) vs different non-surgical control treatments (COR: Corticosteroids, HA: Hyaluronic Acid, MFAT: Microfragmented Adipose Tissue, and PBO: Placebo) on KOOS Sport and Recreation Function at the following time points: A) 6 months, and B) 12 months. Each treatment comparison includes both direct and indirect estimates for mean difference (MD) with corresponding 95% confidence intervals.

A)

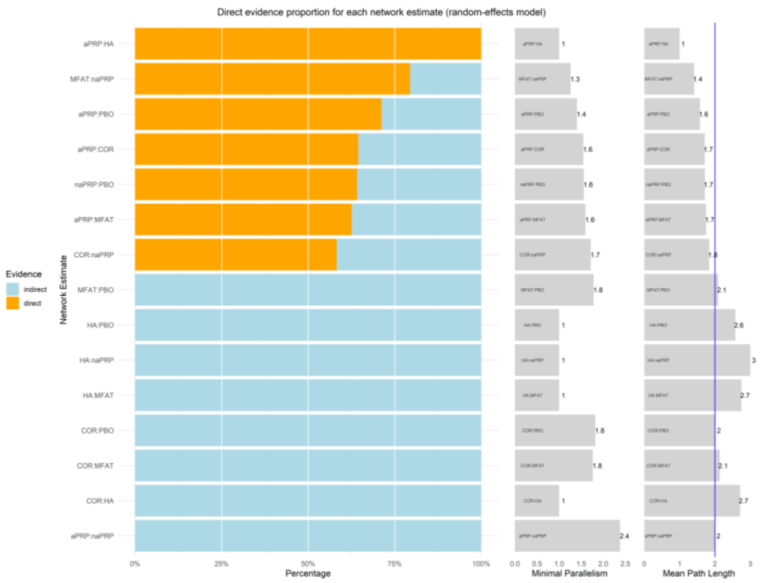

B)

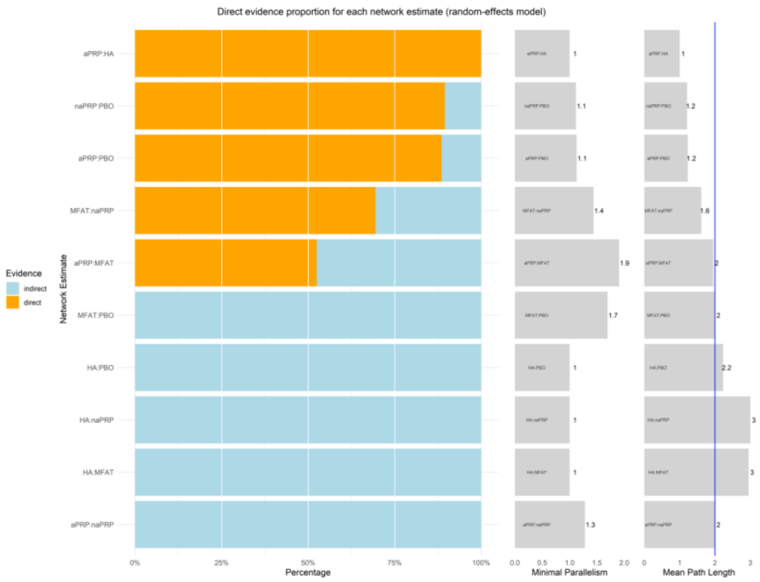

Figure S28. Direct evidence plot representing direct evidence proportion for each assessment corresponding to the network meta-analysis of the effect of PRP stratified by platelet activation status (activated PRP = aPRP, and non-activated PRP = naPRP) vs different non-surgical control treatments (COR: Corticosteroids, HA: Hyaluronic Acid, MFAT: Microfragmented Adipose Tissue, and PBO: Placebo) on KOOS Sport and Recreation Function at the following time points: A) 6 months, and B) 12 months. Key geometry metrics depicted include minimal path length, representing the shortest distance between nodes, and mean path length, reflecting the shortest parallel connections between nodes, and mean path length, quantifying the average shortest path across all pairs of interventions within the network.

KOOS Knee-Related Quality of Life

A)

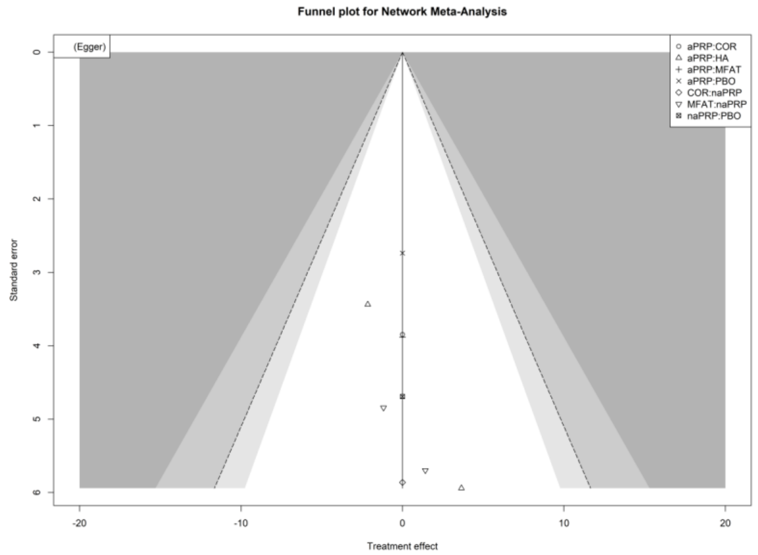

B)

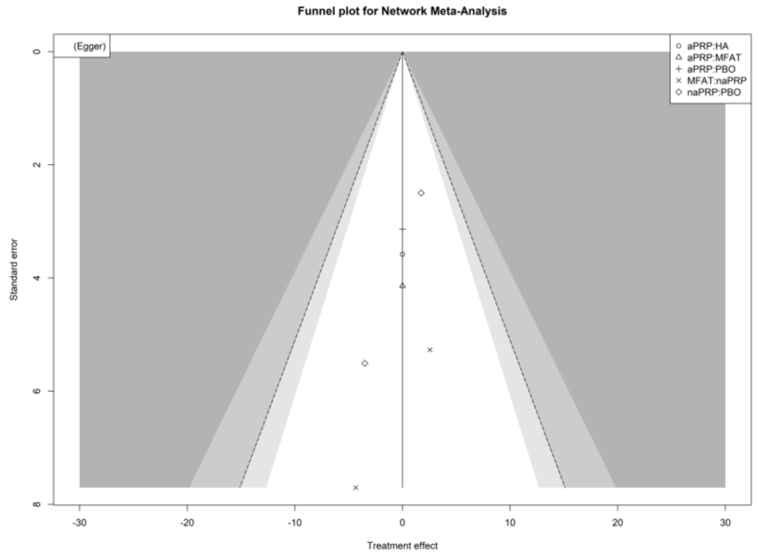

Figure S29. Contour enhanced funnel plot for trials of the effect of PRP stratified by platelet activation status (activated PRP = aPRP, and non-activated PRP = naPRP) vs different non-surgical control treatments (COR: Corticosteroids, HA: Hyaluronic Acid, MFAT: Microfragmented Adipose Tissue, and PBO: Placebo) on KOOS Knee-Related Quality of Life at the following time points: A) 6 months, and B) 12 months. The vertical line represents the pooled effect estimate. Contour lines indicate regions of statistical significance (*p*<0.01, *p*<0.05, and *p*<0.10). Asymmetry in the distribution of studies may suggest potential publication bias or small-study effects.

A)

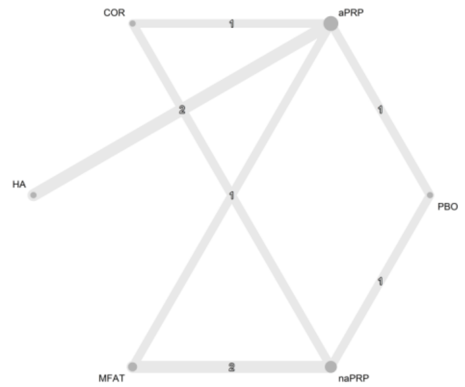

B)

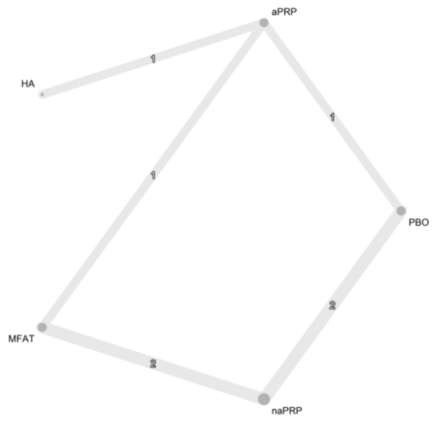

Figure S30. Network plot representing direct comparisons among interventions included in the network meta-analysis for KOOS Knee-Related Quality of Life at the following time points: A) 6 months, and B) 12 months. PRP treatments were stratified by platelet activation status (activated PRP = aPRP, and non-activated PRP = naPRP) vs different non-surgical control treatments (COR: Corticosteroids, HA: Hyaluronic Acid, MFAT: Microfragmented Adipose Tissue, and PBO: Placebo). Node size is proportional to the total number of participants receiving each intervention, and edge thickness reflects the number of studies contributing to each direct comparison.

A)

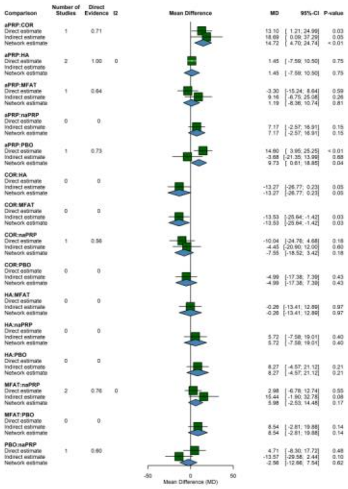

B)

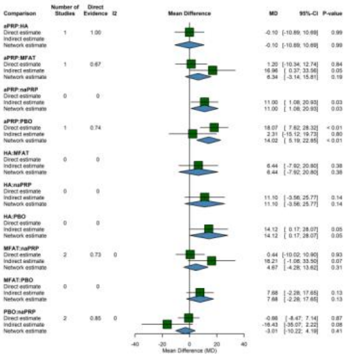

Figure S31. Node-split forest plot representing inconsistency assessment in the network meta-analysis of the effect of PRP stratified by platelet activation status (activated PRP = aPRP, and non-activated PRP = naPRP) vs different non-surgical control treatments (COR: Corticosteroids, HA: Hyaluronic Acid, MFAT: Microfragmented Adipose Tissue, and PBO: Placebo) on KOOS Knee-Related Quality of Life at the following time points: A) 6 months, and B) 12 months. Each treatment comparison includes both direct and indirect estimates for mean difference (MD) with corresponding 95% confidence intervals.

A)

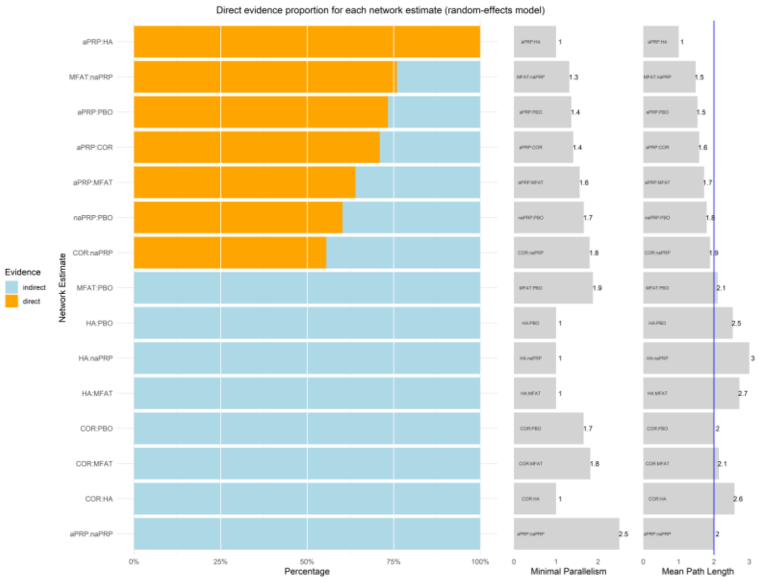

B)

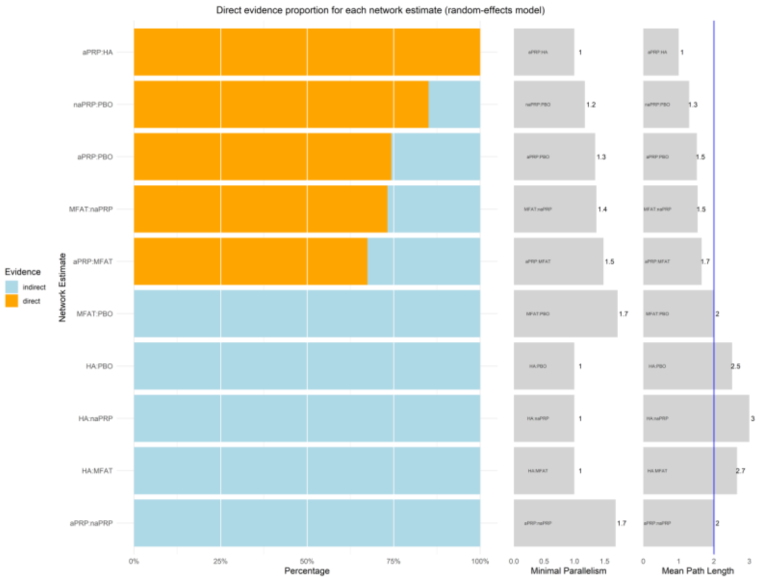

Figure S32. Direct evidence plot representing direct evidence proportion for each assessment corresponding to the network meta-analysis of the effect of PRP stratified by platelet activation status (activated PRP = aPRP, and non-activated PRP = naPRP) vs different non-surgical control treatments (COR: Corticosteroids, HA: Hyaluronic Acid, MFAT: Microfragmented Adipose Tissue, and PBO: Placebo) on KOOS Knee-Related Quality of Life at the following time points: A) 6 months, and B) 12 months. Key geometry metrics depicted include minimal path length, representing the shortest distance between nodes, and mean path length, reflecting the shortest parallel connections between nodes, and mean path length, quantifying the average shortest path across all pairs of interventions within the network.

KOOS Symptoms

A)

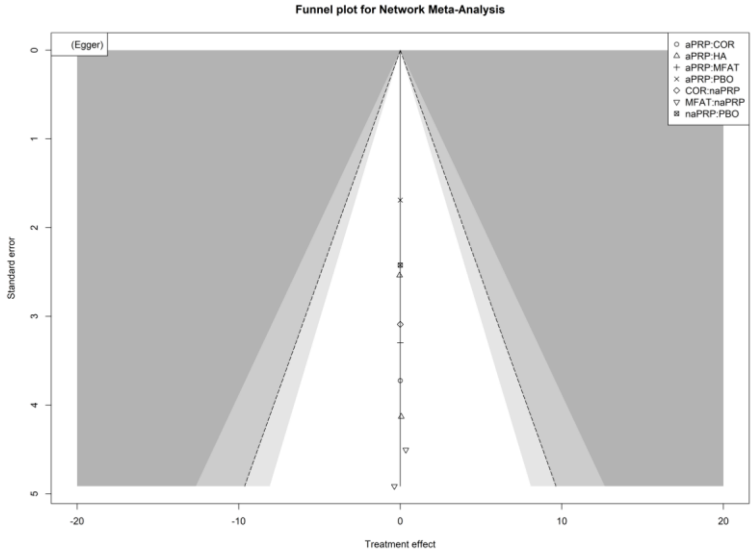

B)

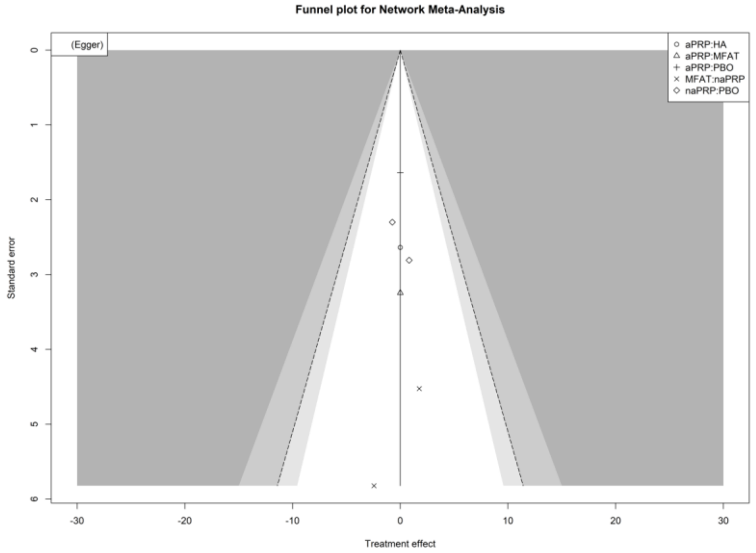

Figure S33. Contour enhanced funnel plot for trials of the effect of PRP stratified by platelet activation status (activated PRP = aPRP, and non-activated PRP = naPRP) vs different non-surgical control treatments (COR: Corticosteroids, HA: Hyaluronic Acid, MFAT: Microfragmented Adipose Tissue, and PBO: Placebo) on KOOS Symptoms at the following time points: A) 6 months, and B) 12 months. The vertical line represents the pooled effect estimate. Contour lines indicate regions of statistical significance (p<0.01, p<0.05, and p<0.10). Asymmetry in the distribution of studies may suggest potential publication bias or small-study effects.

A)

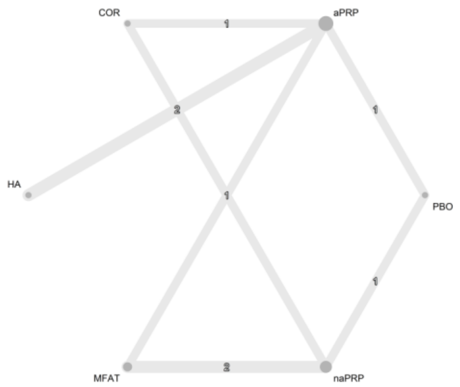

B)

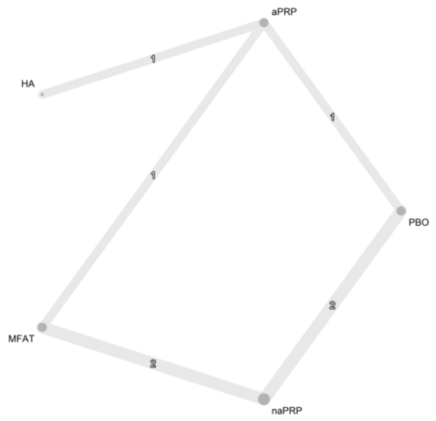

Figure S34. Network plot representing direct comparisons among interventions included in the network meta-analysis for KOOS Symptoms at the following time points: A) 6 months, and B) 12 months. PRP treatments were stratified by platelet activation status (activated PRP = aPRP, and non-activated PRP = naPRP) vs different non-surgical control treatments (COR: Corticosteroids, HA: Hyaluronic Acid, MFAT: Microfragmented Adipose Tissue, and PBO: Placebo). Node size is proportional to the total number of participants receiving each intervention, and edge thickness reflects the number of studies contributing to each direct comparison.

A)

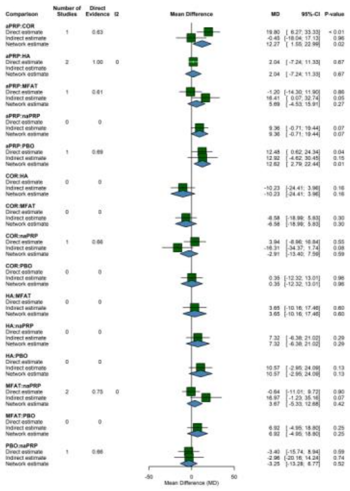

B)

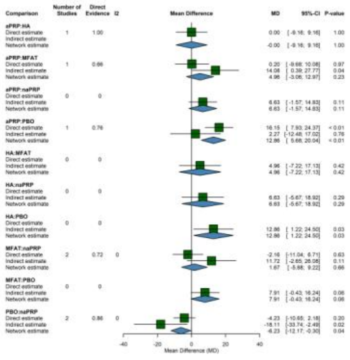

Figure S35. Node-split forest plot representing inconsistency assessment in the network meta-analysis of the effect of PRP stratified by platelet activation status (activated PRP = aPRP, and non-activated PRP = naPRP) vs different non-surgical control treatments (COR: Corticosteroids, HA: Hyaluronic Acid, MFAT: Microfragmented Adipose Tissue, and PBO: Placebo) on KOOS Symptoms at the following time points: A) 6 months, and B) 12 months. Each treatment comparison includes both direct and indirect estimates for mean difference (MD) with corresponding 95% confidence intervals.

A)

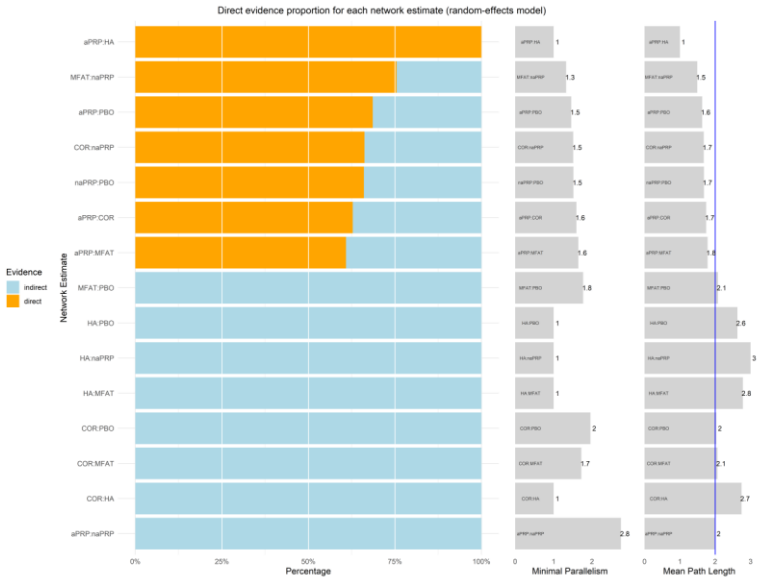

B)

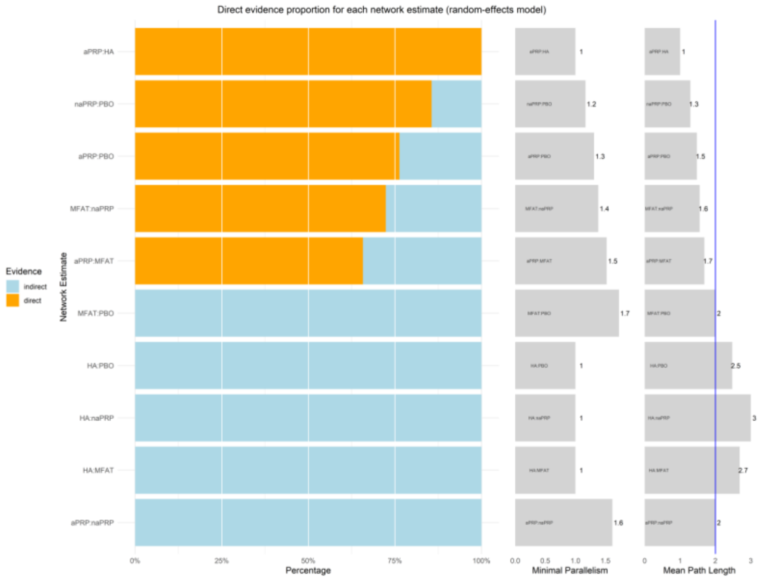

Figure S36. Direct evidence plot representing direct evidence proportion for each assessment corresponding to the network meta-analysis of the effect of PRP stratified by platelet activation status (activated PRP = aPRP, and non-activated PRP = naPRP) vs different non-surgical control treatments (ACE: Acetaminophen, BMC: Bone Marrow Concentrate, COR: Corticosteroids, HA: Hyaluronic Acid, NSAID: Non-steroidal Anti-Inflammatory Drugs, and OZO: Ozone therapy) on KOOS Symptoms at the following time points: A) 6 months, and B) 12 months. Key geometry metrics depicted include minimal path length, representing the shortest distance between nodes, and mean path length, reflecting the shortest parallel connections between nodes, and mean path length, quantifying the average shortest path across all pairs of interventions within the network.

VAS

A)

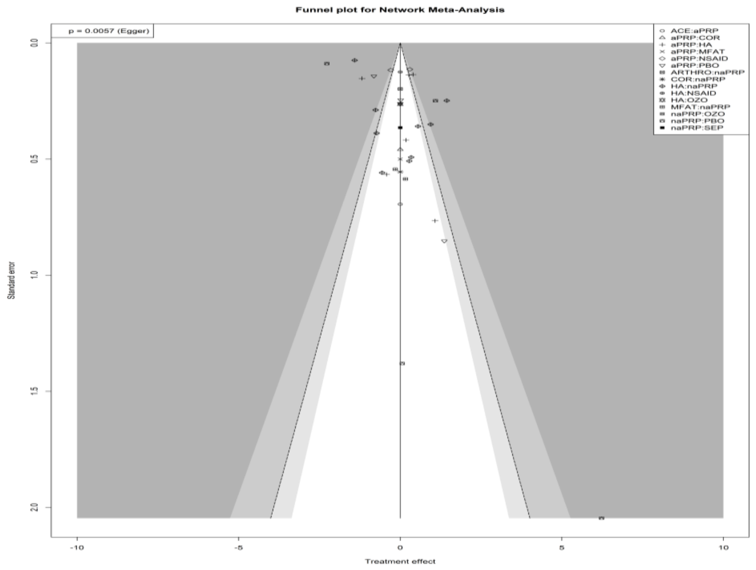

B)

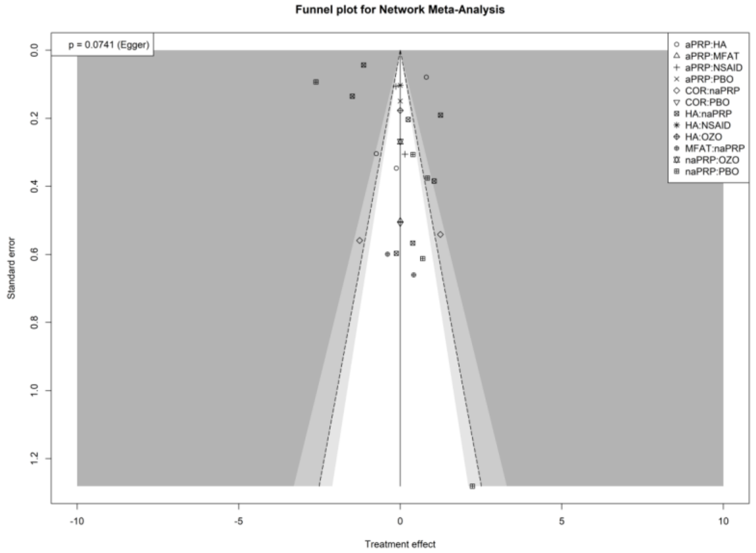

Figure S37. Contour enhanced funnel plot for trials of the effect of PRP stratified by platelet activation status (activated PRP = aPRP, and non-activated PRP = naPRP) vs different non-surgical control treatments (ACE: Acetaminophen, ARTHRO: Arthroscopy, BMC: Bone Marrow Concentrate, COR: Corticosteroids, HA: Hyaluronic Acid, MFAT: Microfragmented Adipose Tissue, NSAID: Non-steroidal Anti-Inflammatory Drugs, OZO: Ozone therapy, PBO: Placebo, and SEP: Structured Exercise Program) on VAS at the following time points: A) 6 months, and B) 12 months. The vertical line represents the pooled effect estimate. Contour lines indicate regions of statistical significance (*p*<0.01, *p*<0.05, and *p*<0.10). Asymmetry in the distribution of studies may suggest potential publication bias or small-study effects.

A)

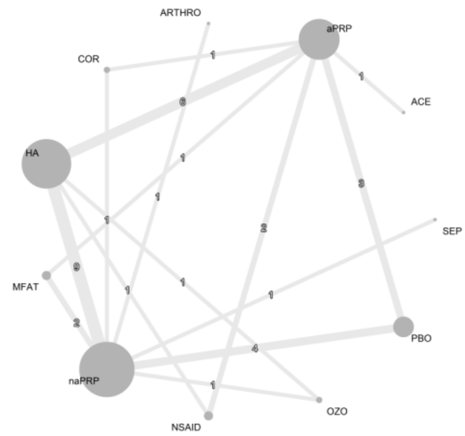

B)

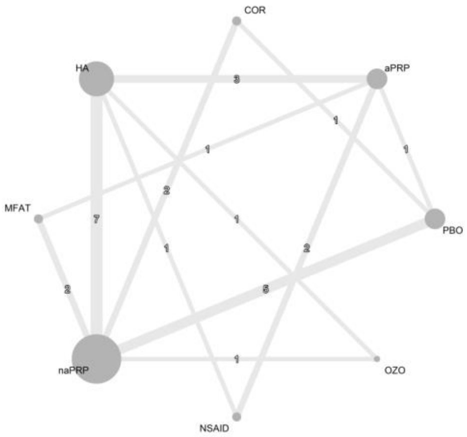

Figure S38. Network plot representing direct comparisons among interventions included in the network meta-analysis for VAS at the following time points: A) 6 months, and B) 12 months. PRP treatments were stratified by platelet activation status (activated PRP = aPRP, and non-activated PRP = naPRP) vs different non-surgical control treatments (ACE: Acetaminophen, ARTHRO: Arthroscopy, BMC: Bone Marrow Concentrate, COR: Corticosteroids, HA: Hyaluronic Acid, MFAT: Microfragmented Adipose Tissue, NSAID: Non-steroidal Anti-Inflammatory Drugs, OZO: Ozone therapy, PBO: Placebo, and SEP: Structured Exercise Program). Node size is proportional to the total number of participants receiving each intervention, and edge thickness reflects the number of studies contributing to each direct comparison.

A)

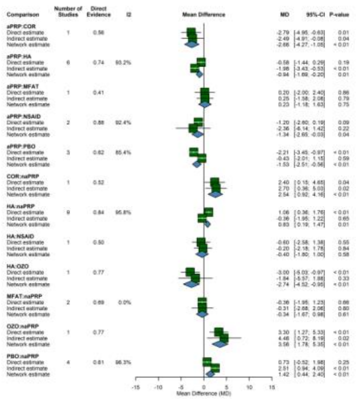

B)

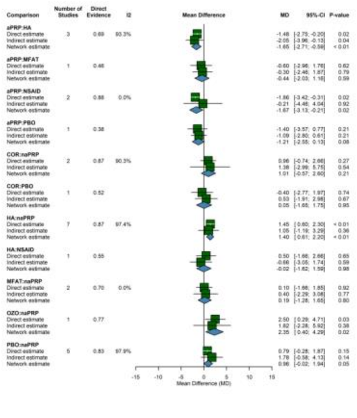

Figure S39. Node-split forest plot representing inconsistency assessment in the network meta-analysis of the effect of PRP stratified by platelet activation status (activated PRP = aPRP, and non-activated PRP = naPRP) vs different non-surgical control treatments (ACE: Acetaminophen, ARTHRO: Arthroscopy, BMC: Bone Marrow Concentrate, COR: Corticosteroids, HA: Hyaluronic Acid, MFAT: Microfragmented Adipose Tissue, NSAID: Non-steroidal Anti-Inflammatory Drugs, OZO: Ozone therapy, PBO: Placebo, and SEP: Structured Exercise Program) on VAS at the following time points: A) 6 months, and B) 12 months. Each treatment comparison includes both direct and indirect estimates for mean difference (MD) with corresponding 95% confidence intervals.

A)

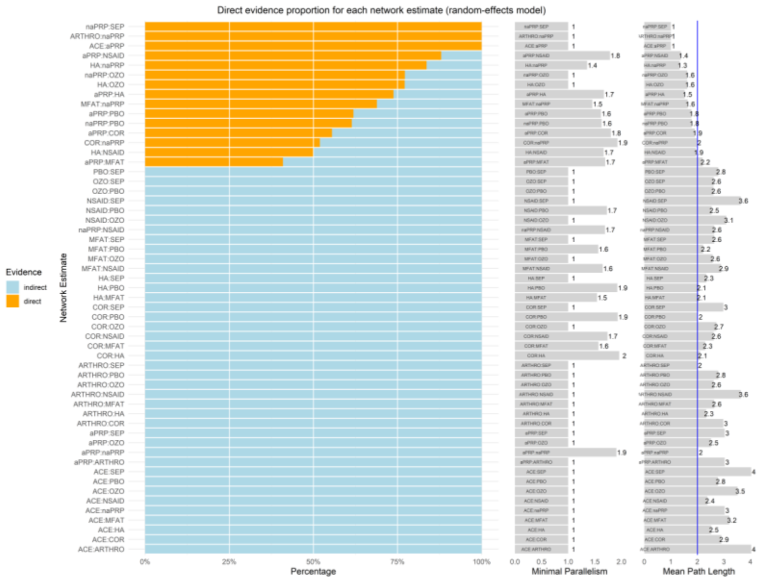

B)

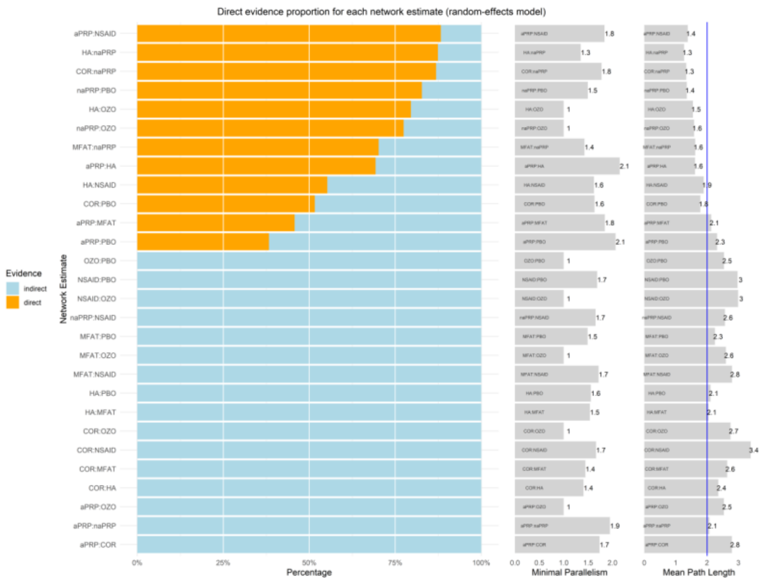

Figure S40. Direct evidence plot representing direct evidence proportion for each assessment corresponding to the network meta-analysis of the effect of PRP stratified by platelet activation status (activated PRP = aPRP, and non-activated PRP = naPRP) vs different non-surgical control treatments (ACE: Acetaminophen, ARTHRO: Arthroscopy, BMC: Bone Marrow Concentrate, COR: Corticosteroids, HA: Hyaluronic Acid, MFAT: Microfragmented Adipose Tissue, NSAID: Non-steroidal Anti-Inflammatory Drugs, OZO: Ozone therapy, PBO: Placebo, and SEP: Structured Exercise Program) on VAS at the following time points: A) 6 months, and B) 12 months. Key geometry metrics depicted include minimal path length, representing the shortest distance between nodes, and mean path length, reflecting the shortest parallel connections between nodes, and mean path length, quantifying the average shortest path across all pairs of interventions within the network.

IKDC

A)

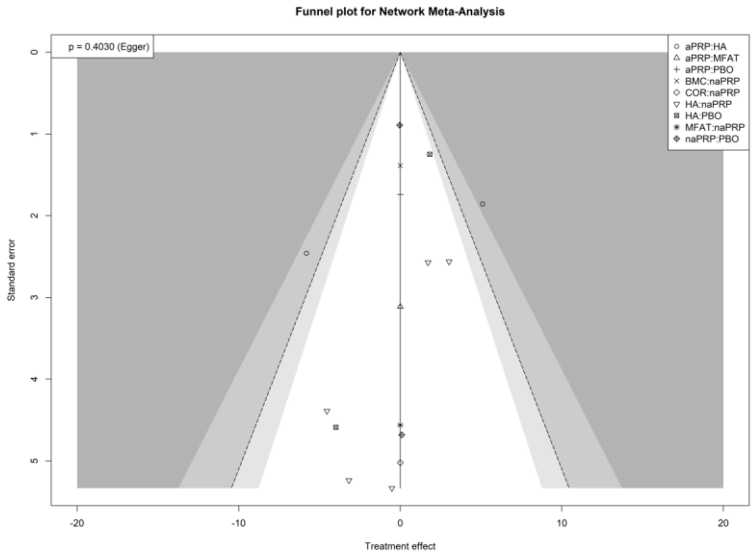

B)

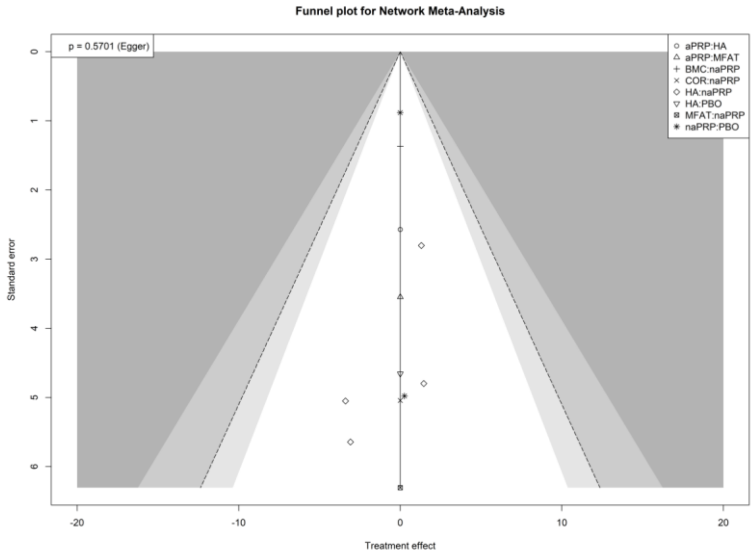

Figure S41. Contour enhanced funnel plot for trials of the effect of PRP stratified by platelet activation status (activated PRP = aPRP, and non-activated PRP = naPRP) vs different non-surgical control treatments (BMC: Bone Marrow Concentrate, COR: Corticosteroids, HA: Hyaluronic Acid, MFAT: Microfragmented Adipose Tissue, PBO: Placebo, and SEP: Structured Exercise Program) on IKDC at the following time points: A) 6 months, and B) 12 months. The vertical line represents the pooled effect estimate. Contour lines indicate regions of statistical significance (*p*<0.01, *p*<0.05, and *p*<0.10). Asymmetry in the distribution of studies may suggest potential publication bias or small-study effects.

A)

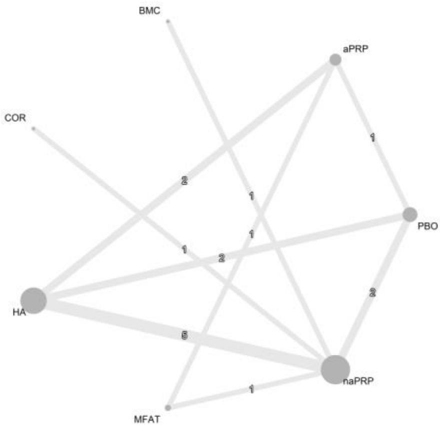

B)

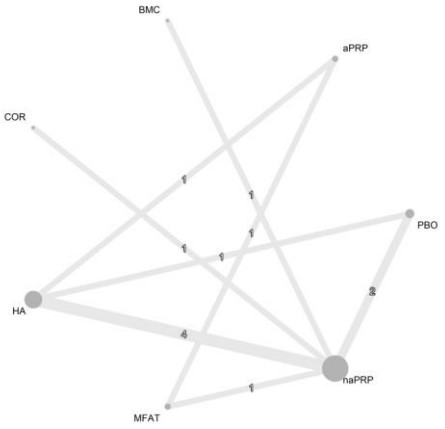

Figure S42. Network plot representing direct comparisons among interventions included in the network meta-analysis for IKDC at the following time points: A) 6 months, and B) 12 months. PRP treatments were stratified by platelet activation status (activated PRP = aPRP, and non-activated PRP = naPRP) vs different non-surgical control treatments (BMC: Bone Marrow Concentrate, COR: Corticosteroids, HA: Hyaluronic Acid, MFAT: Microfragmented Adipose Tissue, PBO: Placebo, and SEP: Structured Exercise Program). Node size is proportional to the total number of participants receiving each intervention, and edge thickness reflects the number of studies contributing to each direct comparison.

A)

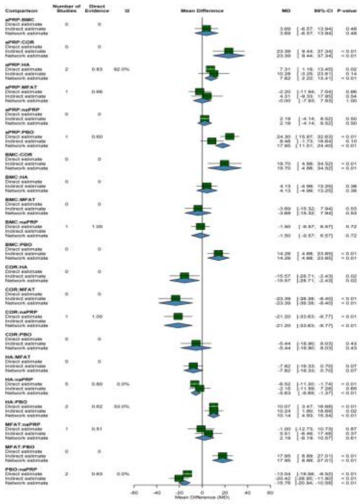

B)

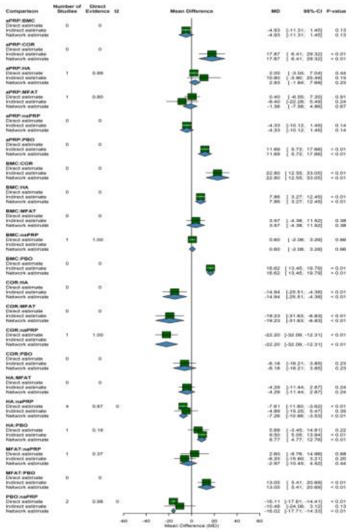

Figure S43. Node-split forest plot representing inconsistency assessment in the network meta-analysis of the effect of PRP stratified by platelet activation status (activated PRP = aPRP, and non-activated PRP = naPRP) vs different non-surgical control treatments (BMC: Bone Marrow Concentrate, COR: Corticosteroids, HA: Hyaluronic Acid, MFAT: Microfragmented Adipose Tissue, PBO: Placebo, and SEP: Structured Exercise Program) on IKDC at the following time points: A) 6 months, and B) 12 months. Each treatment comparison includes both direct and indirect estimates for mean difference (MD) with corresponding 95% confidence intervals.

A)

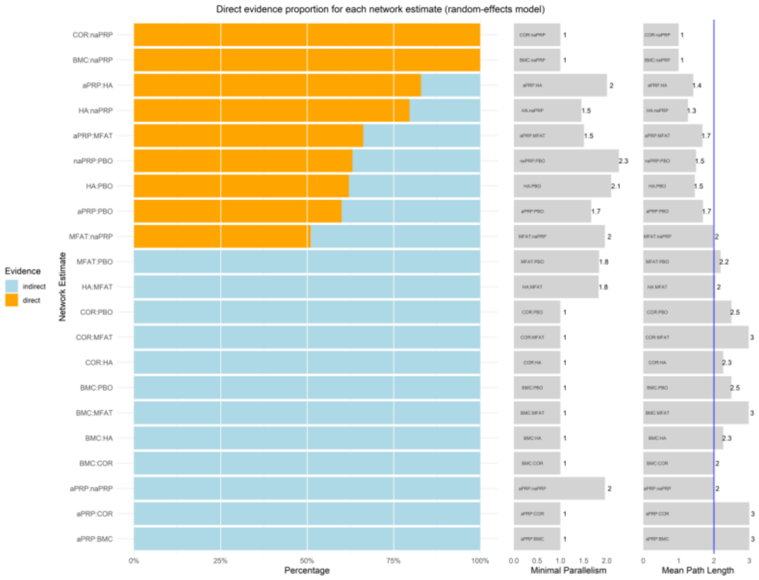

B)

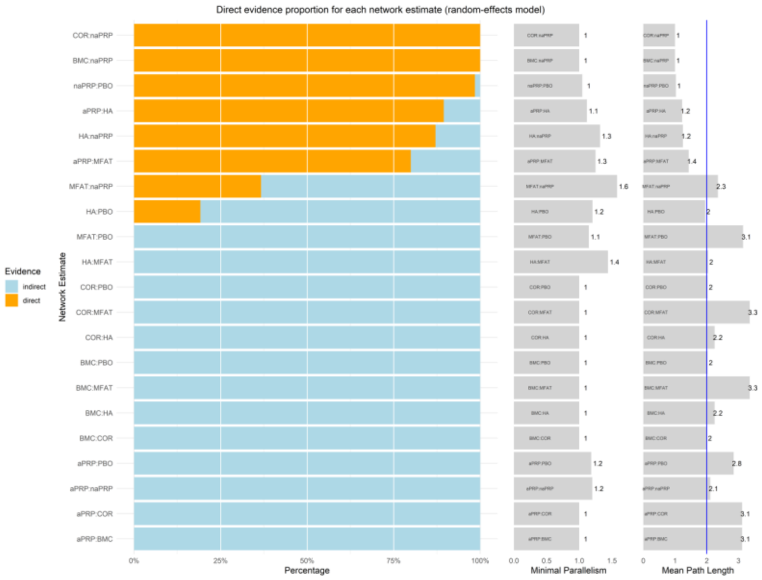

Figure S44. Direct evidence plot representing direct evidence proportion for each assessment corresponding to the network meta-analysis of the effect of PRP stratified by platelet activation status (activated PRP = aPRP, and non-activated PRP = naPRP) vs different non-surgical control treatments (BMC: Bone Marrow Concentrate, COR: Corticosteroids, HA: Hyaluronic Acid, MFAT: Microfragmented Adipose Tissue, PBO: Placebo, and SEP: Structured Exercise Program) on IKDC at the following time points: A) 6 months, and B) 12 months. Key geometry metrics depicted include minimal path length, representing the shortest distance between nodes, and mean path length, reflecting the shortest parallel connections between nodes, and mean path length, quantifying the average shortest path across all pairs of interventions within the network.

Lequesne Index

A)

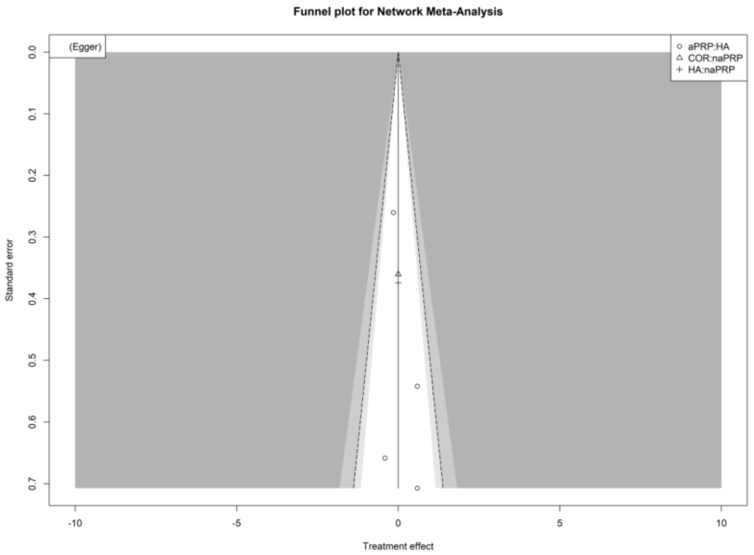

B)

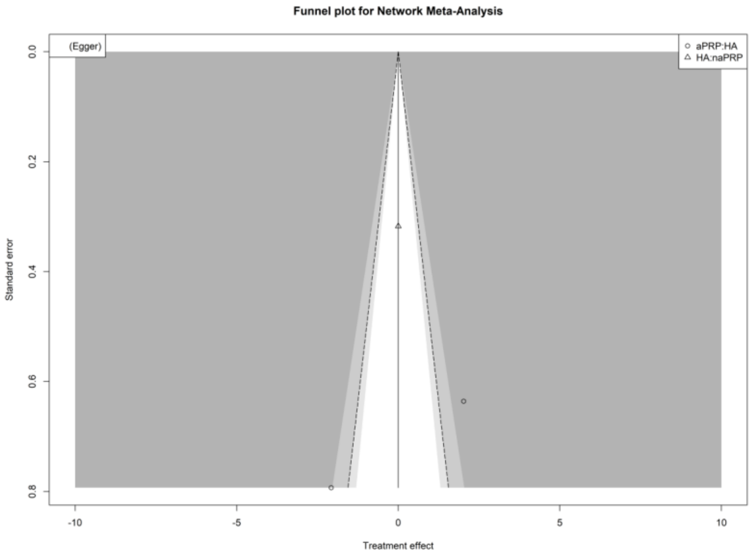

Figure S45. Contour enhanced funnel plot for trials of the effect of PRP stratified by platelet activation status (activated PRP = aPRP, and non-activated PRP = naPRP) vs different non-surgical control treatments (COR: Corticosteroids, and HA: Hyaluronic Acid) on Lequesne Index at the following time points: A) 6 months, and B) 12 months. The vertical line represents the pooled effect estimate. Contour lines indicate regions of statistical significance (*p*<0.01, *p*<0.05, and *p*<0.10). Asymmetry in the distribution of studies may suggest potential publication bias or small-study effects.

A)

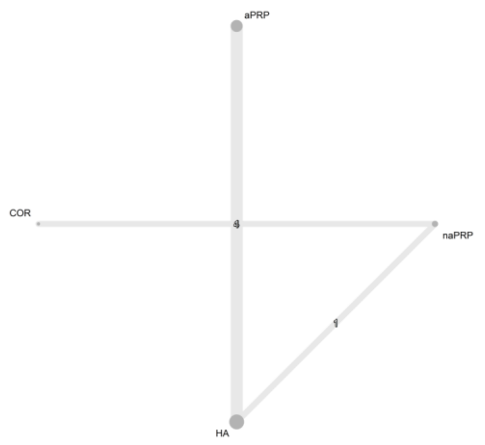

B)

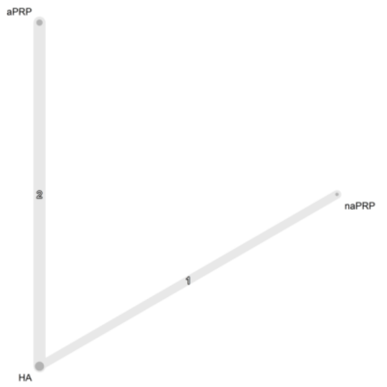

Figure S46. Network plot representing direct comparisons among interventions included in the network meta-analysis for Lequesne Index at the following time points: A) 6 months, and B) 12 months. PRP treatments were stratified by platelet activation status (activated PRP = aPRP, and non-activated PRP = naPRP) vs different non-surgical control treatments (COR: Corticosteroids, and HA: Hyaluronic Acid). Node size is proportional to the total number of participants receiving each intervention, and edge thickness reflects the number of studies contributing to each direct comparison.

A)

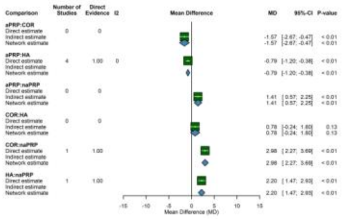

B)

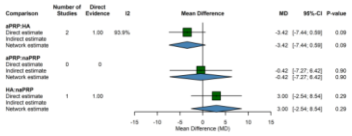

Figure S47. Node-split forest plot representing inconsistency assessment in the network meta-analysis of the effect of PRP stratified by platelet activation status (activated PRP = aPRP, and non-activated PRP = naPRP) vs different non-surgical control treatments (COR: Corticosteroids, and HA: Hyaluronic Acid) on Lequesne Index at the following time points: A) 6 months, and B) 12 months. Each treatment comparison includes both direct and indirect estimates for mean difference (MD) with corresponding 95% confidence intervals.

A)

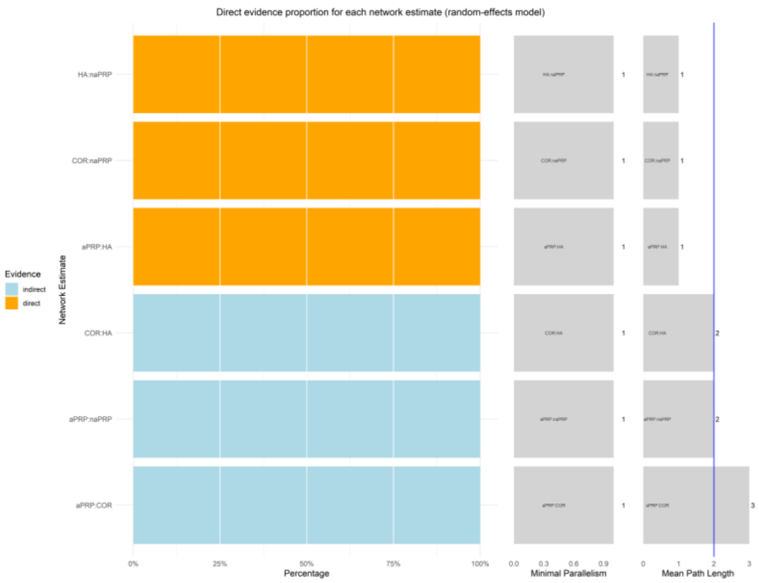

B)

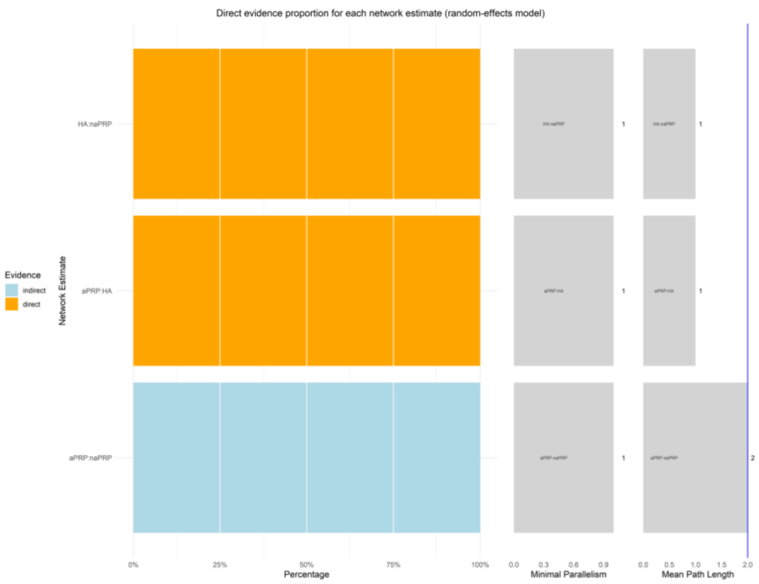

Figure S48. Direct evidence plot representing direct evidence proportion for each assessment corresponding to the network meta-analysis of the effect of PRP stratified by platelet activation status (activated PRP = aPRP, and non-activated PRP = naPRP) vs different non-surgical control treatments (COR: Corticosteroids, and HA: Hyaluronic Acid) on Lequesne Index at the following time points: A) 6 months, and B) 12 months. Key geometry metrics depicted include minimal path length, representing the shortest distance between nodes, and mean path length, reflecting the shortest parallel connections between nodes, and mean path length, quantifying the average shortest path across all pairs of interventions within the network.

Effect of PRP categories according to Mishra's classification system

WOMAC Pain

A)

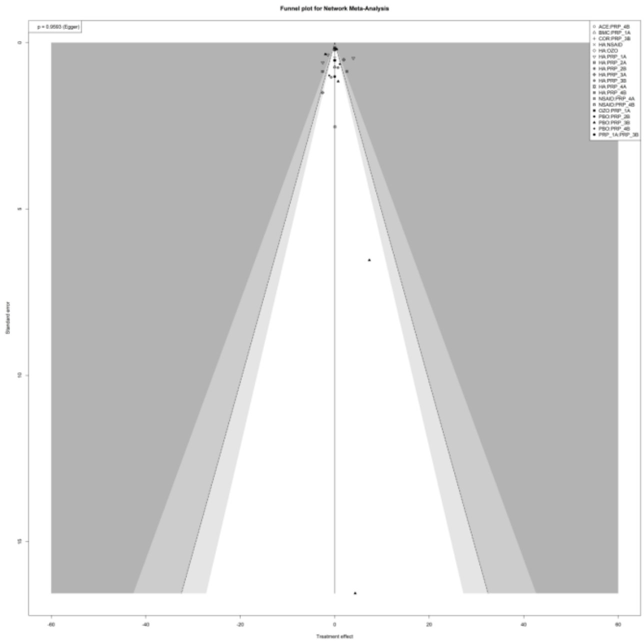

B)

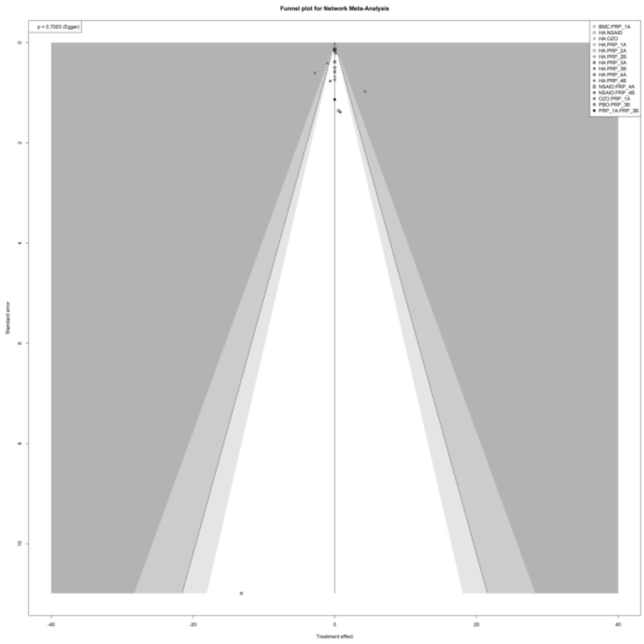

Figure S49. Contour enhanced funnel plot for trials of the effect of PRP categorized according to Mishra's classification system vs different non-surgical control treatments (ACE: Acetaminophen, BMC: Bone Marrow Concentrate, COR: Corticosteroids, HA: Hyaluronic Acid, NSAID: Non-steroidal Anti-Inflammatory Drugs, and OZO: Ozone therapy) on WOMAC Pain at the following time points: A) 6 months, and B) 12 months. The vertical line represents the pooled effect estimate. Contour lines indicate regions of statistical significance (*p*<0.01, *p*<0.05, and *p*<0.10). Asymmetry in the distribution of studies may suggest potential publication bias or small-study effects.

A)

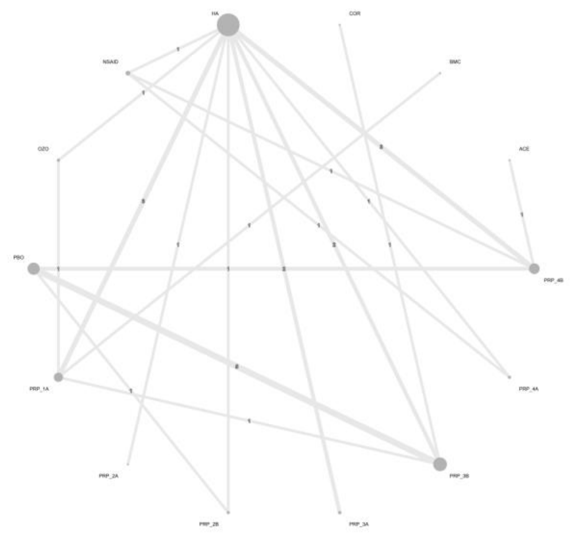

B)

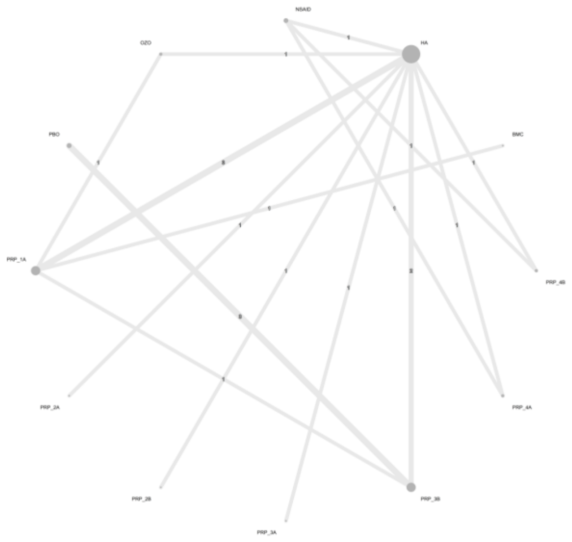

Figure S50. Network plot representing direct comparisons among interventions included in the network meta-analysis for WOMAC Pain at the following time points: A) 6 months, and B) 12 months. PRP treatments were categorized according to Mishra's classification system vs different non-surgical control treatments (ACE: Acetaminophen, BMC: Bone Marrow Concentrate, COR: Corticosteroids, HA: Hyaluronic Acid, NSAID: Non-steroidal Anti-Inflammatory Drugs, and OZO: Ozone therapy). Node size is proportional to the total number of participants receiving each intervention, and edge thickness reflects the number of studies contributing to each direct comparison.

A)

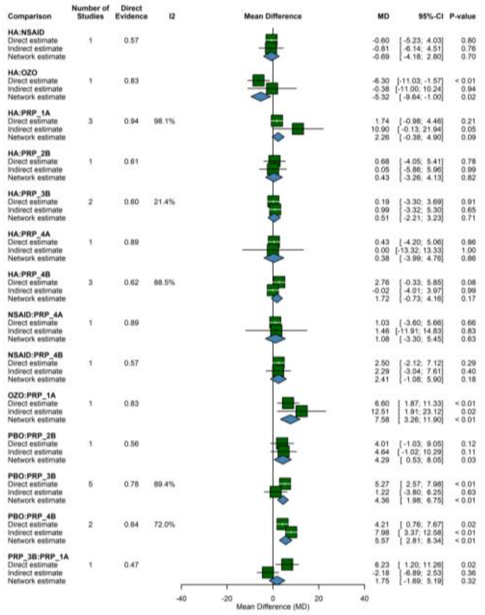

B)

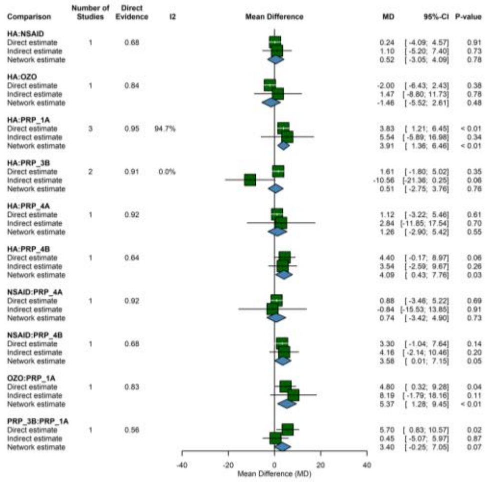

Figure S51. Node-split forest plot representing inconsistency assessment in the network meta-analysis of the effect of PRP categorized according to Mishra's classification system vs different non-surgical control treatments (ACE: Acetaminophen, BMC: Bone Marrow Concentrate, COR: Corticosteroids, HA: Hyaluronic Acid, NSAID: Non-steroidal Anti-Inflammatory Drugs, and OZO: Ozone therapy) on WOMAC Pain at the following time points: A) 6 months, and B) 12 months. Each treatment comparison includes both direct and indirect estimates for mean difference (MD) with corresponding 95% confidence intervals.

A)

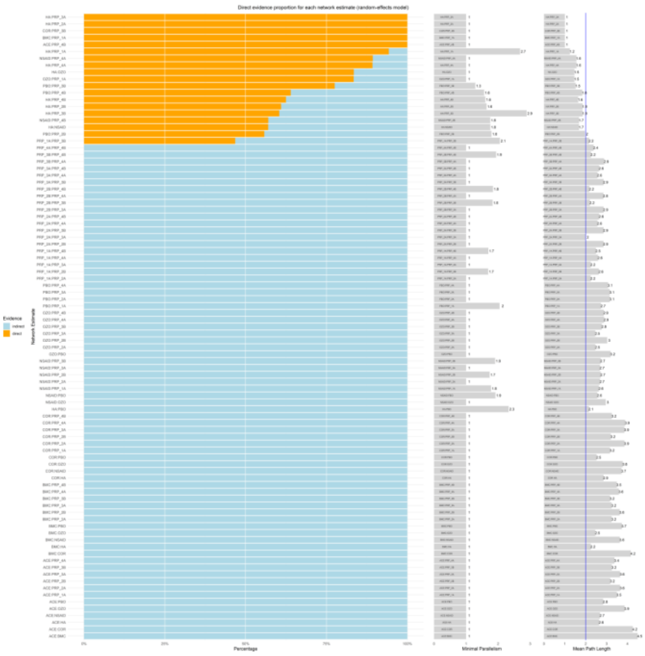

B)

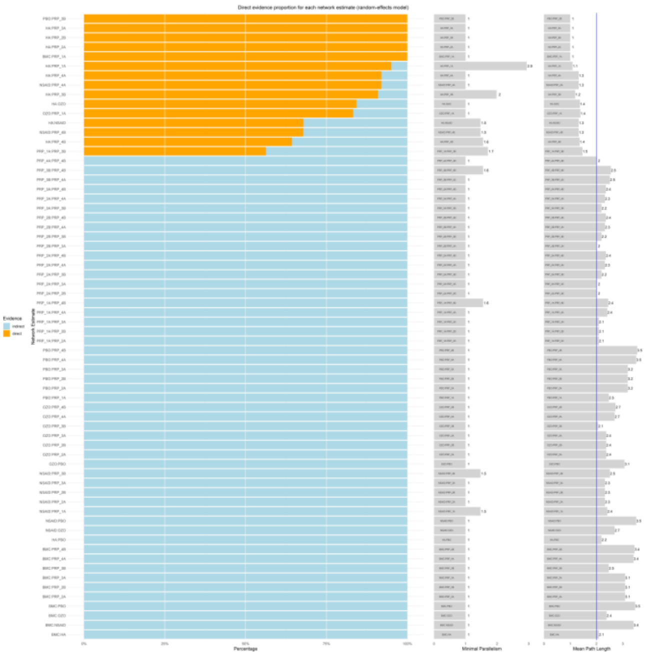

Figure S52. Direct evidence plot representing direct evidence proportion for each assessment corresponding to the network meta-analysis of the effect of PRP categorized according to Mishra's classification system vs different non-surgical control treatments (ACE: Acetaminophen, BMC: Bone Marrow Concentrate, COR: Corticosteroids, HA: Hyaluronic Acid, NSAID: Non-steroidal Anti-Inflammatory Drugs, and OZO: Ozone therapy) on WOMAC Pain at the following time points: A) 6 months, and B) 12 months. Key geometry metrics depicted include minimal path length, representing the shortest distance between nodes, and mean path length, reflecting the shortest parallel connections between nodes, and mean path length, quantifying the average shortest path across all pairs of interventions within the network.

WOMAC Stiffness

A)

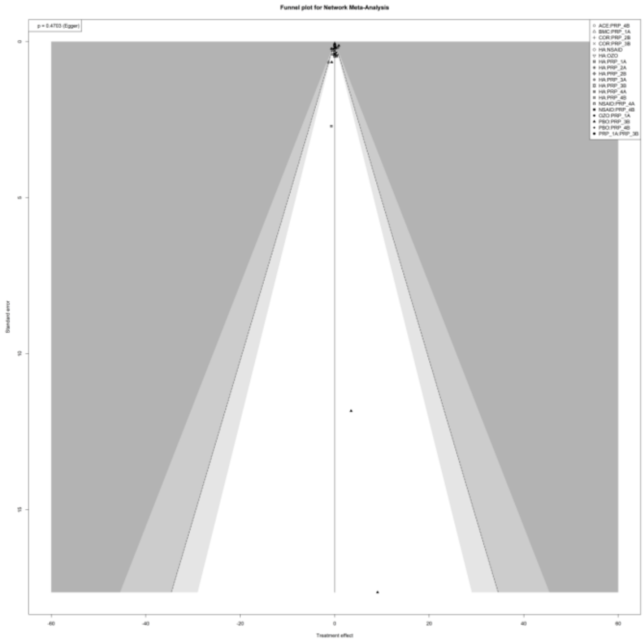

B)

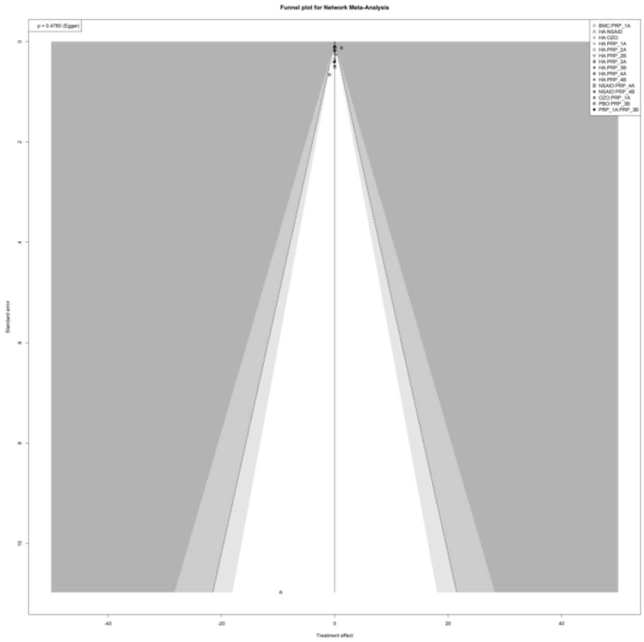

Figure S53. Contour enhanced funnel plot for trials of the effect of PRP categorized according to Mishra's classification system vs different non-surgical control treatments (ACE: Acetaminophen, BMC: Bone Marrow Concentrate, COR: Corticosteroids, HA: Hyaluronic Acid, NSAID: Non-steroidal Anti-Inflammatory Drugs, and OZO: Ozone therapy) on WOMAC Stiffness at the following time points: A) 6 months, and B) 12 months. The vertical line represents the pooled effect estimate. Contour lines indicate regions of statistical significance (*p*<0.01, *p*<0.05, and *p*<0.10). Asymmetry in the distribution of studies may suggest potential publication bias or small-study effects.

A)

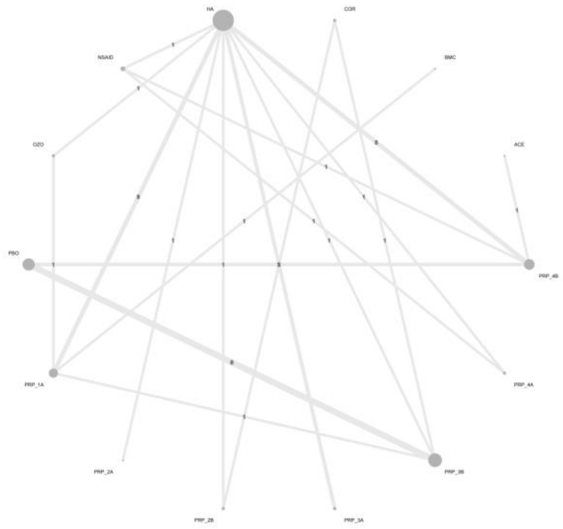

B)

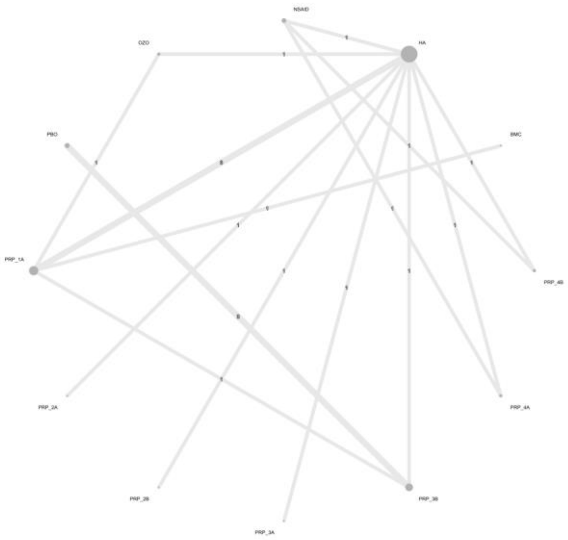

Figure S54. Network plot representing direct comparisons among interventions included in the network meta-analysis for WOMAC Stiffness at the following time points: A) 6 months, and B) 12 months. PRP treatments were categorized according to Mishra's classification system vs different non-surgical control treatments (ACE: Acetaminophen, BMC: Bone Marrow Concentrate, COR: Corticosteroids, HA: Hyaluronic Acid, NSAID: Non-steroidal Anti-Inflammatory Drugs, OZO: Ozone therapy, and PBO: Placebo). Node size is proportional to the total number of participants receiving each intervention, and edge thickness reflects the number of studies contributing to each direct comparison.

A)

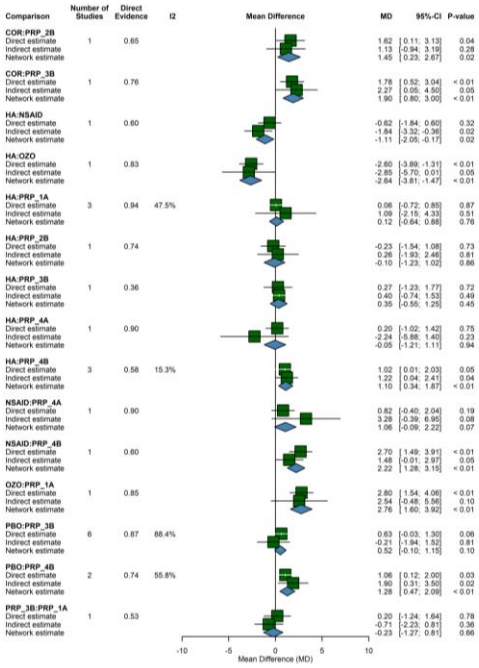

B)

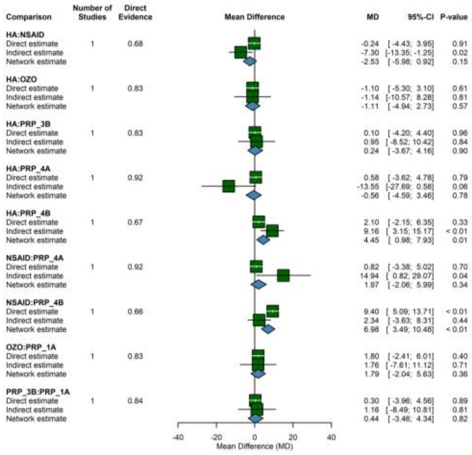

Figure S55. Node-split forest plot representing inconsistency assessment in the network meta-analysis of the effect of PRP categorized according to Mishra's classification system vs different non-surgical control treatments (ACE: Acetaminophen, BMC: Bone Marrow Concentrate, COR: Corticosteroids, HA: Hyaluronic Acid, NSAID: Non-steroidal Anti-Inflammatory Drugs, OZO: Ozone therapy, and PBO: Placebo) on WOMAC Stiffness at the following time points: A) 6 months, and B) 12 months. Each treatment comparison includes both direct and indirect estimates for mean difference (MD) with corresponding 95% confidence intervals.

A)

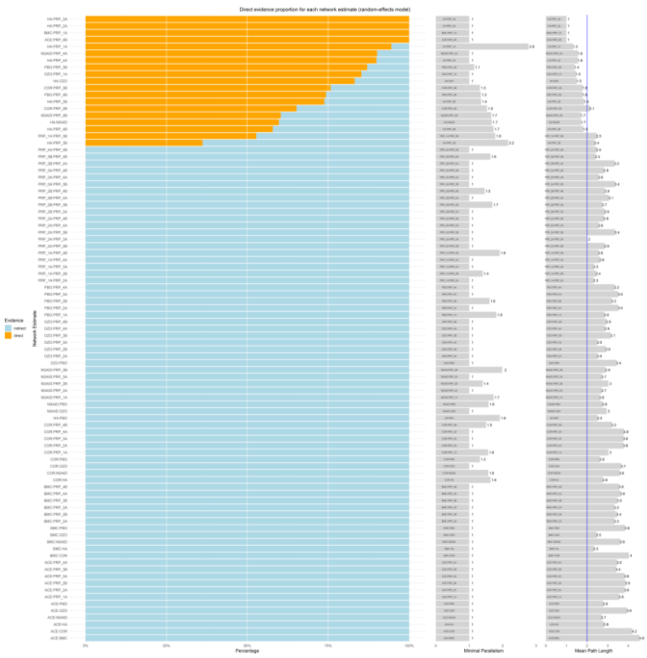

B)

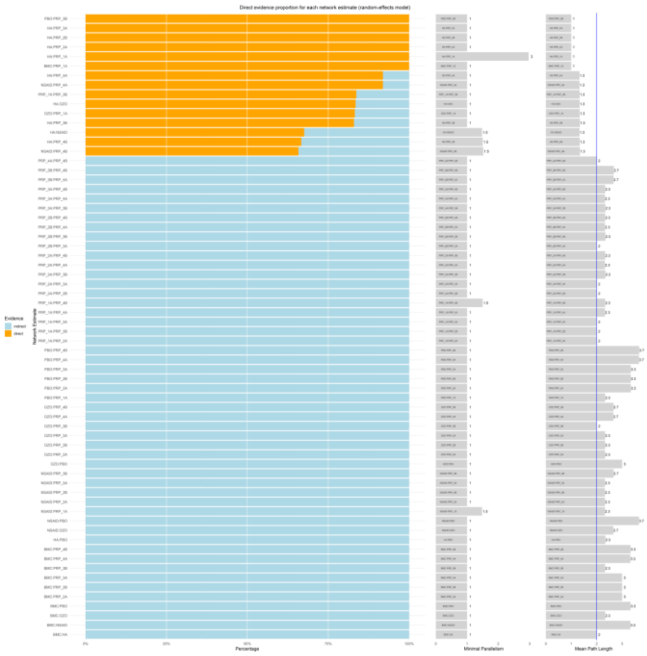

Figure S56. Direct evidence plot representing direct evidence proportion for each assessment corresponding to the network meta-analysis of the effect of PRP categorized according to Mishra’s classification system vs different non-surgical control treatments (ACE: Acetaminophen, BMC: Bone Marrow Concentrate, COR: Corticosteroids, HA: Hyaluronic Acid, NSAID: Non-steroidal Anti-Inflammatory Drugs, OZO: Ozone therapy, and PBO: Placebo) on WOMAC Stiffness at the following time points: A) 6 months, and B) 12 months. Key geometry metrics depicted include minimal path length, representing the shortest distance between nodes, and mean path length, reflecting the shortest parallel connections between nodes, and mean path length, quantifying the average shortest path across all pairs of interventions within the network.

WOMAC Physical Function

A)

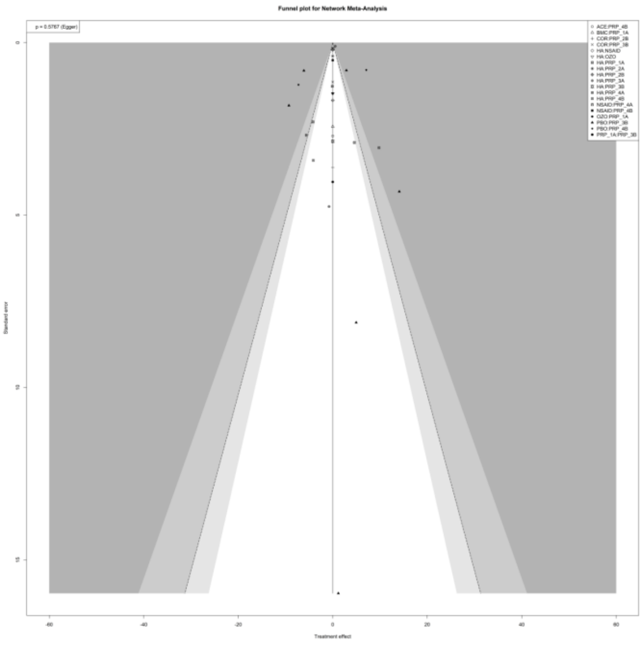

B)

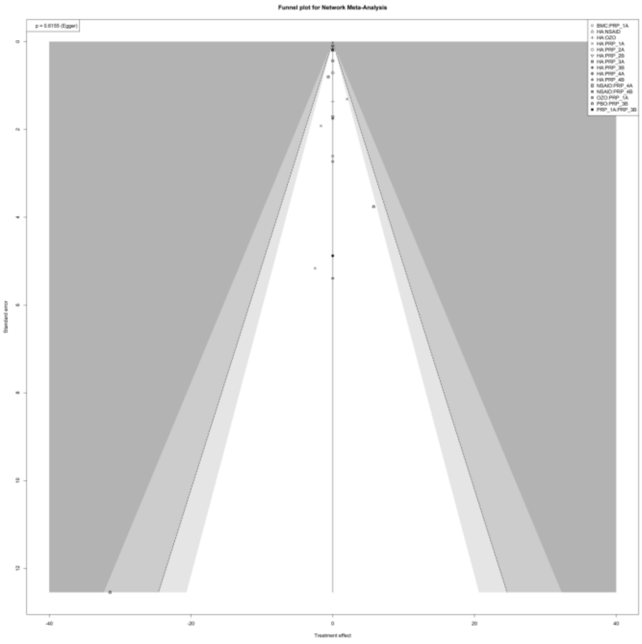

Figure S57. Contour enhanced funnel plot for trials of the effect of PRP categorized according to Mishra's classification system vs different non-surgical control treatments (ACE: Acetaminophen, BMC: Bone Marrow Concentrate, COR: Corticosteroids, HA: Hyaluronic Acid, NSAID: Non-steroidal Anti-Inflammatory Drugs, OZO: Ozone therapy, and PBO: Placebo) on WOMAC Physical Function at the following time points: A) 6 months, and B) 12 months. The vertical line represents the pooled effect estimate. Contour lines indicate regions of statistical significance ($p<0.01$, $p<0.05$, and $p<0.10$). Asymmetry in the distribution of studies may suggest potential publication bias or small-study effects.

A)

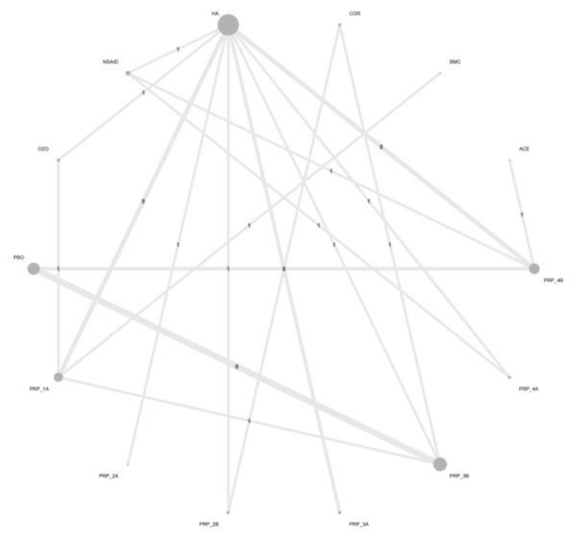

B)

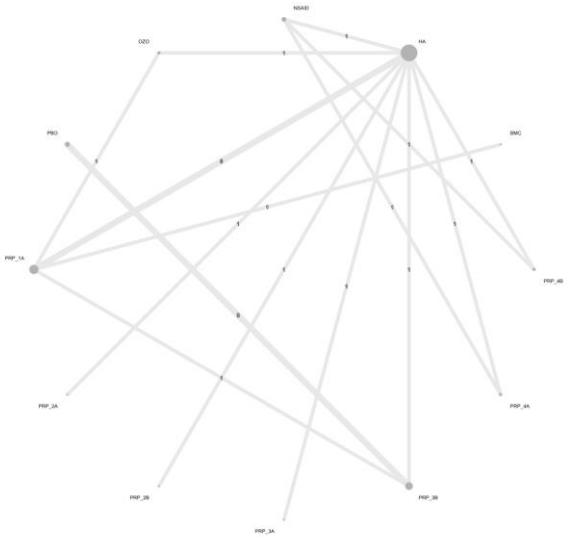

Figure S58. Network plot representing direct comparisons among interventions included in the network meta-analysis for WOMAC Physical Function at the following time points: A) 6 months, and B) 12 months. PRP treatments were categorized according to Mishra's classification system vs different non-surgical control treatments (ACE: Acetaminophen, BMC: Bone Marrow Concentrate, COR: Corticosteroids, HA: Hyaluronic Acid, NSAID: Non-steroidal Anti-Inflammatory Drugs, and OZO: Ozone therapy). Node size is proportional to the total number of participants receiving each intervention, and edge thickness reflects the number of studies contributing to each direct comparison.

A)

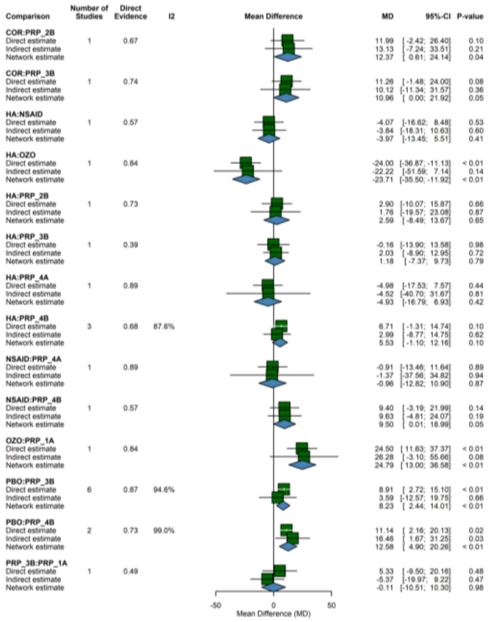

B)

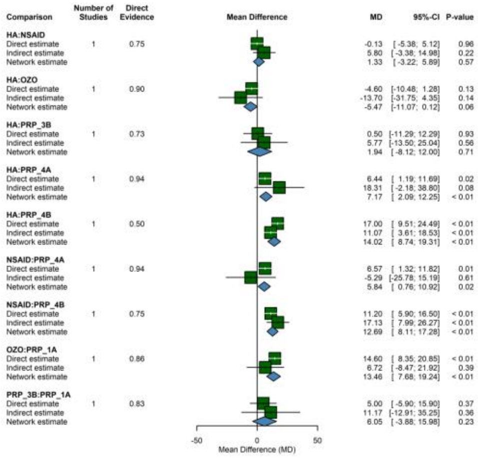

Figure S59. Node-split forest plot representing inconsistency assessment in the network meta-analysis of the effect of PRP categorized according to Mishra's classification system vs different non-surgical control treatments (ACE: Acetaminophen, BMC: Bone Marrow Concentrate, COR: Corticosteroids, HA: Hyaluronic Acid, NSAID: Non-steroidal Anti-Inflammatory Drugs, OZO: Ozone therapy, and PBO: Placebo) on WOMAC Physical Function at the following time points: A) 6 months, and B) 12 months. Each treatment comparison includes both direct and indirect estimates for mean difference (MD) with corresponding 95% confidence intervals.

A)

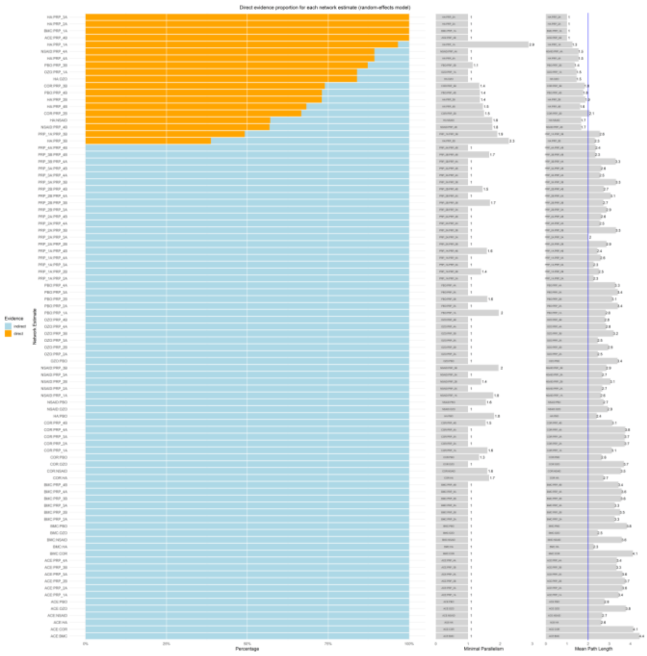

B)

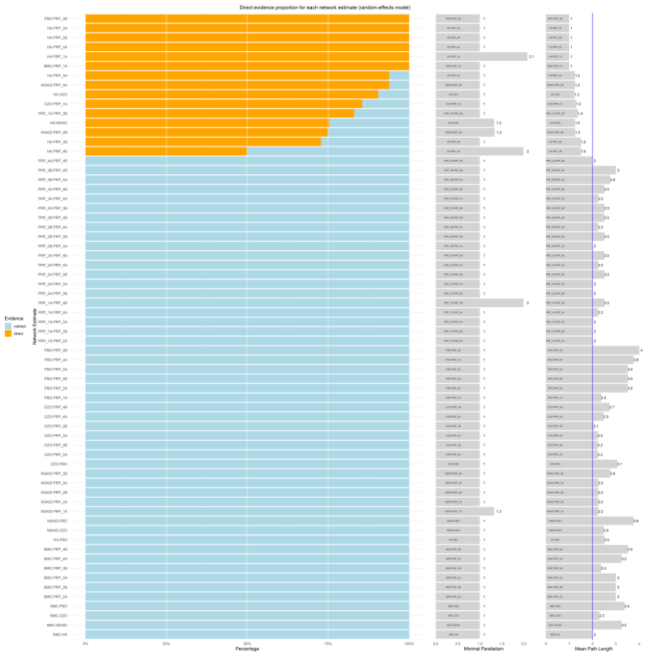

Figure S60. Direct evidence plot representing direct evidence proportion for each assessment corresponding to the network meta-analysis of the effect of PRP categorized according to Mishra's classification system vs different non-surgical control treatments (ACE: Acetaminophen, BMC: Bone Marrow Concentrate, COR: Corticosteroids, HA: Hyaluronic Acid, NSAID: Non-steroidal Anti-Inflammatory Drugs, OZO: Ozone therapy, and PBO: Placebo) on WOMAC Physical Function at the following time points: A) 6 months, and B) 12 months. Key geometry metrics depicted include minimal path length, representing the shortest distance between nodes, and mean path length, reflecting the shortest parallel connections between nodes, and mean path length, quantifying the average shortest path across all pairs of interventions within the network.

WOMAC Total

A)

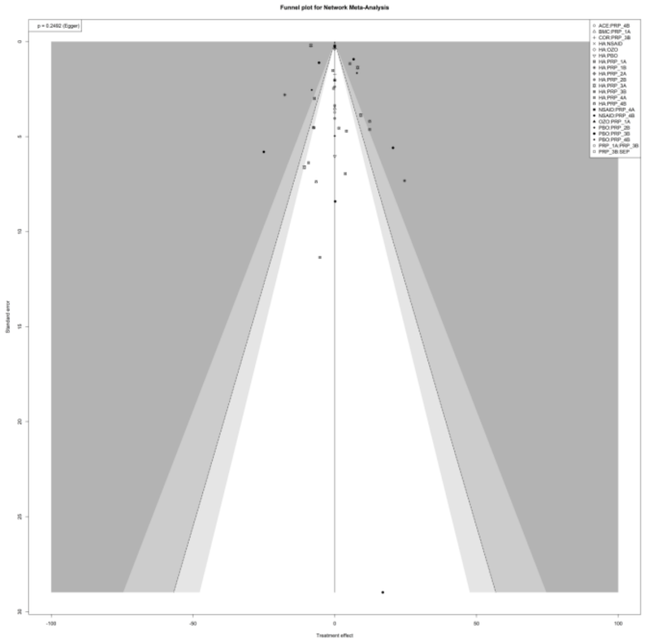

B)

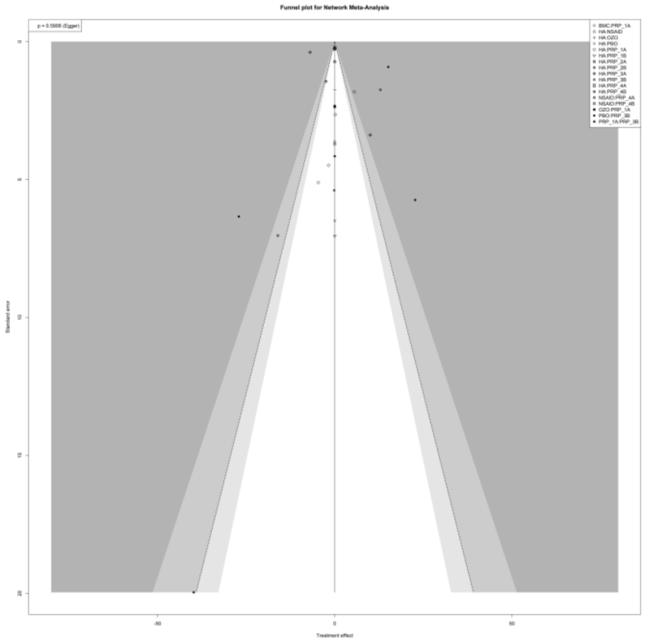

Figure S61. Contour enhanced funnel plot for trials of the effect of PRP categorized according to Mishra's classification system vs different non-surgical control treatments (ACE: Acetaminophen, ARTHRO: Arthroscopy, BMC: Bone Marrow Concentrate, COR: Corticosteroids, HA: Hyaluronic Acid, NSAID: Non-steroidal Anti-Inflammatory Drugs, OZO: Ozone therapy, PBO: Placebo, and SEP: Structured Exercise Program) on WOMAC Total at the following time points: A) 6 months, and B) 12 months. The vertical line represents the pooled effect estimate. Contour lines indicate regions of statistical significance (*p*<0.01, *p*<0.05, and *p*<0.10). Asymmetry in the distribution of studies may suggest potential publication bias or small-study effects.

A)

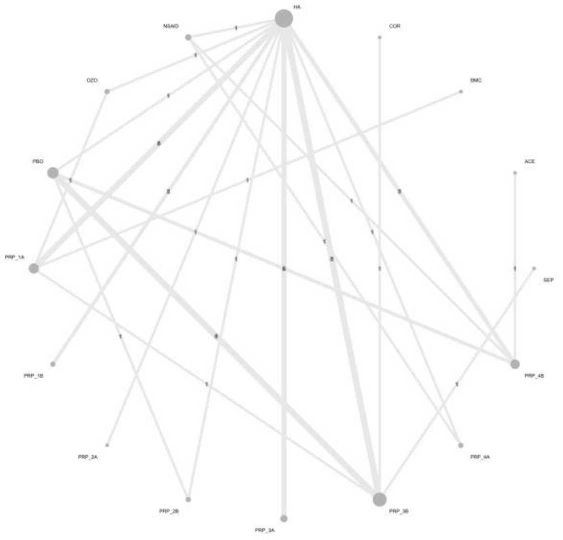

B)

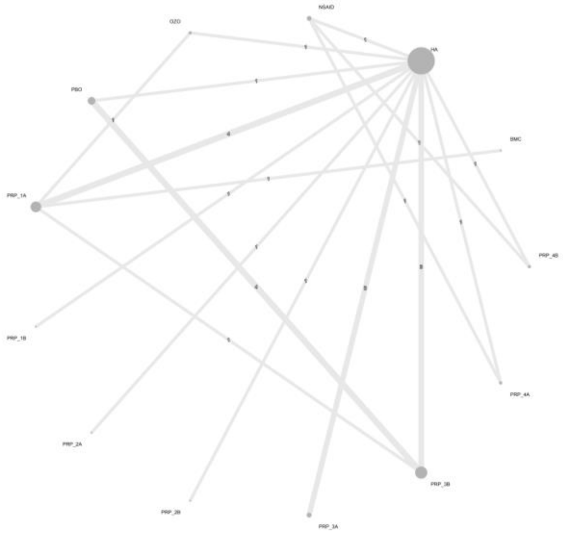

Figure S62. Network plot representing direct comparisons among interventions included in the network meta-analysis for WOMAC Total at the following time points: A) 6 months, and B) 12 months. PRP treatments were categorized according to Mishra's classification system vs different non-surgical control treatments (ACE: Acetaminophen, BMC: Bone Marrow Concentrate, COR: Corticosteroids, HA: Hyaluronic Acid, NSAID: Non-steroidal Anti-Inflammatory Drugs, OZO: Ozone therapy, and PBO: Placebo). Node size is proportional to the total number of participants receiving each intervention, and edge thickness reflects the number of studies contributing to each direct comparison.

A)

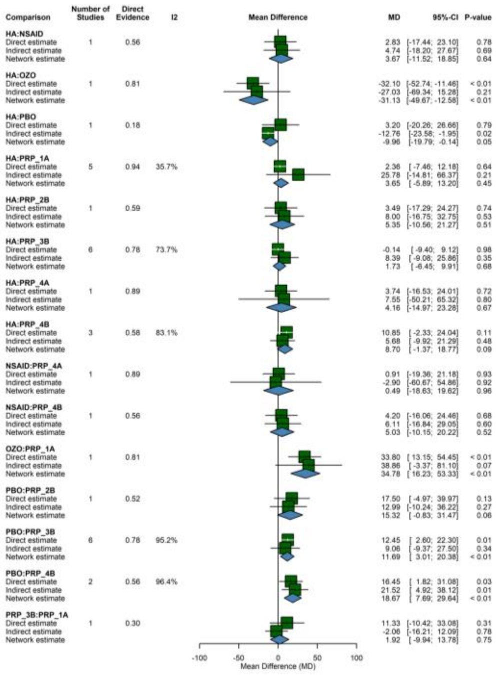

B)

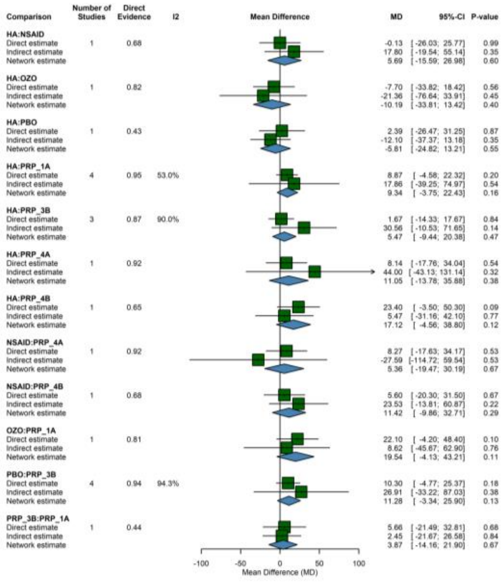

Figure S63. Node-split forest plot representing inconsistency assessment in the network meta-analysis of the effect of PRP categorized according to Mishra's classification system vs different non-surgical control treatments (ACE: Acetaminophen, BMC: Bone Marrow Concentrate, COR: Corticosteroids, HA: Hyaluronic Acid, NSAID: Non-steroidal Anti-Inflammatory Drugs, OZO: Ozone therapy, and PBO: Placebo) on WOMAC Total at the following time points: A) 6 months, and B) 12 months. Each treatment comparison includes both direct and indirect estimates for mean difference (MD) with corresponding 95% confidence intervals.

A)

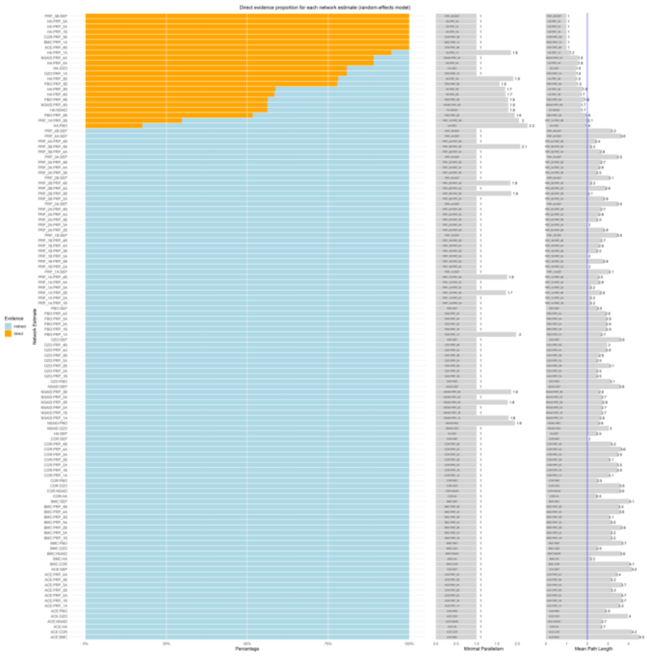

B)

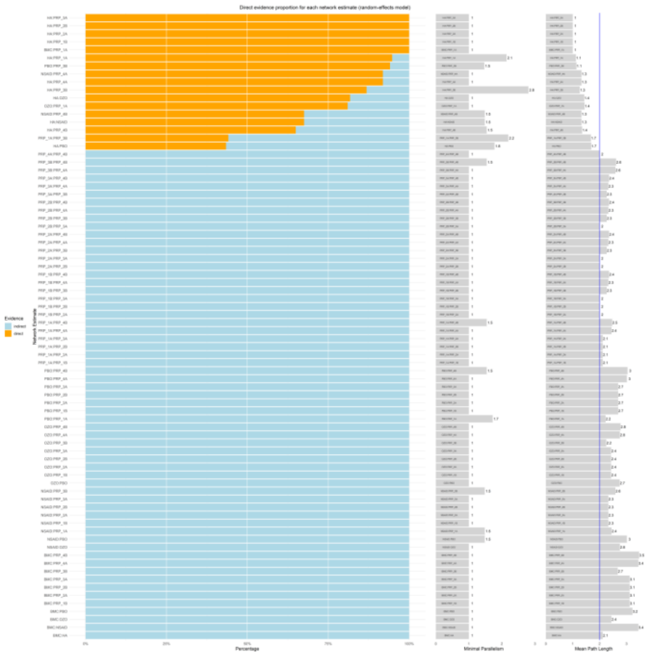

Figure S64. Direct evidence plot representing direct evidence proportion for each assessment corresponding to the network meta-analysis of the effect of PRP categorized according to Mishra's classification system vs different non-surgical control treatments (ACE: Acetaminophen, BMC: Bone Marrow Concentrate, COR: Corticosteroids, HA: Hyaluronic Acid, NSAID: Non-steroidal Anti-Inflammatory Drugs, OZO: Ozone therapy, and PBO: Placebo) on WOMAC Total at the following time points: A) 6 months, and B) 12 months. Key geometry metrics depicted include minimal path length, representing the shortest distance between nodes, and mean path length, reflecting the shortest parallel connections between nodes, and mean path length, quantifying the average shortest path across all pairs of interventions within the network.

KOOS Pain

A)

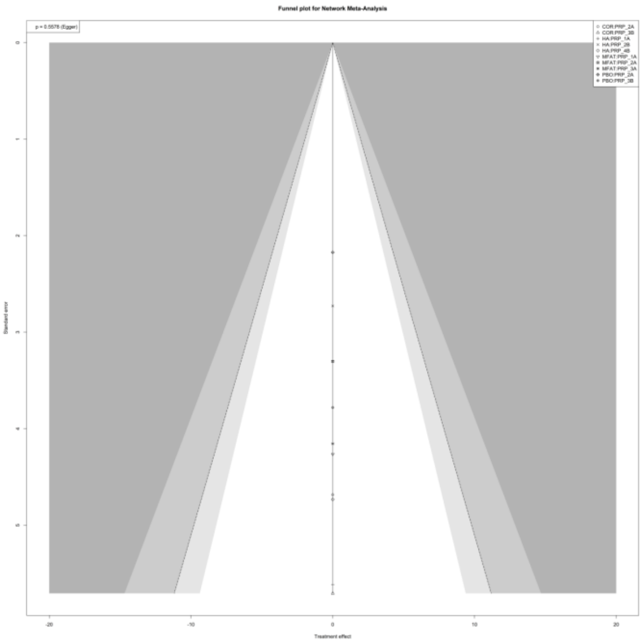

B)

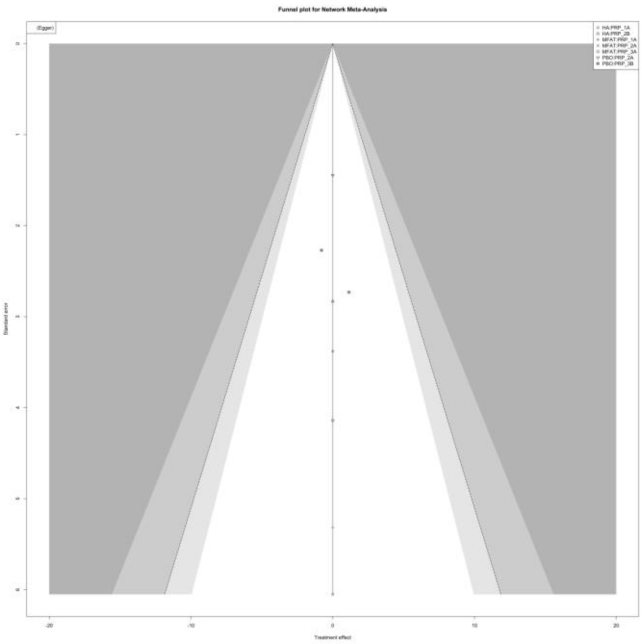

Figure S65. Contour enhanced funnel plot for trials of the effect of PRP categorized according to Mishra's classification system vs different non-surgical control treatments (COR: Corticosteroids, HA: Hyaluronic Acid, MFAT: Microfragmented Adipose Tissue, and PBO: Placebo) on KOOS Pain at the following time points: A) 6 months, and B) 12 months. The vertical line represents the pooled effect estimate. Contour lines indicate regions of statistical significance ($p<0.01$, $p<0.05$, and $p<0.10$). Asymmetry in the distribution of studies may suggest potential publication bias or small-study effects.

A)

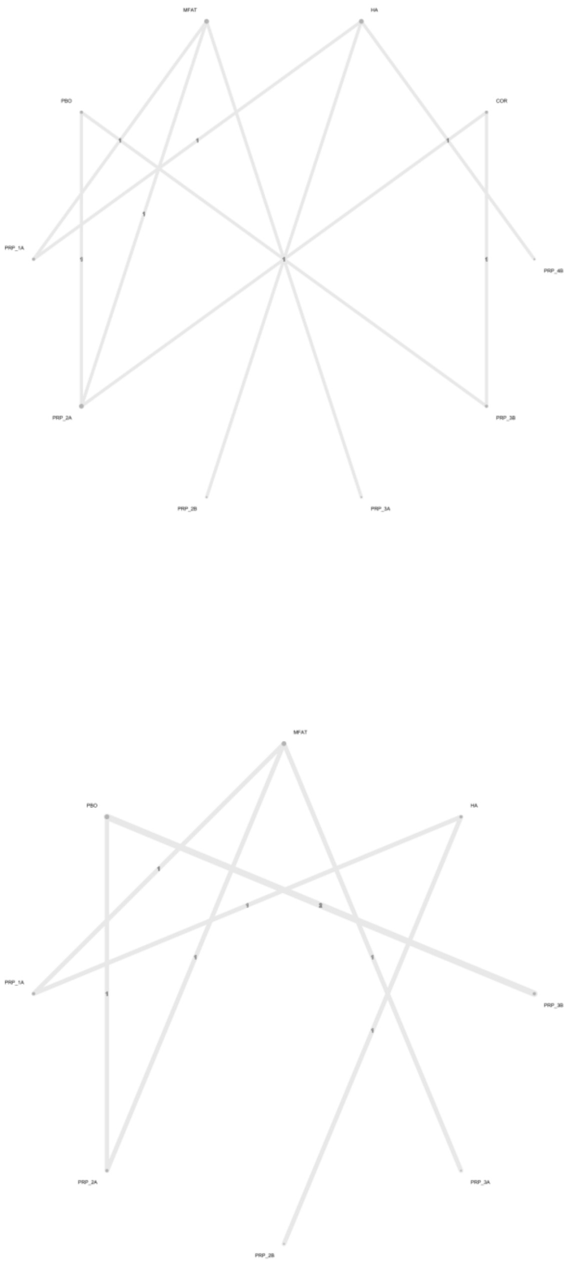

B)

Figure S66. Network plot representing direct comparisons among interventions included in the network meta-analysis for KOOS Pain at the following time points: A) 6 months, and B) 12 months. PRP treatments were categorized according to Mishra's classification system vs different non-surgical control treatments (COR: Corticosteroids, HA: Hyaluronic Acid, MFAT: Microfragmented Adipose Tissue, and PBO: Placebo). Node size is proportional to the total number of participants receiving each intervention, and edge thickness reflects the number of studies contributing to each direct comparison.

A)

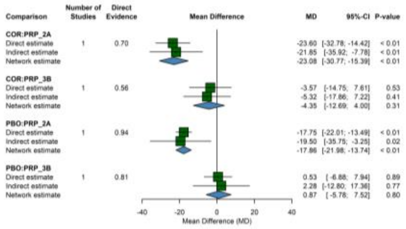

B)

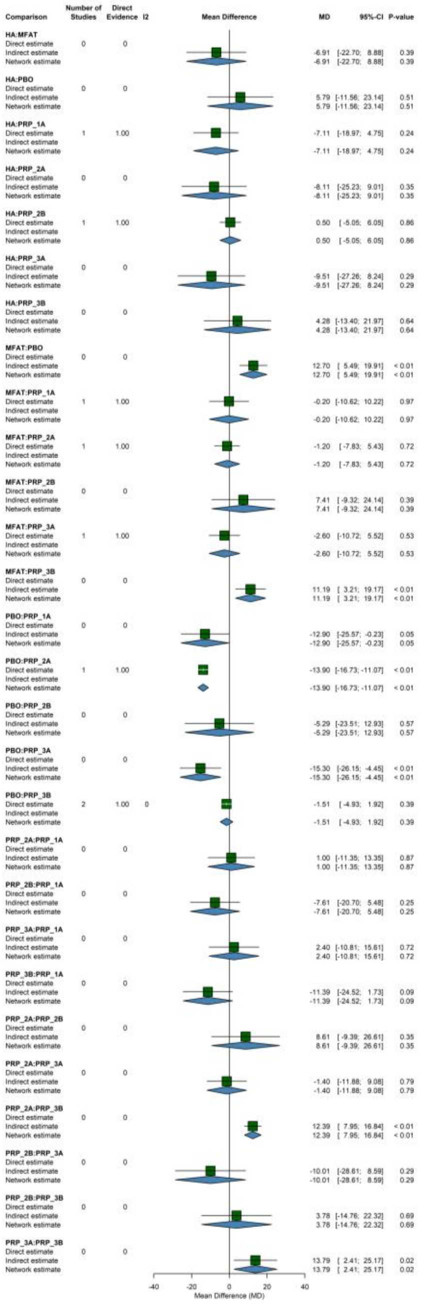

Figure S67. Node-split forest plot representing inconsistency assessment in the network meta-analysis of the effect of PRP categorized according to Mishra's classification system vs different non-surgical control treatments (COR: Corticosteroids, HA: Hyaluronic Acid, MFAT: Microfragmented Adipose Tissue, and PBO: Placebo) on KOOS Pain at the following time points: A) 6 months, and B) 12 months. Each treatment comparison includes both direct and indirect estimates for mean difference (MD) with corresponding 95% confidence intervals.

A)

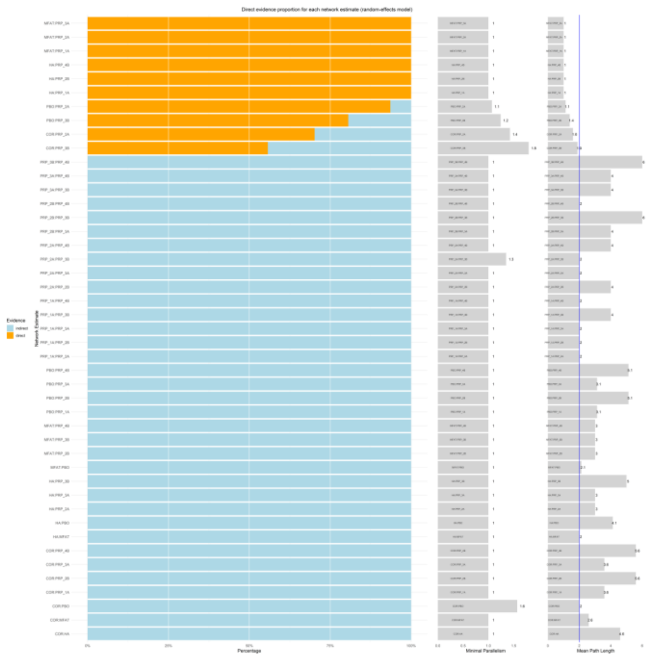

B)

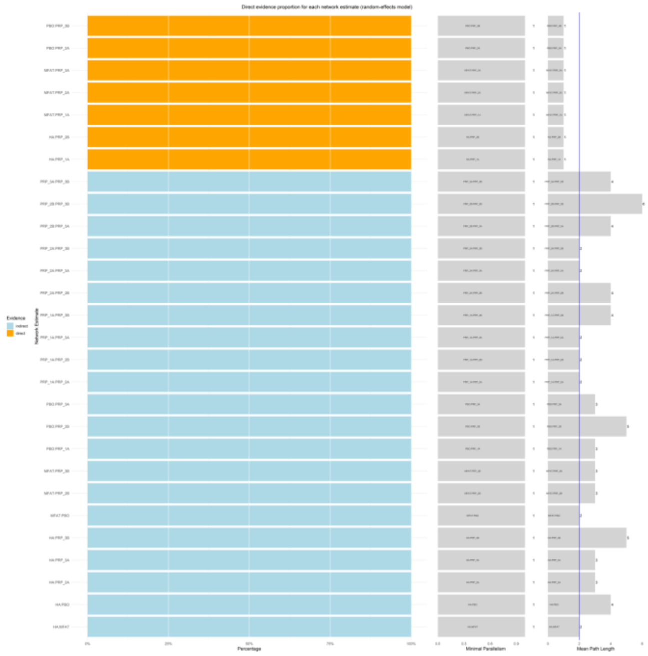

Figure S68. Direct evidence plot representing direct evidence proportion for each assessment corresponding to the network meta-analysis of the effect of PRP categorized according to Mishra's classification system vs different non-surgical control treatments (COR: Corticosteroids, HA: Hyaluronic Acid, MFAT: Microfragmented Adipose Tissue, and PBO: Placebo) on KOOS Pain at the following time points: A) 6 months, and B) 12 months. Key geometry metrics depicted include minimal path length, representing the shortest distance between nodes, and mean path length, reflecting the shortest parallel connections between nodes, and mean path length, quantifying the average shortest path across all pairs of interventions within the network.

KOOS Activities of Daily Living

A)

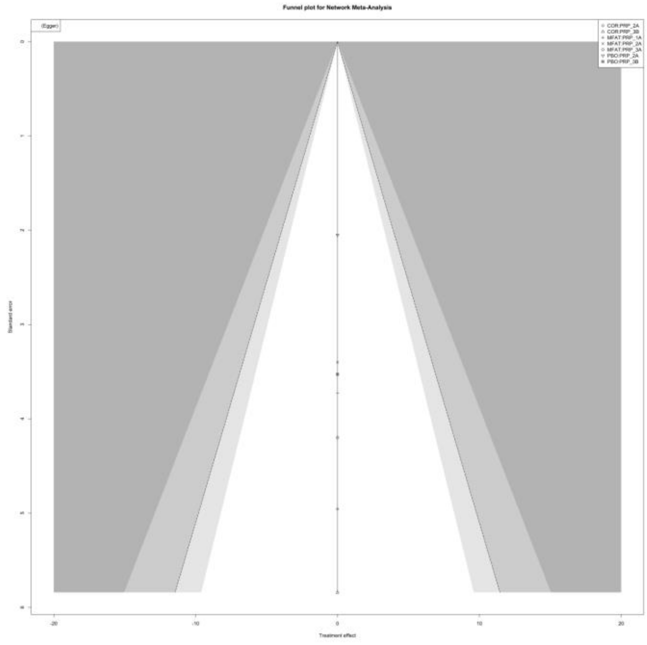

B)

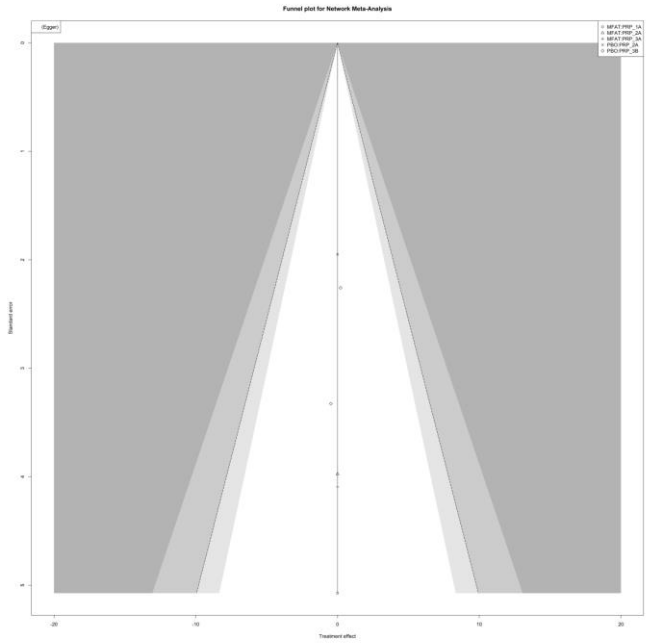

Figure S69. Contour enhanced funnel plot for trials of the effect of PRP categorized according to Mishra's classification system vs different non-surgical control treatments (COR: Corticosteroids, HA: Hyaluronic Acid, MFAT: Microfragmented Adipose Tissue, and PBO: Placebo) on KOOS Activities of Daily Living at the following time points: A) 6 months, and B) 12 months. The vertical line represents the pooled effect estimate. Contour lines indicate regions of statistical significance (*p*<0.01, *p*<0.05, and *p*<0.10). Asymmetry in the distribution of studies may suggest potential publication bias or small-study effects.

A)

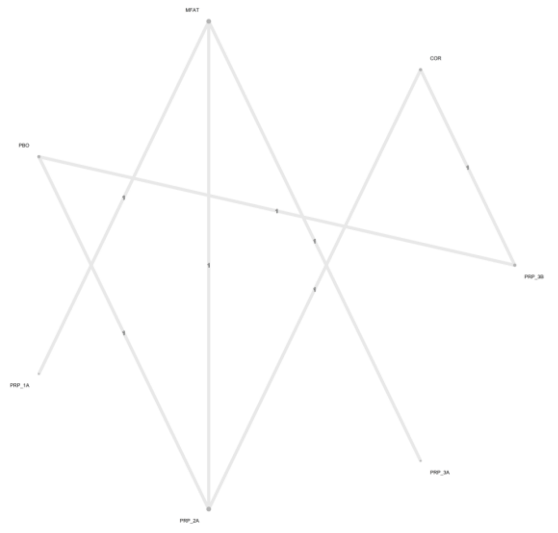

B)

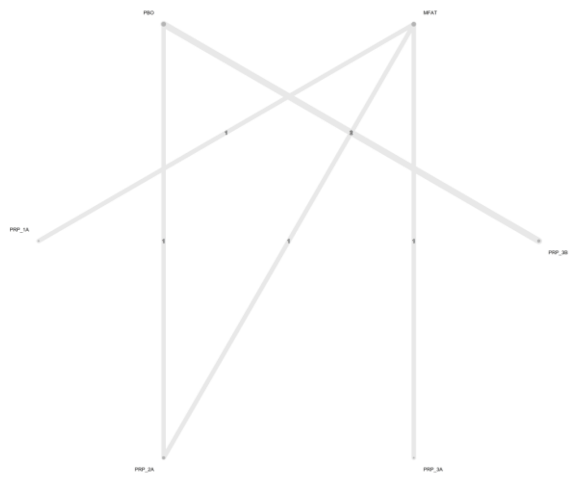

Figure S70. Network plot representing direct comparisons among interventions included in the network meta-analysis for KOOS Activities of Daily Living at the following time points: A) 6 months, and B) 12 months. PRP treatments were categorized according to Mishra's classification system vs different non-surgical control treatments (COR: Corticosteroids, HA: Hyaluronic Acid, MFAT: Microfragmented Adipose Tissue, and PBO: Placebo). Node size is proportional to the total number of participants receiving each intervention, and edge thickness reflects the number of studies contributing to each direct comparison.

A)

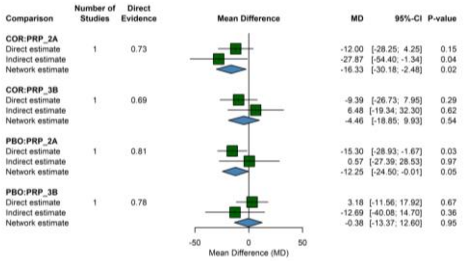

B)

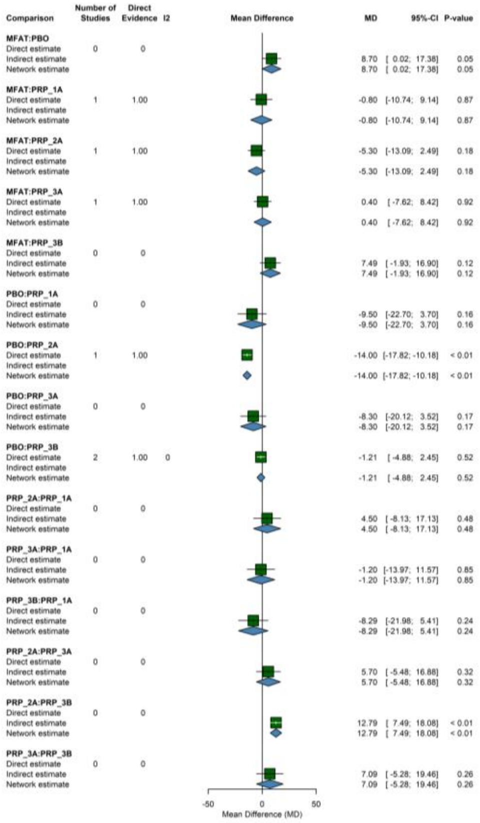

Figure S71. Node-split forest plot representing inconsistency assessment in the network meta-analysis of the effect of PRP categorized according to Mishra's classification system vs different non-surgical control treatments (COR: Corticosteroids, HA: Hyaluronic Acid, MFAT: Microfragmented Adipose Tissue, and PBO: Placebo) on KOOS Activities of Daily Living at the following time points: A) 6 months, and B) 12 months. Each treatment comparison includes both direct and indirect estimates for mean difference (MD) with corresponding 95% confidence intervals.

A)

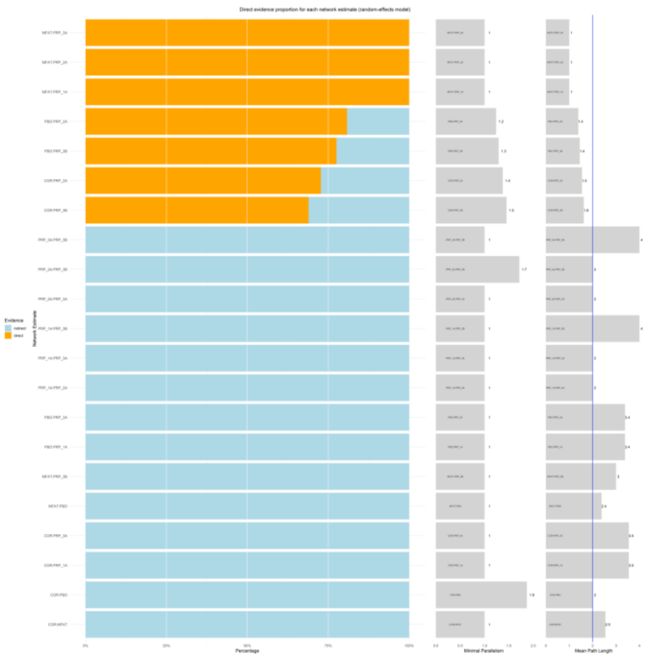

B)

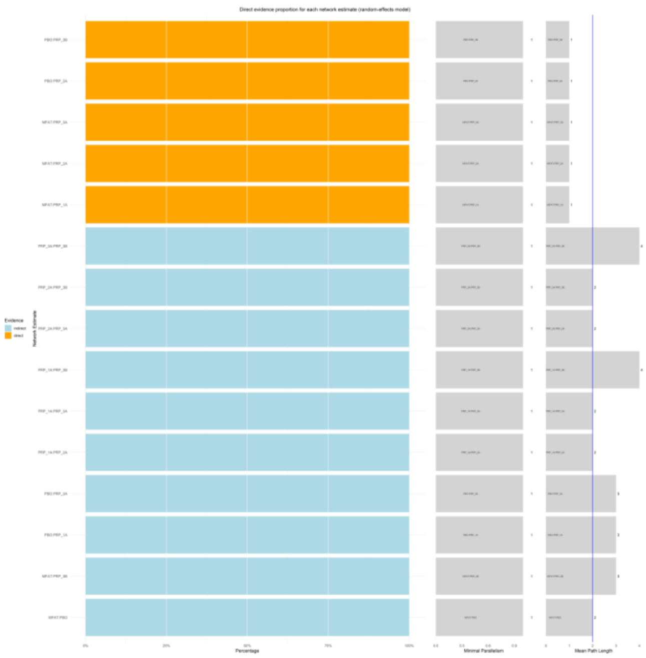

Figure S72. Direct evidence plot representing direct evidence proportion for each assessment corresponding to the network meta-analysis of the effect of PRP categorized according to Mishra’s classification system vs different non-surgical control treatments (COR: Corticosteroids, HA: Hyaluronic Acid, MFAT: Microfragmented Adipose Tissue, and PBO: Placebo) on KOOS Activities of Daily Living at the following time points: A) 6 months, and B) 12 months. Key geometry metrics depicted include minimal path length, representing the shortest distance between nodes, and mean path length, reflecting the shortest parallel connections between nodes, and mean path length, quantifying the average shortest path across all pairs of interventions within the network.

KOOS Sport and Recreation Function

A)

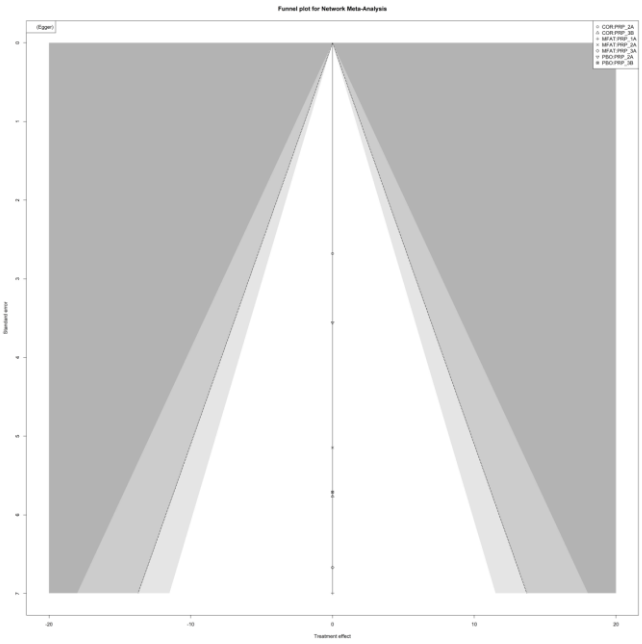

B)

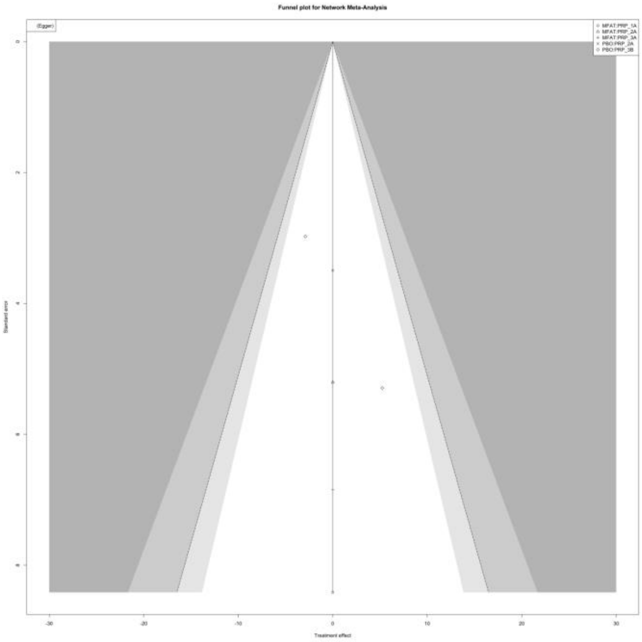

Figure S73. Contour enhanced funnel plot for trials of the effect of PRP categorized according to Mishra's classification system vs different non-surgical control treatments (COR: Corticosteroids, HA: Hyaluronic Acid, MFAT: Microfragmented Adipose Tissue, and PBO: Placebo) on KOOS Sport and Recreation Function at the following time points: A) 6 months, and B) 12 months. The vertical line represents the pooled effect estimate. Contour lines indicate regions of statistical significance (*p*<0.01, *p*<0.05, and *p*<0.10). Asymmetry in the distribution of studies may suggest potential publication bias or small-study effects.

A)

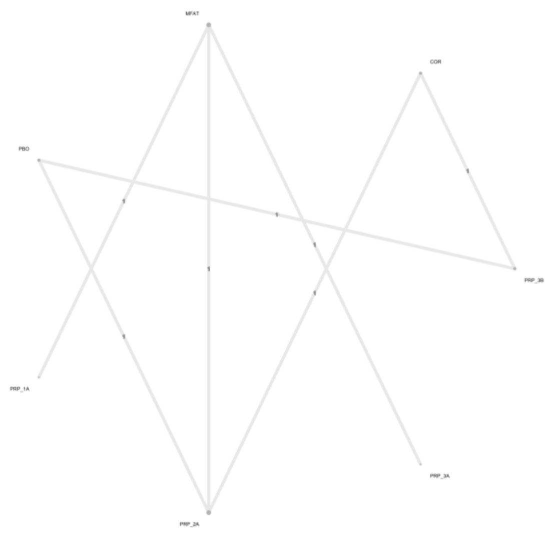

B)

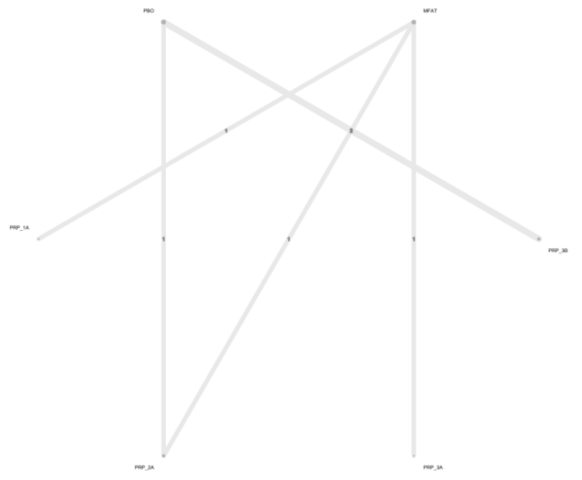

Figure S74. Network plot representing direct comparisons among interventions included in the network meta-analysis for KOOS Sport and Recreation Function at the following time points: A) 6 months, and B) 12 months. PRP treatments were categorized according to Mishra's classification system vs different non-surgical control treatments (COR: Corticosteroids, HA: Hyaluronic Acid, MFAT: Microfragmented Adipose Tissue, and PBO: Placebo). Node size is proportional to the total number of participants receiving each intervention, and edge thickness reflects the number of studies contributing to each direct comparison.

A)

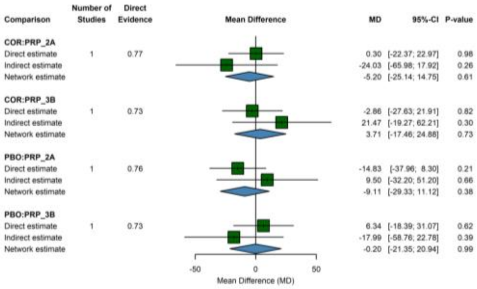

B)

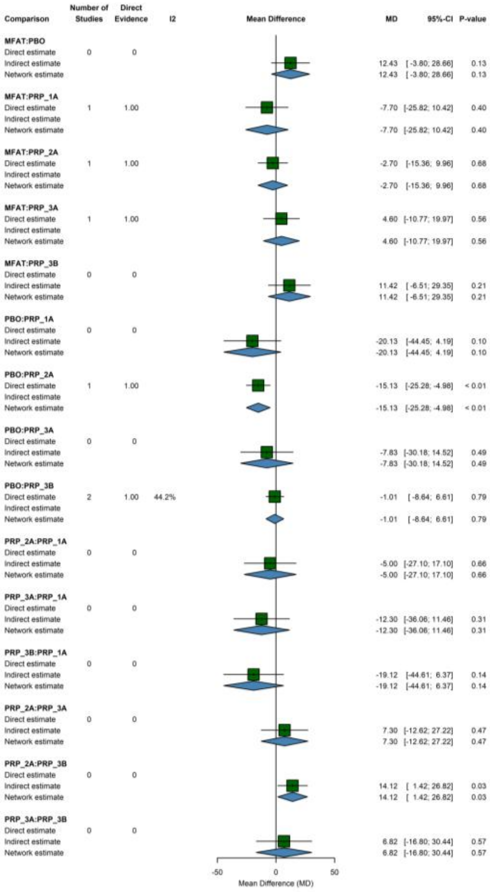

Figure S75. Node-split forest plot representing inconsistency assessment in the network meta-analysis of the effect of PRP categorized according to Mishra's classification system vs different non-surgical control treatments (COR: Corticosteroids, HA: Hyaluronic Acid, MFAT: Microfragmented Adipose Tissue, and PBO: Placebo) on KOOS Sport and Recreation Function at the following time points: A) 6 months, and B) 12 months. Each treatment comparison includes both direct and indirect estimates for mean difference (MD) with corresponding 95% confidence intervals.

A)

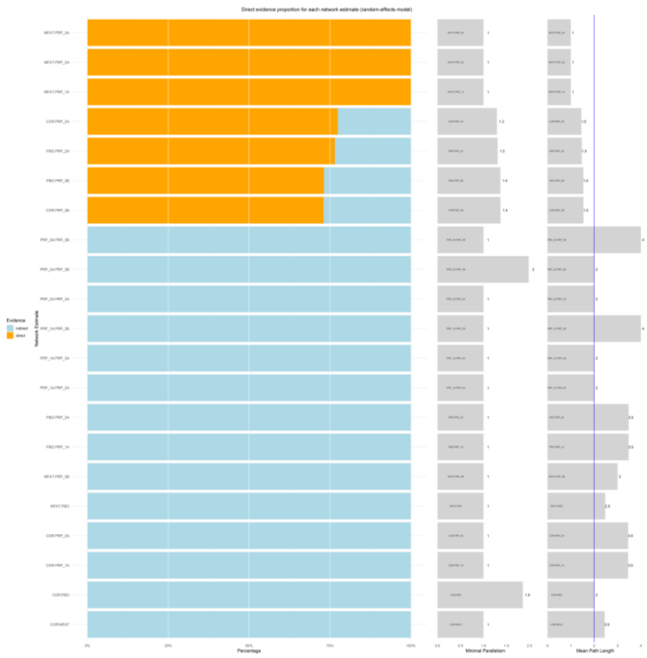

B)

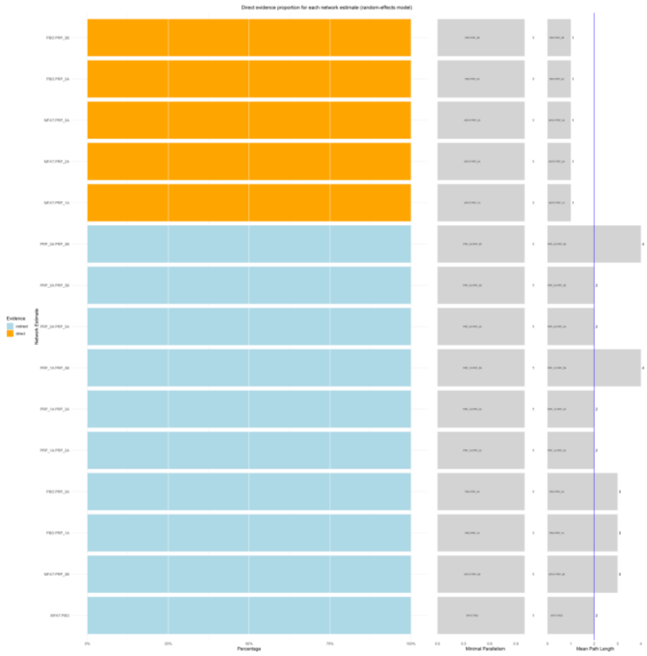

Figure S76. Direct evidence plot representing direct evidence proportion for each assessment corresponding to the network meta-analysis of the effect of PRP categorized according to Mishra's classification system vs different non-surgical control treatments (COR: Corticosteroids, HA: Hyaluronic Acid, MFAT: Microfragmented Adipose Tissue, and PBO: Placebo) on KOOS Sport and Recreation Function at the following time points: A) 6 months, and B) 12 months. Key geometry metrics depicted include minimal path length, representing the shortest distance between nodes, and mean path length, reflecting the shortest parallel connections between nodes, and mean path length, quantifying the average shortest path across all pairs of interventions within the network.

KOOS Knee-Related Quality of Life

A)

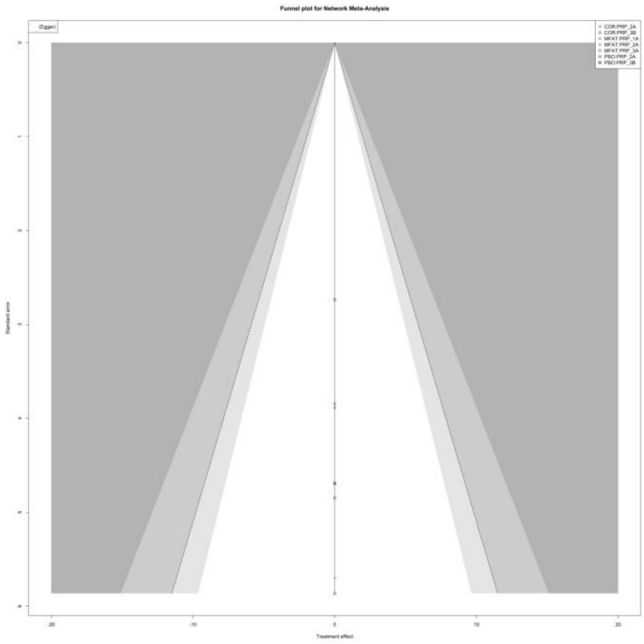

B)

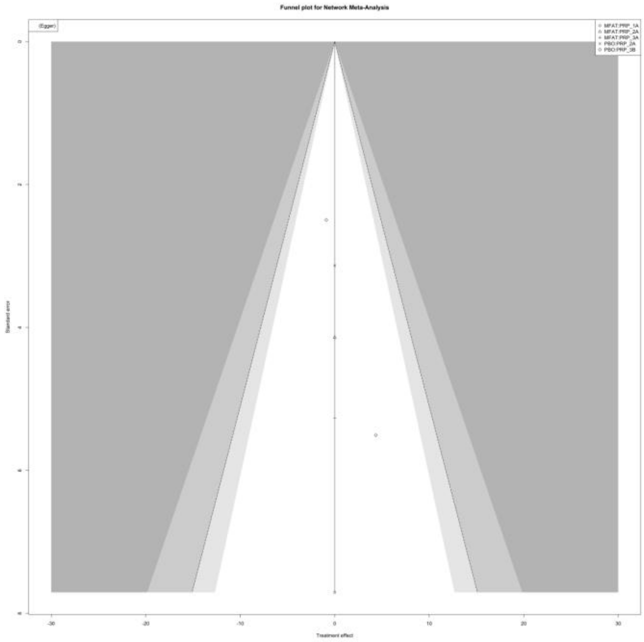

Figure S77. Contour enhanced funnel plot for trials of the effect of PRP categorized according to Mishra's classification system vs different non-surgical control treatments (COR: Corticosteroids, HA: Hyaluronic Acid, MFAT: Microfragmented Adipose Tissue, and PBO: Placebo) on KOOS Knee-Related Quality of Life at the following time points: A) 6 months, and B) 12 months. The vertical line represents the pooled effect estimate. Contour lines indicate regions of statistical significance (p<0.01, p<0.05, and p<0.10). Asymmetry in the distribution of studies may suggest potential publication bias or small-study effects.

A)

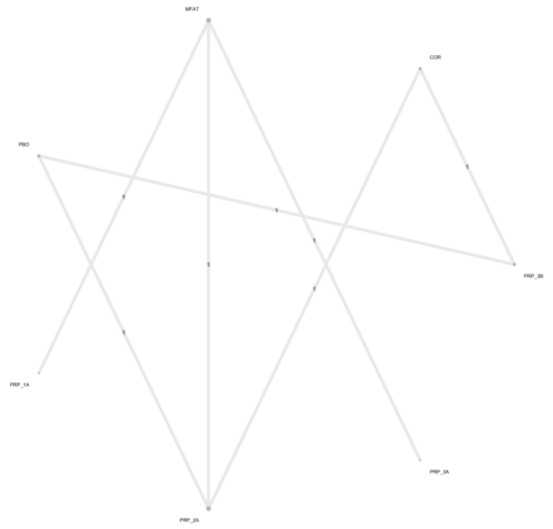

B)

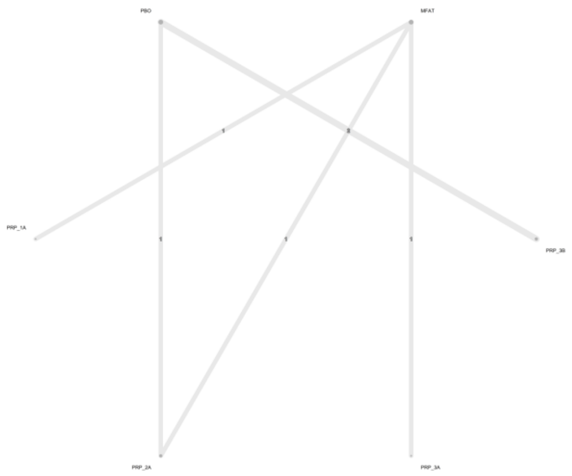

Figure S78. Network plot representing direct comparisons among interventions included in the network meta-analysis for KOOS Knee-Related Quality of Life at the following time points: A) 6 months, and B) 12 months. PRP treatments were categorized according to Mishra's classification system vs different non-surgical control treatments (COR: Corticosteroids, HA: Hyaluronic Acid, MFAT: Microfragmented Adipose Tissue, and PBO: Placebo). Node size is proportional to the total number of participants receiving each intervention, and edge thickness reflects the number of studies contributing to each direct comparison.

A)

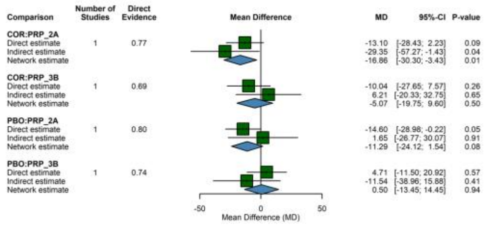

B)

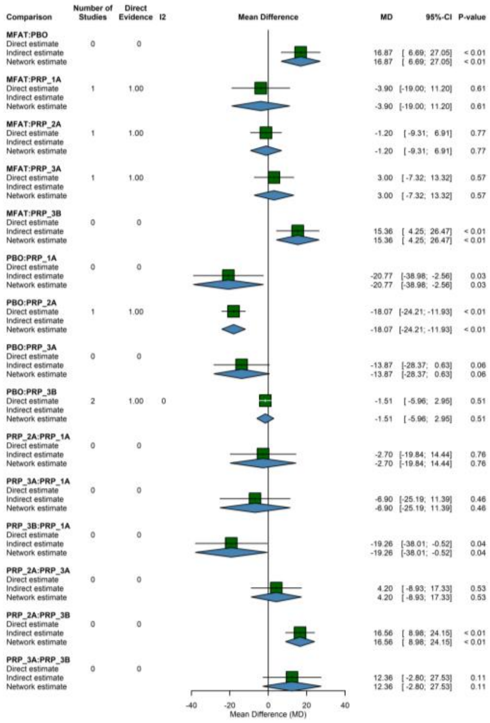

Figure S79. Node-split forest plot representing inconsistency assessment in the network meta-analysis of the effect of PRP categorized according to Mishra's classification system vs different non-surgical control treatments (COR: Corticosteroids, HA: Hyaluronic Acid, MFAT: Microfragmented Adipose Tissue, and PBO: Placebo) on KOOS Knee-Related Quality of Life at the following time points: A) 6 months, and B) 12 months. Each treatment comparison includes both direct and indirect estimates for mean difference (MD) with corresponding 95% confidence intervals.

A)

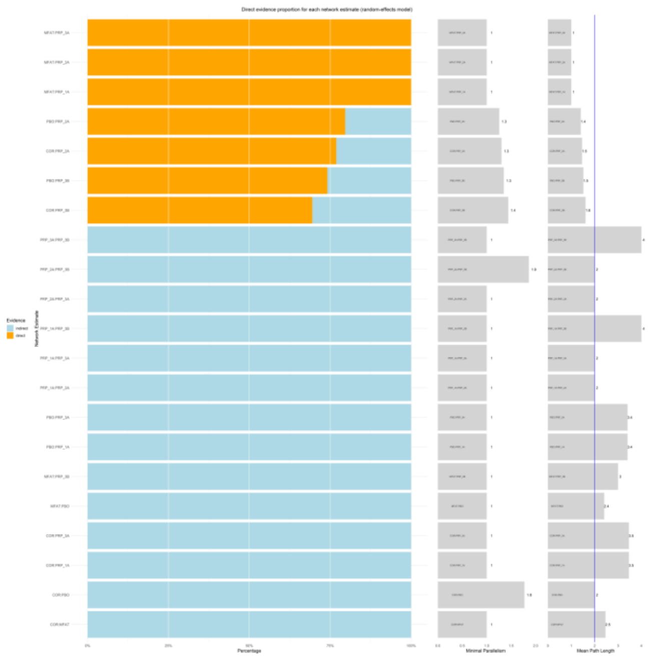

B)

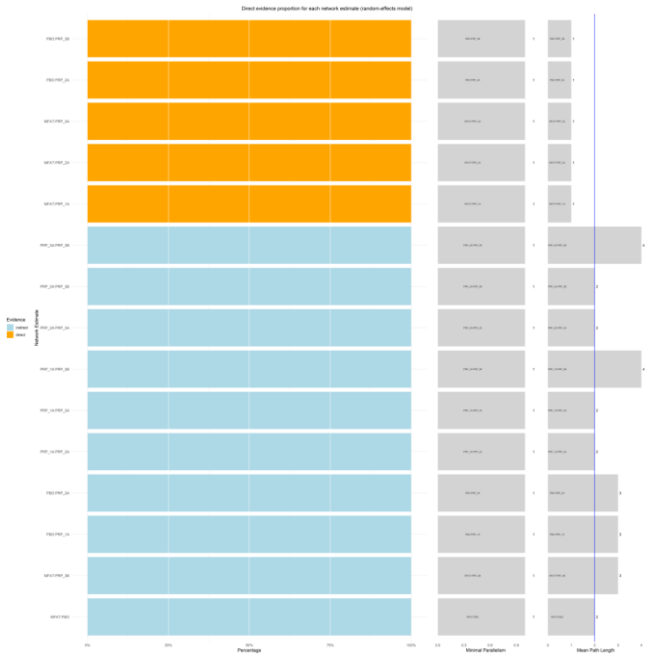

Figure S80. Direct evidence plot representing direct evidence proportion for each assessment corresponding to the network meta-analysis of the effect of PRP categorized according to Mishra's classification system vs different non-surgical control treatments (COR: Corticosteroids, HA: Hyaluronic Acid, MFAT: Microfragmented Adipose Tissue, and PBO: Placebo) on KOOS Knee-Related Quality of Life at the following time points: A) 6 months, and B) 12 months. Key geometry metrics depicted include minimal path length, representing the shortest distance between nodes, and mean path length, reflecting the shortest parallel connections between nodes, and mean path length, quantifying the average shortest path across all pairs of interventions within the network.

KOOS Symptoms

A)

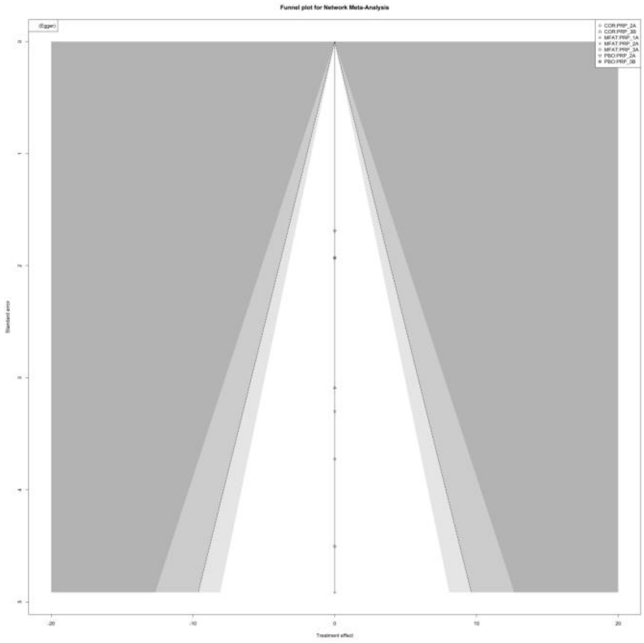

B)

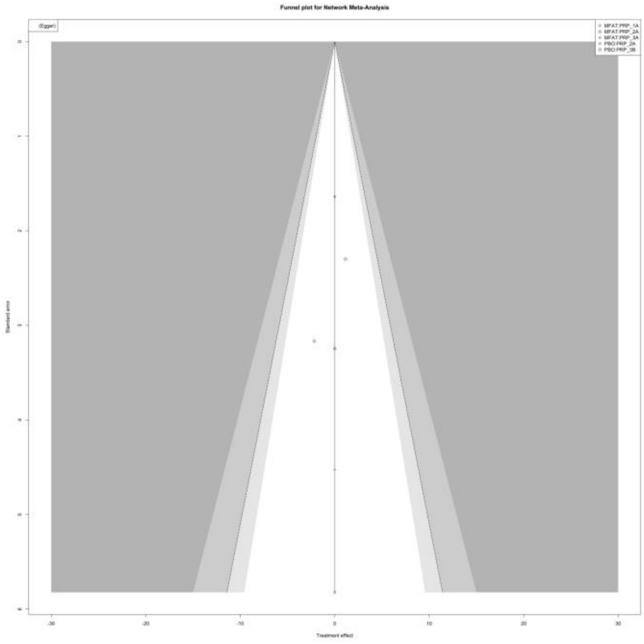

Figure S81. Contour enhanced funnel plot for trials of the effect of PRP categorized according to Mishra's classification system vs different non-surgical control treatments (COR: Corticosteroids, HA: Hyaluronic Acid, MFAT: Microfragmented Adipose Tissue, and PBO: Placebo) on KOOS Symptoms at the following time points: A) 6 months, and B) 12 months. The vertical line represents the pooled effect estimate. Contour lines indicate regions of statistical significance (*p*<0.01, *p*<0.05, and *p*<0.10). Asymmetry in the distribution of studies may suggest potential publication bias or small-study effects.

A)

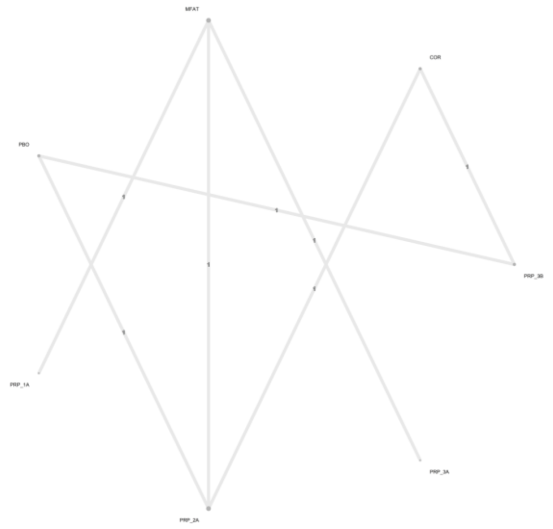

B)

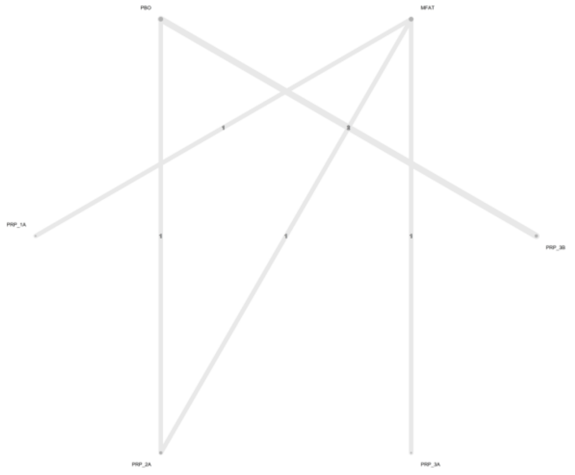

Figure S82. Network plot representing direct comparisons among interventions included in the network meta-analysis for KOOS Symptoms at the following time points: A) 6 months, and B) 12 months. PRP treatments were categorized according to Mishra's classification system vs different non-surgical control treatments (COR: Corticosteroids, HA: Hyaluronic Acid, MFAT: Microfragmented Adipose Tissue, and PBO: Placebo). Node size is proportional to the total number of participants receiving each intervention, and edge thickness reflects the number of studies contributing to each direct comparison.

A)

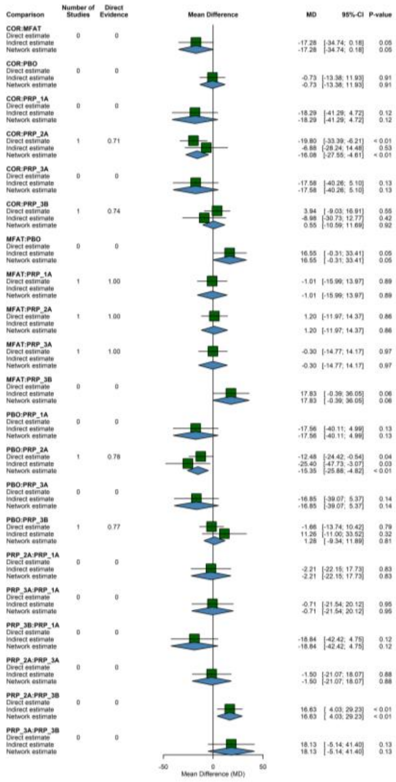

B)

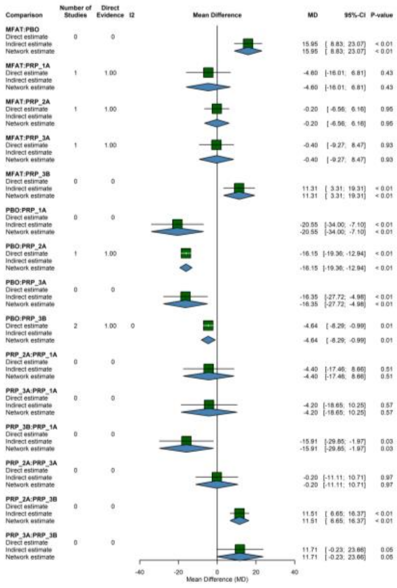

Figure S83. Node-split forest plot representing inconsistency assessment in the network meta-analysis of the effect of PRP categorized according to Mishra's classification system vs different non-surgical control treatments (COR: Corticosteroids, HA: Hyaluronic Acid, MFAT: Microfragmented Adipose Tissue, and PBO: Placebo) on KOOS Symptoms at the following time points: A) 6 months, and B) 12 months. Each treatment comparison includes both direct and indirect estimates for mean difference (MD) with corresponding 95% confidence intervals.

A)

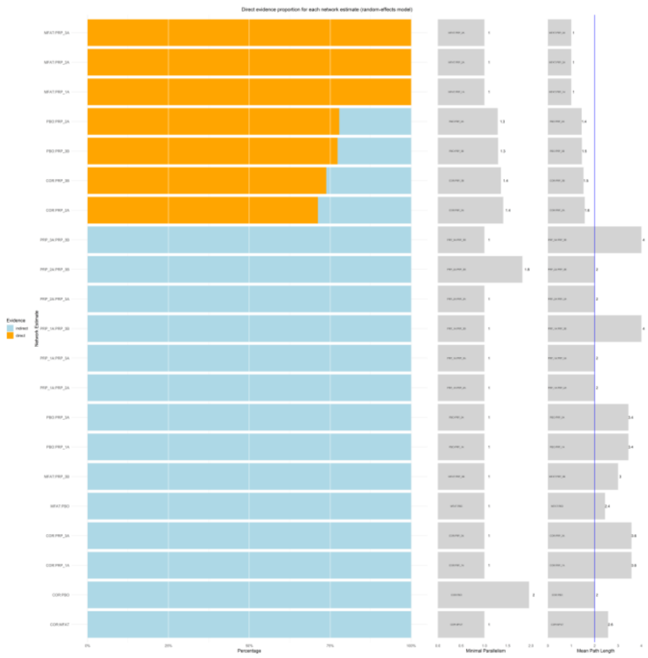

B)

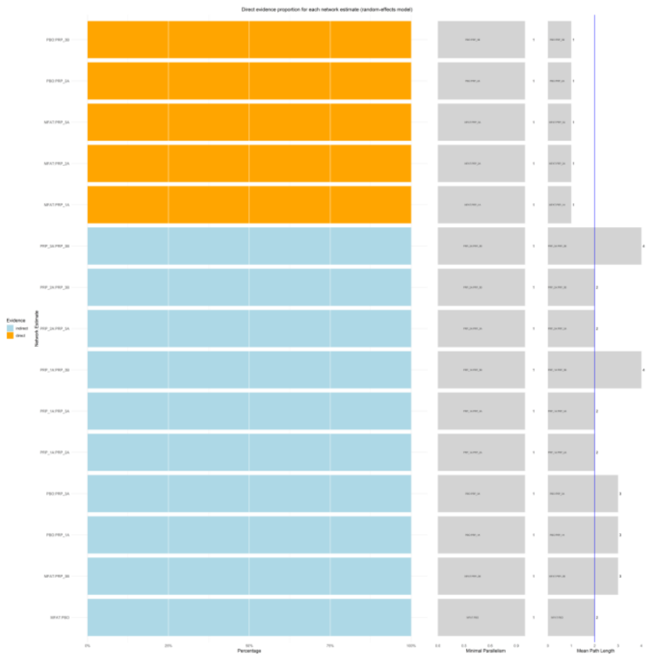

Figure S84. Direct evidence plot representing direct evidence proportion for each assessment corresponding to the network meta-analysis of the effect of PRP categorized according to Mishra's classification system vs different non-surgical control treatments (ACE: Acetaminophen, BMC: Bone Marrow Concentrate, COR: Corticosteroids, HA: Hyaluronic Acid, NSAID: Non-steroidal Anti-Inflammatory Drugs, and OZO: Ozone therapy) on KOOS Symptoms at the following time points: A) 6 months, and B) 12 months. Key geometry metrics depicted include minimal path length, representing the shortest distance between nodes, and mean path length, reflecting the shortest parallel connections between nodes, and mean path length, quantifying the average shortest path across all pairs of interventions within the network.

VAS

A)

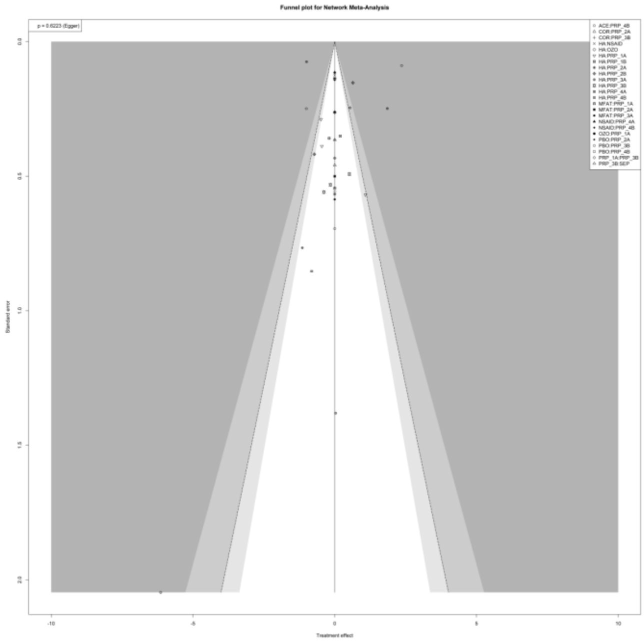

B)

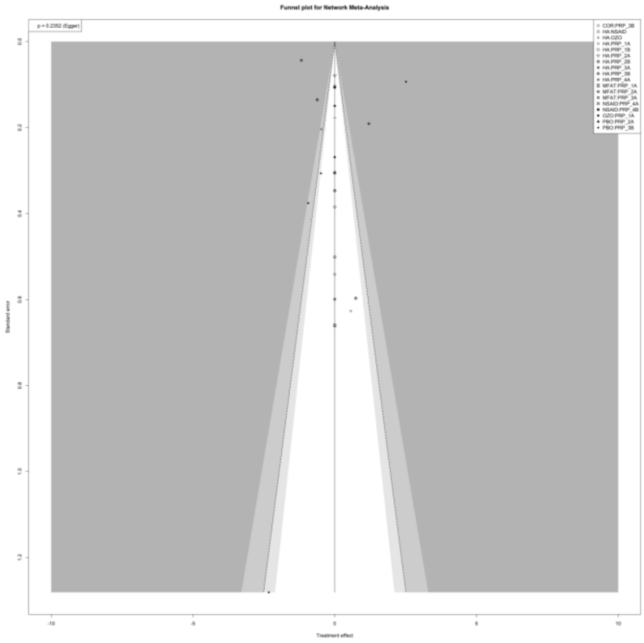

Figure S85. Contour enhanced funnel plot for trials of the effect of PRP categorized according to Mishra's classification system vs different non-surgical control treatments (ACE: Acetaminophen, ARTHRO: Arthroscopy, BMC: Bone Marrow Concentrate, COR: Corticosteroids, HA: Hyaluronic Acid, MFAT: Microfragmented Adipose Tissue, NSAID: Non-steroidal Anti-Inflammatory Drugs, OZO: Ozone therapy, PBO: Placebo, and SEP: Structured Exercise Program) on VAS at the following time points: A) 6 months, and B) 12 months. The vertical line represents the pooled effect estimate. Contour lines indicate regions of statistical significance (*p*<0.01, *p*<0.05, and *p*<0.10). Asymmetry in the distribution of studies may suggest potential publication bias or small-study effects.

A)

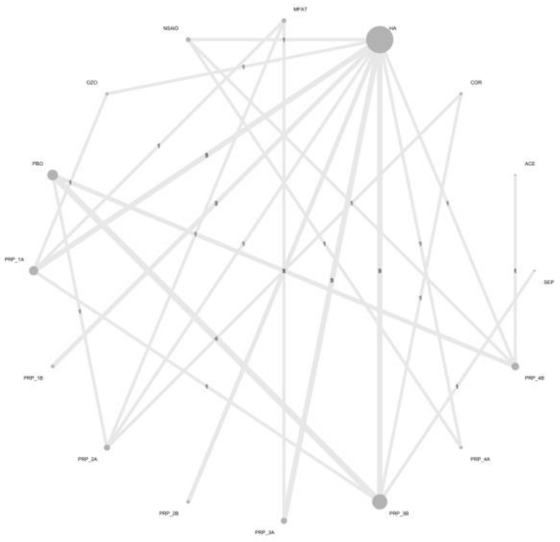

B)

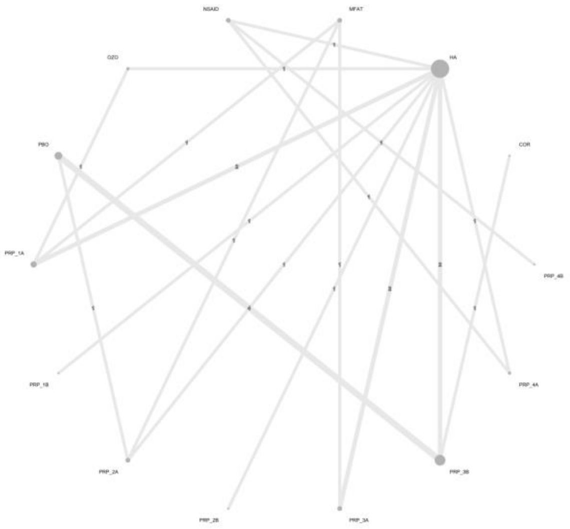

Figure S86. Network plot representing direct comparisons among interventions included in the network meta-analysis for VAS at the following time points: A) 6 months, and B) 12 months. PRP treatments were categorized according to Mishra's classification system vs different non-surgical control treatments (ACE: Acetaminophen, ARTHRO: Arthroscopy, BMC: Bone Marrow Concentrate, COR: Corticosteroids, HA: Hyaluronic Acid, MFAT: Microfragmented Adipose Tissue, NSAID: Non-steroidal Anti-Inflammatory Drugs, OZO: Ozone therapy, PBO: Placebo, and SEP: Structured Exercise Program). Node size is proportional to the total number of participants receiving each intervention, and edge thickness reflects the number of studies contributing to each direct comparison.

A)

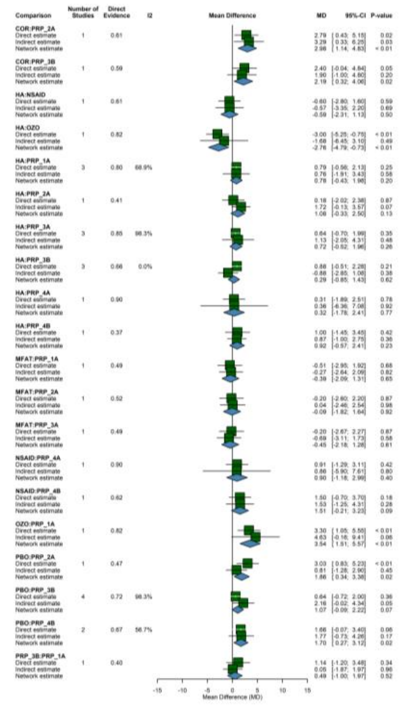

B)

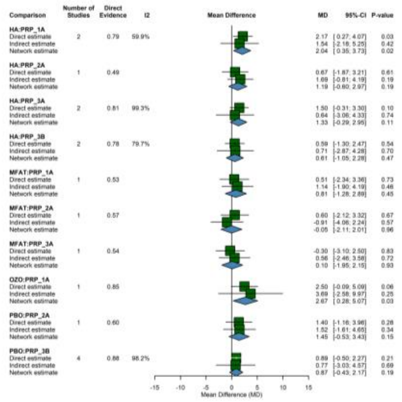

Figure S87. Node-split forest plot representing inconsistency assessment in the network meta-analysis of the effect of PRP categorized according to Mishra’s classification system vs different non-surgical control treatments (ACE: Acetaminophen, ARTHRO: Arthroscopy, BMC: Bone Marrow Concentrate, COR: Corticosteroids, HA: Hyaluronic Acid, MFAT: Microfragmented Adipose Tissue, NSAID: Non-steroidal Anti-Inflammatory Drugs, OZO: Ozone therapy, PBO: Placebo, and SEP: Structured Exercise Program) on VAS at the following time points: A) 6 months, and B) 12 months. Each treatment comparison includes both direct and indirect estimates for mean difference (MD) with corresponding 95% confidence intervals.

A)

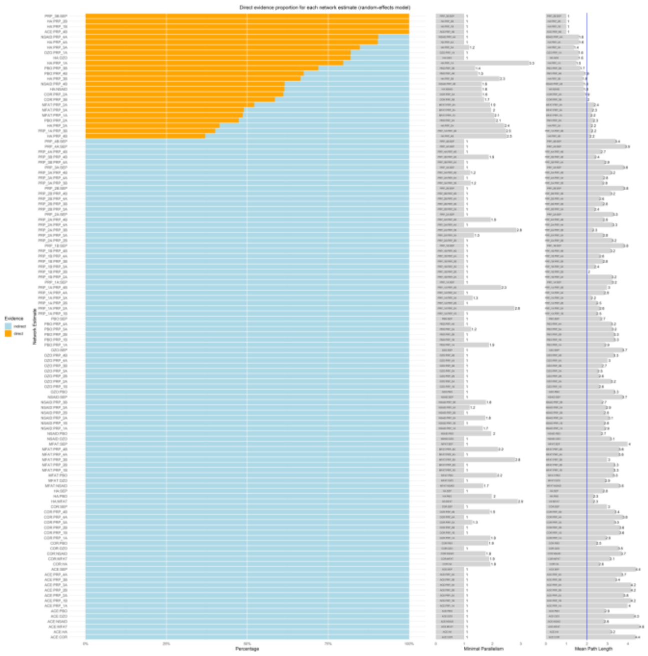

B)

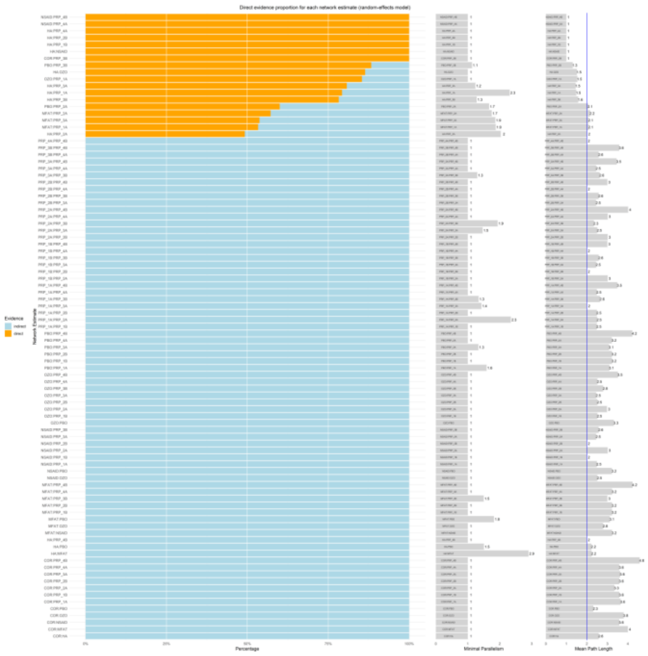

Figure S88. Direct evidence plot representing direct evidence proportion for each assessment corresponding to the network meta-analysis of the effect of PRP categorized according to Mishra's classification system vs different non-surgical control treatments (ACE: Acetaminophen, ARTHRO: Arthroscopy, BMC: Bone Marrow Concentrate, COR: Corticosteroids, HA: Hyaluronic Acid, MFAT: Microfragmented Adipose Tissue, NSAID: Non-steroidal Anti-Inflammatory Drugs, OZO: Ozone therapy, PBO: Placebo, and SEP: Structured Exercise Program) on VAS at the following time points: A) 6 months, and B) 12 months. Key geometry metrics depicted include minimal path length, representing the shortest distance between nodes, and mean path length, reflecting the shortest parallel connections between nodes, and mean path length, quantifying the average shortest path across all pairs of interventions within the network.

IKDC

A)

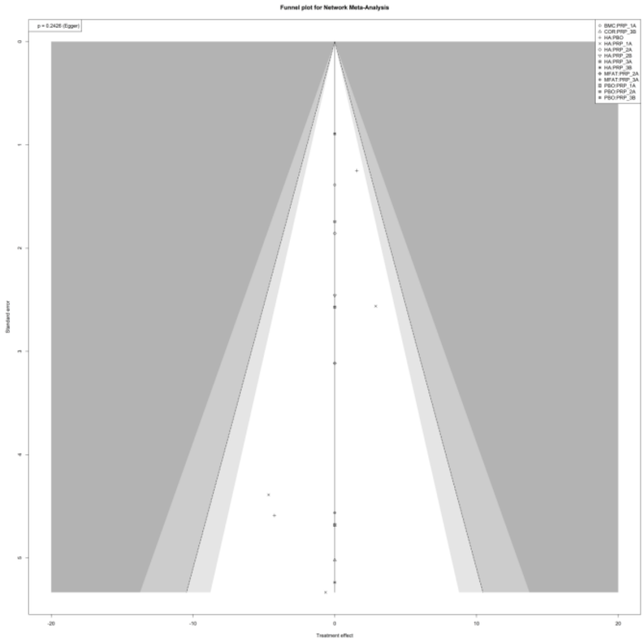

B)

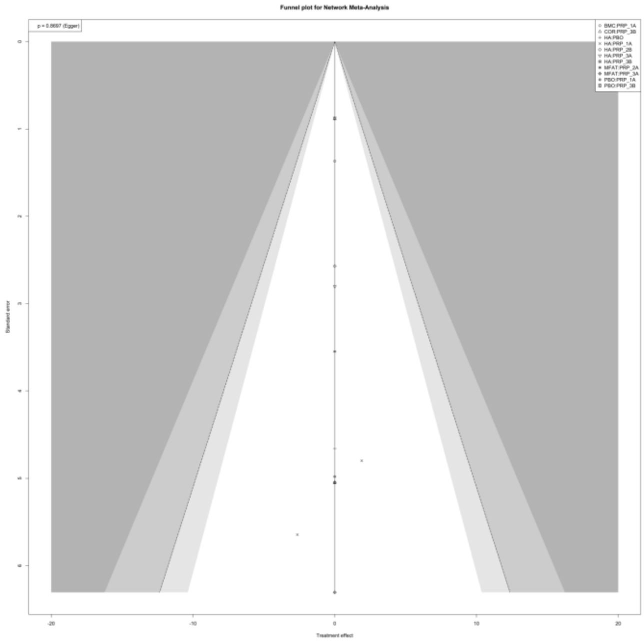

Figure S89. Contour enhanced funnel plot for trials of the effect of PRP categorized according to Mishra's classification system vs different non-surgical control treatments (BMC: Bone Marrow Concentrate, COR: Corticosteroids, HA: Hyaluronic Acid, MFAT: Microfragmented Adipose Tissue, PBO: Placebo, and SEP: Structured Exercise Program) on IKDC at the following time points: A) 6 months, and B) 12 months. The vertical line represents the pooled effect estimate. Contour lines indicate regions of statistical significance (*p*<0.01, *p*<0.05, and *p*<0.10). Asymmetry in the distribution of studies may suggest potential publication bias or small-study effects.

A)

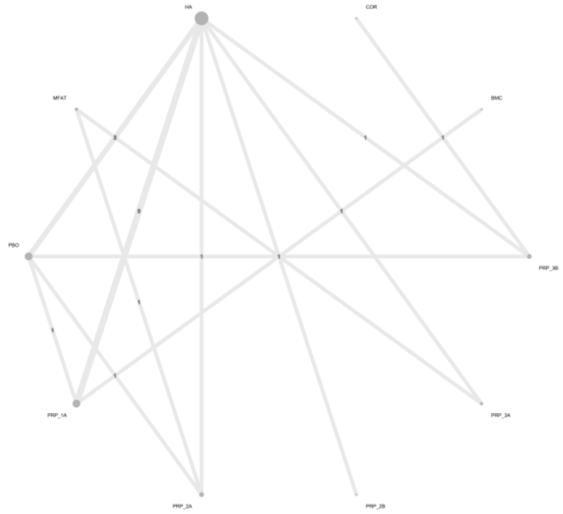

B)

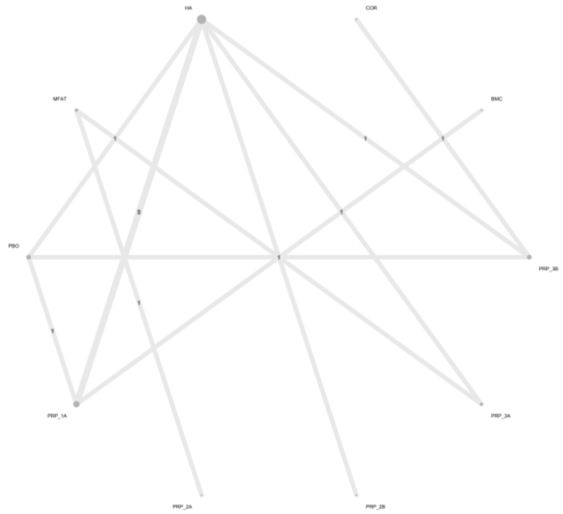

Figure S90. Network plot representing direct comparisons among interventions included in the network meta-analysis for IKDC at the following time points: A) 6 months, and B) 12 months. PRP treatments were categorized according to Mishra's classification system vs different non-surgical control treatments (BMC: Bone Marrow Concentrate, COR: Corticosteroids, HA: Hyaluronic Acid, MFAT: Microfragmented Adipose Tissue, PBO: Placebo, and SEP: Structured Exercise Program). Node size is proportional to the total number of participants receiving each intervention, and edge thickness reflects the number of studies contributing to each direct comparison.

A)

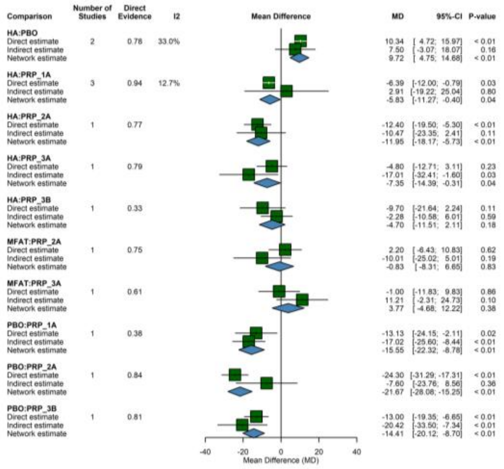

B)

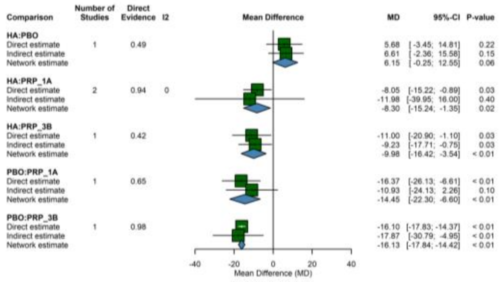

Figure S91. Node-split forest plot representing inconsistency assessment in the network meta-analysis of the effect of PRP categorized according to Mishra's classification system vs different non-surgical control treatments (BMC: Bone Marrow Concentrate, COR: Corticosteroids, HA: Hyaluronic Acid, MFAT: Microfragmented Adipose Tissue, PBO: Placebo, and SEP: Structured Exercise Program) on IKDC at the following time points: A) 6 months, and B) 12 months. Each treatment comparison includes both direct and indirect estimates for mean difference (MD) with corresponding 95% confidence intervals.

A)

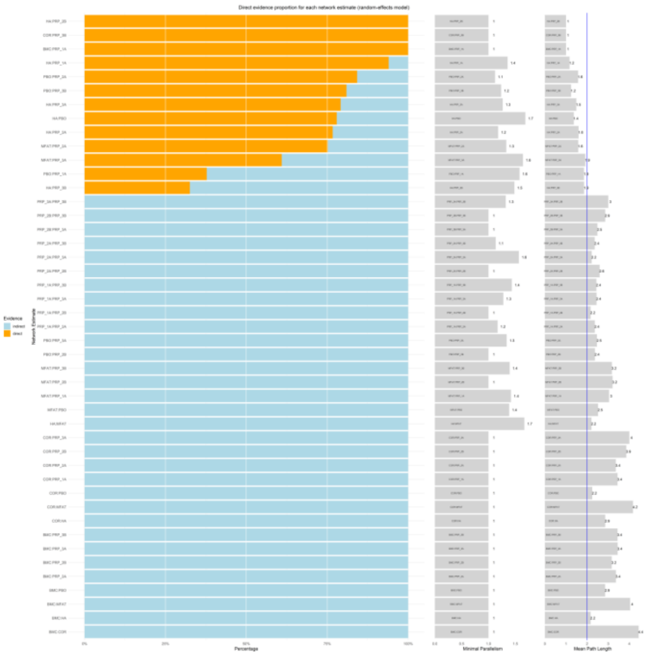

B)

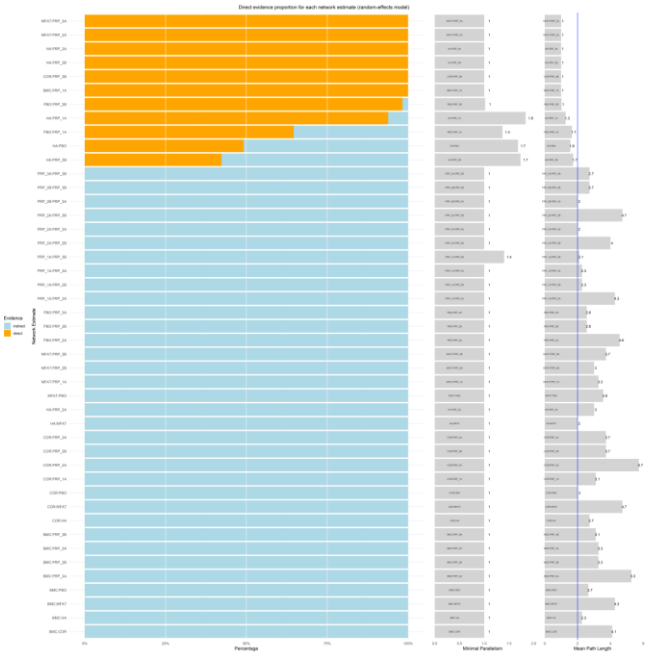

Figure S92. Direct evidence plot representing direct evidence proportion for each assessment corresponding to the network meta-analysis of the effect of PRP categorized according to Mishra's classification system vs different non-surgical control treatments (BMC: Bone Marrow Concentrate, COR: Corticosteroids, HA: Hyaluronic Acid, MFAT: Microfragmented Adipose Tissue, PBO: Placebo, and SEP: Structured Exercise Program) on IKDC at the following time points: A) 6 months, and B) 12 months. Key geometry metrics depicted include minimal path length, representing the shortest distance between nodes, and mean path length, reflecting the shortest parallel connections between nodes, and mean path length, quantifying the average shortest path across all pairs of interventions within the network.
